# From CRISPR–Cas to Argonautes: Defense systems of bacteria and archaea in the hydrothermal deep subsurface

**DOI:** 10.1093/ismeco/ycag200

**Published:** 2026-07-10

**Authors:** Lei Su, Paraskevi Mara, Virginia Edgcomb, Andreas Teske

**Affiliations:** State Key Laboratory of Marine Geology, Tongji University, Shanghai 200092, China; Department of Geology and Geophysics, Woods Hole Oceanographic Institution, Woods Hole, MA 02543, United States; Department of Geology and Geophysics, Woods Hole Oceanographic Institution, Woods Hole, MA 02543, United States; Department of Earth, Marine and Environmental Sciences, University of North Carolina at Chapel Hill, Murray Hall, CB 3300, Chapel Hill, NC 27599, United States

**Keywords:** defense genes, CRISPR–Cas, Argonautes, subsurface, Guaymas Basin

## Abstract

Microbial defense systems are central to virus–host interactions and thus to microbial survival but remain poorly studied in microbial communities of extreme environments. Here we examine the repertoire and distribution of microbial defense genes in hydrothermally influenced deep subsurface sediments and rocks of the Guaymas Basin (Gulf of California). Restriction–modification and abortive infection systems were broadly distributed across the examined sediment depths, and clustered, regularly interspaced short palindromic repeats–Cas systems were primarily detected within temperate surficial sediments. Prokaryotic Argonaute genes were found mostly in archaeal MAGs at elevated temperatures up to 81.8°C. Overall defense gene repertoire declines downcore, as temperature increases and phylogenetic host range narrows, with phylogeny as the decisive control factor. We suggest that these defense systems, together with DNA repair mechanisms, protein maintenance activities, and RNA modification pathways, constitute a survival toolkit for the hydrothermally influenced subsurface, where energy limitation and temperature extremes select for resilient microbial communities.

## Introduction

Microorganisms experience intense biological interactions in their natural environment, including constant exposure to viral predation. Recent estimates suggest there are nearly 10^31^ viral particles in the biosphere [1], and as many as 10^23^ infection events per second in the global ocean alone [2]. In response, prokaryotes have evolved diverse defense mechanisms, including restriction–modification (RM) systems that distinguish self from non-self DNA, and abortive infection (Abi) systems that limit viral propagation by sacrificing infected cells [3]. Defense systems also include adaptive immune systems, currently exemplified by clustered, regularly interspaced short palindromic repeats (CRISPR)–Cas systems. Collectively, the pan-immune system that includes these mechanisms has been termed the prokaryotic ‘immune system’ [4], which also includes additional newly discovered mechanisms that remain poorly understood [3].

Recently, a large-scale genomic survey identified at least 21 distinct anti-phage defense systems across nearly 18 000 bacterial and archaeal genomes and highlighted the modularity and complexity of these immune repertoires [5]. These systems include RMs, Abi systems, CRISPR–Cas machinery, and prokaryotic Argonaute (pAgo) systems. RMs are the most common defense systems identified in prokaryotic genomes. They involve endonucleases that cut foreign DNA at specific sequences and methyltransferases (MTases) that methylate the host DNA, protecting it from cleavage [6, 7]. Abi systems are post-infection mechanisms that include toxin–antitoxin (TA) modules inducing host programmed cell death or dormancy to limit phage propagation within a prokaryotic population [8–11]. pAgos are multi-domain systems involved in gene repression and defense against viruses and transposons [12, 13]. CRISPR–Cas defense systems provide acquired immunity by capturing short DNA sequences from mobile genetic elements and incorporating them into the host genome as spacers [14]. Different CRISPR–Cas types target DNA or RNA invaders through distinct molecular mechanisms [15].

This extensive repertoire of microbial self-defense genes has developed within microbial communities in natural environments. Here we demonstrate that they also extend to hydrothermal sediments and veined rocks in the marine deep biosphere of Guaymas Basin in the Gulf of California. The Guaymas Basin is a young marginal rift basin that is characterized by active seafloor spreading and rapid sedimentation, where organic-rich sediments, produced by high surface productivity and terrestrial runoff, alternate with extensive magmatic intrusions (doleritic sills) that penetrate laterally into the unconsolidated sediments [16]. The sediments and sills of Guaymas Basin were recently drilled during International Ocean Discovery Program (IODP) Expedition 385 (IODP 385) [17], to investigate how steep geothermal temperature gradients, changing porewater geochemistry, and variable sediment lithology shape bacterial and archaeal communities across a range of hydrothermal influence [18–20]. Integrated datasets show that cell abundance and DNA yield decline with increasing depth and temperature [21], matched by increasingly recalcitrant organic matter [22], diminished rates of anaerobic microbial processes downcore [23], and a progressive decline in the microbial remineralization of sedimentary organic matter, reflected in lower dissolved inorganic carbon and NH₄^+^ concentrations [17, 24, 25].

Previous metagenomic analyses of Guaymas Basin subsurface sediments collected during IODP Expedition 385 revealed the potential for diverse responses in microbial communities exposed to local environmental stresses [19]. Metatranscriptomic analyses showed expression of genes involved in DNA repair, protein maintenance, and RNA modifications, suggesting that physiological maintenance mechanisms remain active under hydrothermal conditions at different sites [18]. Additional adaptive mechanisms, such as microbial defense systems, likely also play a critical role in supporting microbial persistence. By mining previously generated Guaymas Basin metagenomes in combination with newly acquired metagenomes from additional IODP 385 sediments with strong hydrothermal influence, we explore the repertoire of microbial defense genes in these subsurface communities. While determining viral diversity and abundance specifically for Guaymas Basin sediments was outside the scope of this study, marine sedimentary systems show high viral endemism and abundance, the latter correlating positively with microbial abundance (10^7^–10^10^ viruses g^−1^ dry sediments) [26]. Thus, the downcore abundance and diversity of defense systems should follow microbial diversity and abundance trends in the Guaymas Basin subsurface. Placing Guaymas Basin within the emerging comparative framework of environmental defensomes [3] allows us to evaluate whether in hydrothermally influenced deep subsurface habitats microbial defense repertoires are shaped by both biological and environmental constraints. In demonstrating the potential of the deep subsurface as a promising hunting ground for microbial defense systems, we hope to encourage further metagenomic studies of the microbial defensome in extreme environments.

## Materials and methods

### Sampling

All samples for microbiology were handled aseptically and immediately flash-frozen in liquid nitrogen in 50 ml Falcon tubes and stored at −80°C (contamination control details in [20]). The interiors (excluding outer 2 cm of core) of frozen (−80°C) whole-round-core sections of deep sediments (Sites U1547 and U1548) were subsampled with sterile spatulas to obtain 0.5 g aliquots for DNA extraction (Table 1). A total of 0.5 ml of mudline fluids from the top of the first sediment core at IODP Hole U1546B, containing a mixture of seawater and finely suspended sediment particles, was subsampled for DNA extraction to assess the microbial composition of drilling fluids. Rock samples, preferentially with mineral-encrusted veins potentially allowing for hydrothermal fluid circulation, were crushed on the ship immediately after sampling using sterile hammers in a sterilized metal bowl in an anaerobic hood. This material was filled into 50 ml Falcon tubes and frozen at −80°C. Out of a wider range of veined rock samples subjected to DNA extraction, only one sample (1547D-10R1) yielded sufficient DNA for metagenomic sequencing (Table 1).

**Table 1:** Samples producing metagenomes.

**IODP site and hole**	**Core and section**	**Sample description**	**Depth [m], rounded**	**In-situ temp (°C), rounded**	**Cell numbers ml** ^**−1**^ **[21]**	**Publication of metagenomes**
U1547B	1H2	0.5 g sediment^*^	2.2	14.2	n.d.	[19]
U1547B	1H3	0.5 g sediment^*^	3.6	15.0	n.d.	[19]
U1547B	2H2	0.5 g sediment^*^	8.7	17.5	1.25 × 10^8^	[19]
U1547B	2H3	0.5 g sediment^*^	9.9	17.8	n.d.	[19]
U1547B	3H3	0.5 g sediment^*^	19.3	23.6	9.32 × 10^6^	[19]
U1547B	5H2	0.5 g sediment^*^	36.9	31.9	1.35 × 10^6^	[19]
U1547B	5H3	0.5 g sediment^*^	38.1	34.0	n.d.	[19]
U1547B	7H3	0.5 g sediment	57.4	42.3	3.2 × 10^5^	[19]
U1547B	8H2	0.5 g sediment^*^	65.8	46.6	4.3 × 10^4^	[19]
U1547B	9H2	0.5 g sediment	74.3	51.0	3.85 × 10^4^	[19]
U1547B	9H3	0.5 g sediment	76.0	51.9	n.d.	[19]
U1547D	10R1	Crushed veined rock, 20 ml^*^	134.5	81.8	n.d.	This publication
U1548B	2H3	0.5 g sediment^*^	8.9	13.7	9.96 × 10^7^	[19]
U1548B	4H7	0.5 g sediment^*^	33.5	33.5	1.78 × 10^7^	[19]
U1548B	8H5	0.5 g sediment	69.5	62.4	1.01 × 10^3^	[19]
U1548C	1H3	3 × 0.5 g sedim.^*^	2.78	18.6	n.d.	This publication
U1548C	3H6	3 × 0.5 g sedim.^*^	24.17	39.1	n.d.	This publication
U1548C	5H4	3 × 0.5 g sedim.^*^	40.38	54.6	n.d.	This publication

Overview of sediment and rock samples from Ringvent sites (U1547 and U1548) from previously published metagenomes [19], and newly extracted and sequenced samples included in this publication (1547D-10R1 crushed veined rock; 1548C-1H3, 3H6 and 5H4 sediment). Depths are given following IODP’s Core depth below Seafloor-A (CSF-A) terminology. Samples that yielded genes for defense systems are marked with an asterisk in the sample description column. Cell numbers and DNA concentrations for a wider range of Ringvent samples [21] are compiled in Table S3.

### DNA extraction

DNA was extracted from 0.5 g sediment, or pooled 3 × 0.5 g sediment samples using the MP Biomedical FastDNA™ SPIN Kit, with modifications for subsurface sediments [21]. For veined and crushed rock samples (Table S1), DNA extraction was performed with the MP Biomedical FastDNA™ SPIN Kit with additions and modifications (Supplementary Methods). DNA was shipped to the University of Delaware Sequencing and Genotyping Center (UDSG) for library construction (including Illumina Library Quality Analysis, Qubit quantification and Fragment Analysis, followed by ddPCR) and metagenomic sequencing using Element Aviti 2x150 base pairs High Output. Library construction and sequencing were internally fine-tuned at UDSG to allow sequencing of low-DNA samples containing <1 ng/μl DNA (Supplementary Materials) [21].

### Metagenomic processing, assembly, and binning of MAGs

Illumina adapters and low-quality sequences were removed from raw sequencing reads using the read_qc module in MetaWRAP v1.3.2 (https://github.com/bxlab/metaWRAP) [27]. Sequence quality before and after trimming was assessed using FastQC v0.12.1 (http://www.bioinformatics.babraham.ac.uk/projects/fastqc). High-quality reads from contamination control samples were assembled de novo using MEGAHIT v1.2.9 [28]. Subsequently, an index was constructed from the assembled control contigs using Bowtie2 (v2.5.4), and environmental sample reads were aligned against this contaminant sequence database to identify potential contaminants. Unaligned reads, representing non-contaminated sequences, were extracted using Samtools v1.17 and retained for downstream analyses. The resulting cleaned reads (Supplementary Results) were subjected to independent assembly using MEGAHIT with parameters --min-count 2 --k-min 41 --k-max 141 --k-step 10. Three metagenomic binning programs, MetaBAT2 (v2.12.1), MaxBin2 (v2.2.4), and CONCOCT (v1.1.0), were applied with default parameters to reconstruct metagenome-assembled genomes (MAGs) from the assembled contigs. To ensure the sample-specific resolution needed for our downstream temperature- and depth-resolved comparisons, multi-sample binning was avoided; the sample-specific binning strategy accepted a more conservative degree of defense system recovery for lower-completeness MAGs. The resulting genome bins were refined using the binning_refinement module in MetaWRAP to generate the final MAG set. The completeness and contamination of all MAGs were estimated using CheckM2 (v0.1.3) [29]. MAGs with >50% completeness and <10% contamination were retained for subsequent analyses. Taxonomic classification of refined MAGs was performed using the Genome Taxonomy Database Toolkit (GTDB-Tk) [30] with database release R226. To reduce genomic redundancy and establish a non-redundant set of representative genomes for downstream analysis, all refined MAGs were dereplicated using dRep (v3.4.3) with a secondary average nucleotide identity threshold of 95% and a minimum completeness of 50%. Genome coverage of dereplicated MAGs within each sample was calculated using CoverM (v0.7.0) [31].

### Functional annotation and defense system identification

Automated metabolic annotation of MAGs was performed using Prokka (v1.14.6) [32], and subsequently refined with DRAM (Version 1.4.6; Distilled and Refined Annotation of Metabolism) [33]. Additional genome annotation was carried out with METABOLIC (METabolic And BiogeOchemistry anaLyses In miCrobes) [34] for curated functional assignments relevant to microbial biogeochemistry. Antiviral defense systems in MAGs were identified using DefenseFinder v1.0.2 with the parameter “-dbtype gembase,” following MacSyFinder v1.2.0 models and associated rules [35].

### Spacer analysis of CRISPR–Cas systems

CRISPR spacer sequences were extracted from MAG contigs using MinCED (v0.4.2) (https://github.com/ctSkennerton/minced) with a minimum of 2 repeat units. Extracted spacers were queried against the IMG/VR database using BlastN (e-value ≤1 × 10^−5^, word size 7) to identify potential viral targets. Additionally, spacers were compared against locally assembled metagenomic contigs to identify potential local spacer targets, including viral, plasmid, prophage, mobile-element, or host-derived sequences. BLAST hits were filtered at ≥95% nucleotide identity and ≥95% query coverage. Per-MAG spacer counts and hit rates were summarized and merged with temperature and taxonomic metadata for downstream analysis.

### Statistical framework

Differences in MAG abundance (RPKM) and alpha diversity (Shannon index, calculated from normalized RPKM) among temperature groups were assessed using Kruskal–Wallis and pairwise Wilcoxon rank-sum tests with Benjamini–Hochberg (BH) correction for multiple comparisons. Effect sizes were estimated using Cliff’s delta with 95% confidence intervals. CRISPR–Cas prevalence across temperature bins was quantified and tested using Fisher’s exact test (global and pairwise where applicable) and logistic regression (CRISPR_Cas ~ temperature). To test taxonomy effects on defense repertoires, we used Kruskal–Wallis tests for repertoire size (number of defense categories per MAG) and PERMANOVA (*adonis2*; binary Jaccard distance) for multivariate defense composition. Homogeneity of multivariate dispersion was evaluated using PERMDISP (betadisper + permutest). Sample-level beta diversity of MAG community composition was assessed using Bray–Curtis dissimilarity calculated from RPKM abundance data (CoverM). Ordination was performed using both principal coordinates analysis (PCoA; cmdscale) and non-metric multidimensional scaling (NMDS; metaMDS with 100 random starts). The effects of temperature and depth on community composition were tested using PERMANOVA (*adonis2*, marginal tests, 999 permutations). Defense system occurrence was mapped against host MAG abundance across samples by linking defense presence/absence with RPKM-based relative abundance.

### Phylogenetic regression analysis

To disentangle phylogenetic effects from environmental (temperature) effects on defense system distribution, we performed phylogenetic comparative analyses on all 195 MAGs. A combined phylogenetic tree was constructed using UBCG (Universal Bacterial Core Genes, v3.0) [36], which extracts, aligns, and concatenates up to 92 universally conserved single-copy core genes across Bacteria and Archaea. The concatenated alignment was used to infer a maximum-likelihood tree with IQ-TREE3 under the best-fit substitution model selected by ModelFinder [37], with 1000 ultrafast bootstrap replicates [38] and 1000 SH-aLRT replicates. The tree was midpoint-rooted using the R package *phangorn* [39]. Phylogenetic generalized least squares (PGLS) regression was performed using the R package caper [40] with maximum-likelihood estimation of Pagel’s *λ* [41] to test whether temperature explains variation in defense gene count after accounting for phylogenetic non-independence. For binary defense system traits (presence/absence of RM, Abi, CRISPR–Cas, and pAgo), phylogenetic logistic regression was performed using phylolm [42] with the logistic_MPLE method. Models were run with temperature as the sole predictor and additionally with domain (Bacteria/Archaea) as a covariate (Supplementary Materials). To account for multiple testing, *P*-values from phylogenetic logistic regression were corrected using the BH false discovery rate (FDR) procedure. Only MAGs present in the phylogenetic tree, the temperature metadata, and the DefenseFinder output were included in the PGLS analysis.

### MAG assembly vs sequence-based community

To assess the fraction of community diversity not captured by MAG assembly, quality-filtered reads from all 18 samples were taxonomically classified using Kraken2 (v2.1.3) [43] with the standard database (containing RefSeq bacterial, archaeal, viral, human, and UniVec sequences). Bracken (v2.8) [44] was used to re-estimate taxon abundances at the phylum level with a minimum read threshold of 10. Phylum-level relative abundance matrices were generated for Kraken2 profiles and compared with GTDB-Tk-classified MAG abundance profiles (CoverM RPKM). Because GTDB and NCBI taxonomies use partially overlapping phylum nomenclature with different naming conventions (GTDB uses prefix notation, e.g. “p__Actinomycetota”), comparisons were interpreted at the level of shared phylum-level lineages rather than exact string matching. Beta diversity of Kraken2-based community profiles was assessed using the same Bray–Curtis/PERMANOVA framework as for MAG-based profiles.

### Phylogenetic analyses of Argonaute genes

Ago sequences of the Guaymas subsurface (Table S2) were queried for homologs in GenBank using BlastX [45]; further homologs were identified in curated Argonaute databases [46] and added. An overabundance of gaps in initial machine-generated protein alignments, combined with phylogenetic instability of the resulting trees (much impacted by species selection), motivated us to proceed with manually curated alignments using the alignment editor SeqPup [47]. As a first step, Argonautes and their homologs were compared via pairwise online alignments in BlastX and BlastP, and this was repeated in an iterative manner for alignment consistency. Guided by homologous domain sequences and conserved amino acid positions identified using the NCBI Conserved Domain Search Interface in BlastX and BlastP, consistent long-range alignments were obtained, one for sequences with C-terminal PIWI domains, and another alignment for sequences with N-terminal toll/interleukin receptors and acetylation lower-binding affinity (AlbA*)* DNA-binding proteins (Supplementary File 1). Attempts to unify the two alignments failed due to a lack of sequence homology. Sequence portions outside of BlastP-identified domains were excluded from the alignments. Protein distance trees were inferred in PAUP [48], based on mean character differences with minimum evolution as optimality criterion; phylogenies were checked by 1000 full heuristic bootstrap iterations.

### Protein structure of Argonaute sequences

Protein structure and function predictions of the nine Argonaute protein sequences were performed using Phyre2 [49, 50] (Supplementary pdf files). Amino acid sequences were submitted to the Phyre2 web server, which predicts three-dimensional protein structures by homology modeling against known structures in curated databases, including the detection of remote evolutionary relationships. Predicted structural models were used to infer putative protein functions based on structural similarity.

## Results

### MAG diversity and read recruitment decrease across hot sediments

**Figure 1:**
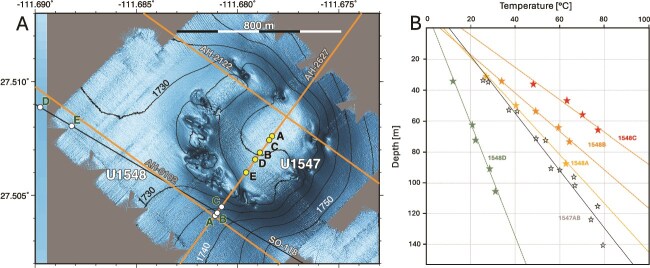
Overview on Ringvent sampling locations and their thermal gradients. (A) Bathymetry of Ringvent, with seismic lines from site survey expeditions (RV ‘Sonne’, July 2015; RV ‘Alpha Helix’, April 2016), and drilling locations for IODP Sites U1547 and U1548 [17]. (B) Temperature gradients for Ringvent drilling sites. Each numbered site includes multiple adjacent holes (identified by alphabetic suffix) along a transect toward increasing hydrothermal exposure. Temperature gradients steepen as drilling holes approach the hydrothermally active crest of Ringvent, with Hole 1548C being the closest and hottest. The temperature data points underlying interpolated in-situ temperatures (Table 1) and linearized temperature profiles are compiled in Table S4.

To investigate host defense systems within the hydrothermally influenced Guaymas Basin subsurface, we focused on the Ringvent area, characterized by shallow magmatic sill intrusions, steep thermal gradients, and active hydrothermal circulation [25, 51, 52]. We analyzed publicly available MAGs from cool (13.7°C–20°C), warm (20-45°C) and hot (45°C–62.4°C) sediments collected at sites U1547 Hole B (U1547B) and U1548 Hole B (U1548B) [19]. In addition, we generated additional MAGs from previously unexamined sediment and rock samples at Sites U1547 and U1548 (Table 1). Generally, Ringvent samples are characterized by rapidly declining cell numbers and DNA concentrations downcore (Table S3). Sediment samples from Hole U1548C, closest to the hydrothermally active crest of the Ringvent area (Fig. 1A), exhibited the steepest thermal gradient documented during IODP 385 (Fig. 1B, Table S4) and yielded sufficient DNA for new metagenomes. In addition to sediments, a sill rock sample collected from 134.5 meters below seafloor (mbsf) in Hole U1547D yielded sufficient DNA for metagenomic library construction and sequencing (Tables S1 and S2). The *in situ* temperature of 81.8°C was extrapolated from the temperature gradient in sediments immediately overlying the sill rock.

Seventeen sediment samples and one rock sample (Table 1) yielded 195 MAGs (>50% complete, <10% contamination) through independent assembly of each sample dataset (Table S5). Archaeal MAGs (*n* = 59) were affiliated with six phyla: Aenigmatarchaeota (*n* = 4), Hadarchaeota (*n* = 13), Halobacteriota (*n* = 1), Methanobacteriota_B (*n* = 1), Thermoplasmatota (*n* = 7), and Thermoproteota (*n* = 33). Bacterial MAGs (*n* = 136) were affiliated with 19 phyla, primarily Acidobacteriota (*n* = 10), Actinomycetota (*n* = 8), Aerophobota (*n* = 16), Bipolaricaulota (*n* = 6), Chloroflexota (*n* = 51), Desulfobacterota (*n* = 8), Planctomycetota (*n* = 9), and WOR-3 (*n* = 7) (Table S5).

**Figure 2:**
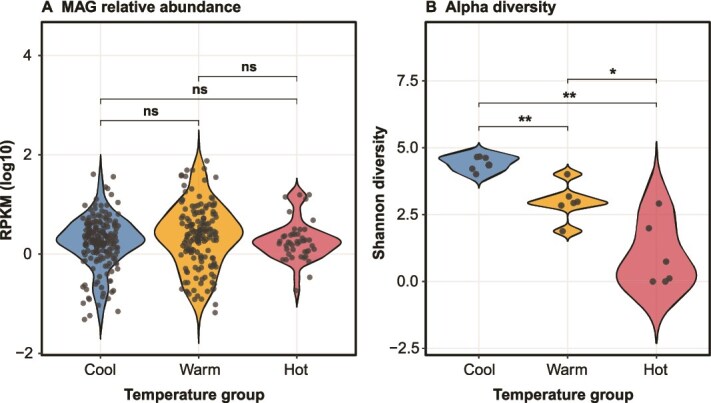
MAG relative abundance and alpha diversity across different temperature regimes (<20°C; 20°C–45°C; >45°C; [19]). (A) Violin plots of distribution and abundance of MAGs measured in reads per kilobase per million mapped reads (RPKM) and displayed on a log₁₀ scale. Each point denotes one MAG; violin widths reflect kernel density estimates. (B) Violin plots of alpha diversity (Shannon index) calculated for each sample based on the corresponding MAG abundance profile. Each point represents one sample. For both panels, pairwise differences between groups were performed using Wilcoxon rank-sum tests, with BH correction for multiple comparisons. Significance is indicated as *P* < .05 (^*^), *P* < .01 (^**^), and *P* < .001 (^***^).

**Figure 3:**
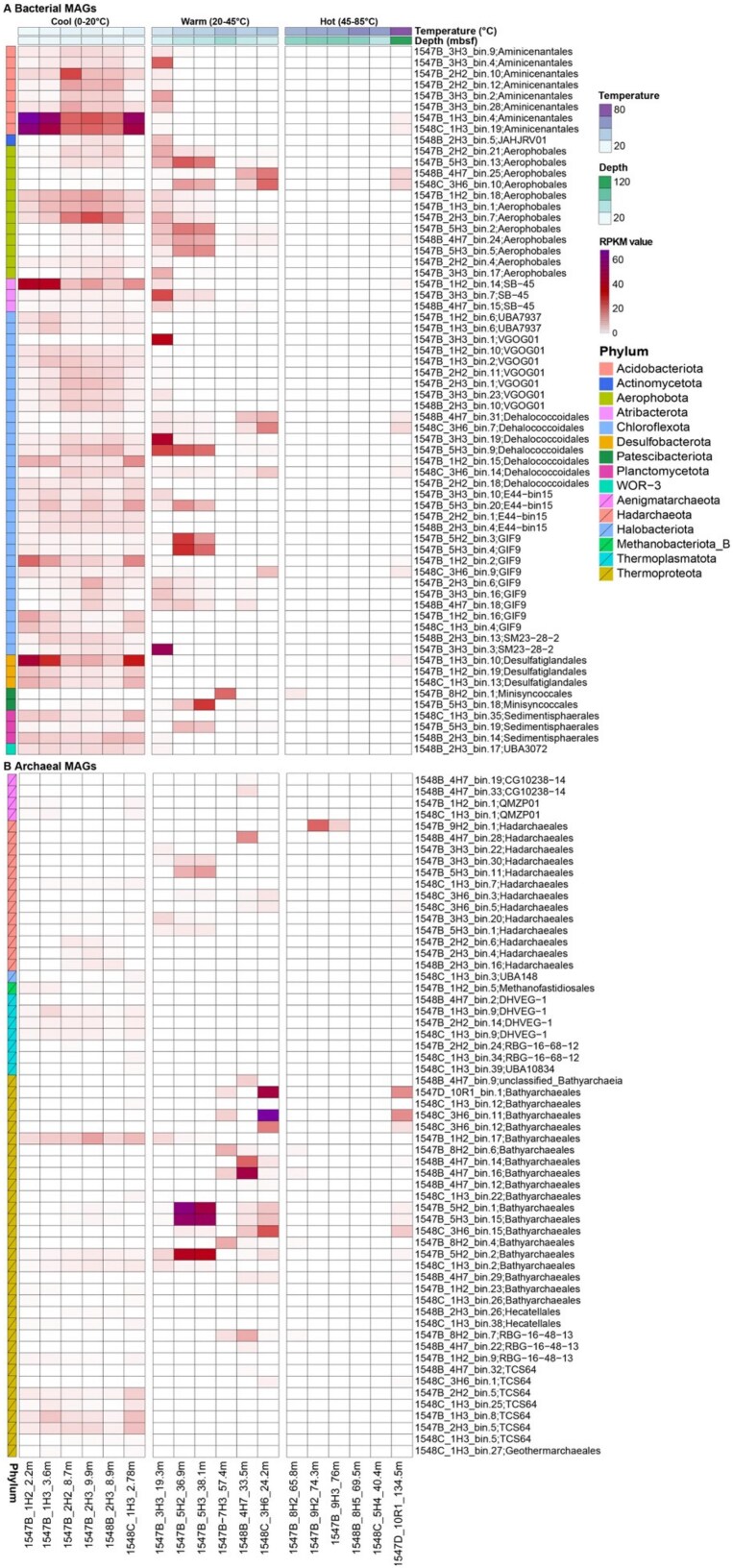
Overview heatmap of bacterial and archaeal MAG distribution on the Ringvent sites U1547B, U1547D, U1548B, and U1548C with temperature classes (<20°C; 20°C–45°C; >45°C; [19]) and by depth. Each column shows the percentage of total pre-processed metagenomic reads (relative abundance) that mapped to all 195 MAGs, for samples ordered by increasing temperature from left to right on the *x*-axis (annotated by site numbers and depths in mbsf). Temperature classes are separated by vertical dashed lines. Each row shows the abundance profile of an individual MAG across all samples, based on metagenomic sequence recruitment to individual MAGs. MAGs are color-coded by phylum on the left, and order-level affinity to the right. RPKM, reads mapped per kilobase of genome, per million mapped reads. Panel section A denotes bacterial MAGs and panel section B denotes archaeal MAGs. Relative MAG abundances are provided in the Source Data file.

We considered whether MAG recruitment, diversity, and taxonomic range would change according to thermal regime, cool = 3°C–20°C, warm = 20°C–45°C, hot >45°C, as defined previously [19]. Checked by pairwise Wilcoxon comparisons, MAG read recruitment did not change significantly across cool, warm, and hot temperature ranges (Fig. 2A), with the caveat that the median in MAG read recruitment for the hot samples was lower than those for cool and warm samples (Table S6). Alpha diversity based on MAG abundance profiles was significantly higher in cool and warm sediment samples compared to hot sediments (Fig. 2B), consistent with reduced cell densities (Table S3) and reduced taxonomic diversity downcore (Fig. 3). The reduced median for MAG recruitment in hot samples compared to cool and warm samples (Table S6) suggests that a larger share of the metagenomic sequences could not be assembled into MAGs at high temperatures, indicative of increasingly fragmented sequences. To check the extent of MAG recruitment, Kraken2/Bracken taxonomic profiling of quality-filtered reads from all 18 samples showed several major phylum-level sequence groups that were present in the read-level community profiles but were not recovered as MAGs (Fig. S1). Implicitly, MAG recruitment provides only a partial window into the existing microbial diversity (see “Metagenomic profiling before MAG assembly” in Supplementary Materials).

A taxonomy heatmap of MAG sequence recruitment, with samples sorted by increasing temperature (Fig. 3), shows how recruitment to highly diverse bacterial and archaeal MAGs in cool sediments undergoes selection toward a narrower phylogenetic range in warm and hot samples, until only a small number of bacterial (Aerophobota, Dehalococcoidia) and archaeal MAGs (Bathyarchaeota, Hadarchaeota) recruit any reads in samples above 45°C. This tendency toward taxonomically restricted MAG recruitment becomes stronger as overall downcore microbial cell numbers decline exponentially. Compared to abundant microbial communities in surficial sediments (approx. 10^8^ to 10^9^ cells/ml), cell numbers have dropped by several orders of magnitude near the 40°C–50°C temperature horizon (Table S3).

### Defense system diversity and distribution

**Figure 4:**
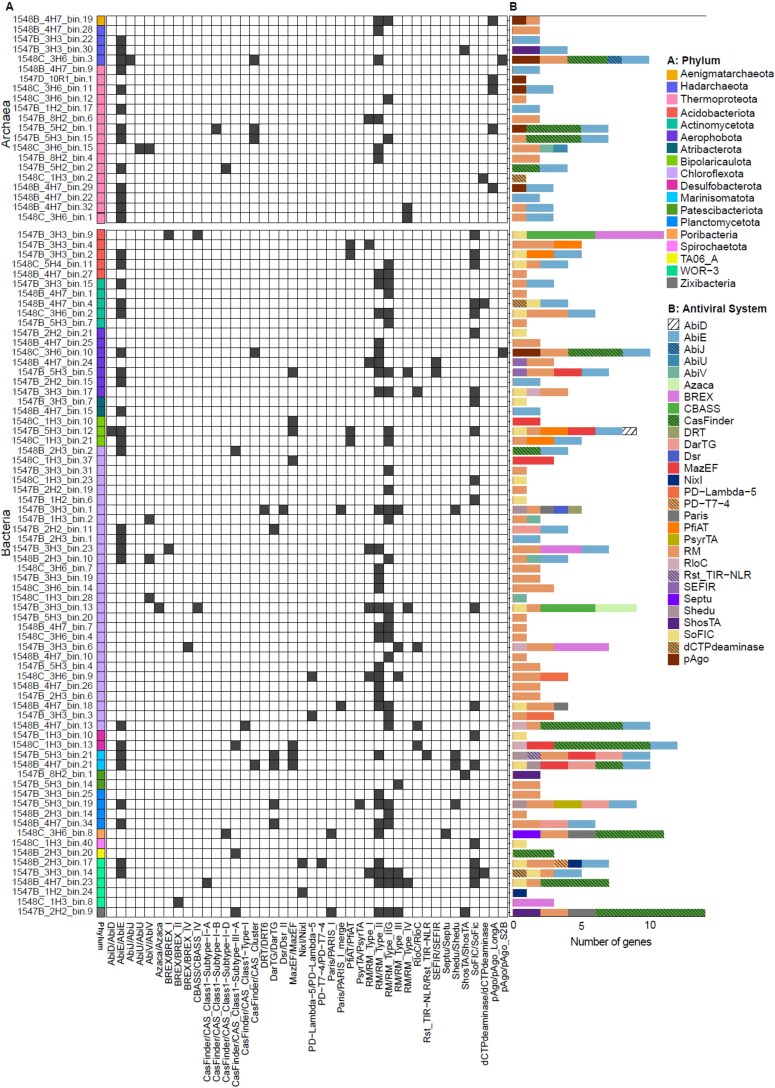
Identification and distribution of prokaryotic defense systems in MAGs recovered from sediment cores and veined rock samples collected at Ringvent sites U1547 and U1548, spanning depth from ~2 mbsf and to up to 134.5 mbsf and temperature regimes reaching up to 81.8°C.

Our analysis identified 353 antiviral genes distributed across 91 of the 195 reconstructed MAGs (Table S7), encompassing 42 families of defense systems (Table S8). Most defense-associated genes were recovered as fragmented loci rather than complete operons, consistent with the fragmented nature and variable completeness of the MAG dataset. Most defense systems were associated with RM systems, Abi systems, CRISPR–Cas systems, and pAgo genes. Recovered prokaryotic MAGs encoded various combinations of these defense systems across depths ranging from 2.2 to 65.8 mbsf, and at temperatures up to 54.6°C (Fig. 4; Table S8). RM and Abi systems are encoded in the majority of the bacterial and archaeal MAGs, ranging from shallow sediments (~2 mbsf) to deeper horizons at 65.8 mbsf, and spanning warm and hot temperature regimes up to 54.6°C (Fig. 4). The occurrence of specific defense systems differed across samples. Given the taxonomic composition of potential hosts changes with depth, temperature and host phylogeny likely play roles in shaping the defense systems present. Notably, MAGs from the veined rock sample (134.5 mbsf; 81.8°C) encoded only one defense system—pAgo, discussed in detail below.

**Figure 5:**
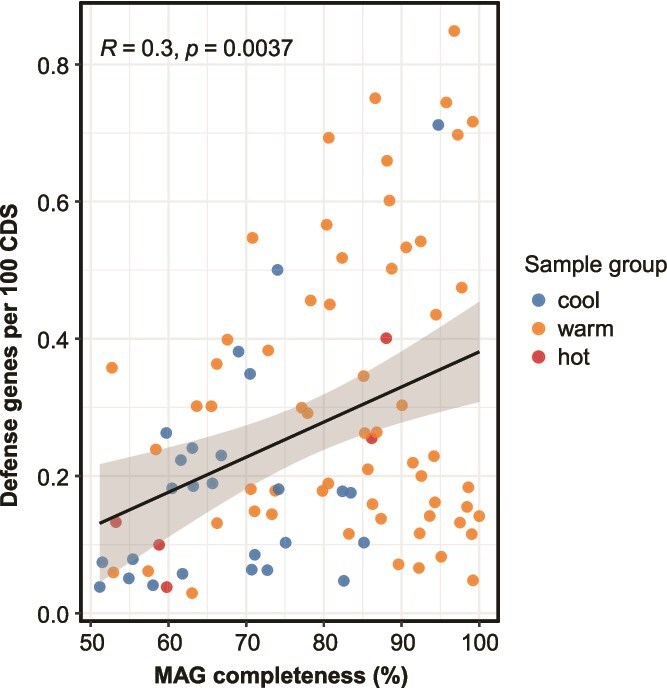
Correlation (*R* = 0.3, *P* < .0037) between MAG completeness and density of defense genes (number of defense genes per 100 coding sequences). MAG data points are color-coded by temperature (<20°C; 20°C–45°C; >45°C). Confidence intervals (95%) are shown as the shaded areas.

To assess whether the distribution of defense systems is biased by genome fragmentation, we examined the relationship between MAG completeness and normalized defense gene density (number of defense genes per 100 coding sequences, CDS) (Fig. 5). CDS count provides a functionally relevant measure of gene content that is less sensitive to assembly-related variation in intergenic and non-coding regions among MAGs of different completeness. In incomplete MAGs, total assembly length can vary substantially due to fragmented contigs and uneven recovery of non-coding sequences, which would make genome-size normalization less comparable across bins. By normalizing defense gene counts per 100 CDS, we aimed to compare defense gene investment relative to the recovered functional gene repertoire of each MAG. MAG completeness (≥50%) and the recovery of defense genes (*R* = 0.3, *P* < .0037) are linked by a weak but significant correlation (Fig. 5), consistent with the expectation that higher genome completeness increases the likelihood of detecting more complete defense systems. However, when focusing on MAGs with high genome completeness (≥80%) and normalized defense gene density, variation in the number of defense genes appeared more closely associated with taxonomic affiliation—particularly at the class and order levels—than with genome completeness itself (Table S7).

To validate whether taxonomic affiliation is indeed associated with the number of defense genes, we performed formal tests of taxonomic effects on defense repertoires using the corrected 18-sample dataset (91 MAGs with defense systems). Kruskal–Wallis testing (n_defense ~ phylum) showed that defense repertoire size does not differ significantly across phyla (χ^2^ = 6.26, df = 9, *P* = .714). However, PERMANOVA on defense system composition (binary Jaccard) showed a significant phylum effect (*R*^2^ = 0.162, *P* = .001); thus, defense repertoire composition (in contrast to size) is significantly structured by phylum affiliation. Temperature was not significant in the joint model (temperature *P* = .571; phylum *P* = .001).

### Phylogenetic vs temperature control of defense gene distribution

To disentangle phylogenetic effects from environmental (temperature) effects on defense system distribution, we constructed a combined maximum-likelihood phylogeny of all MAGs. We then separated the effect of phylogeny on defense system distribution from other factors (temperature) using PGLS, a statistical method that analyzes trait-environment relationships while accounting for their shared evolutionary history. PGLS analysis of total defense gene count revealed a near-zero phylogenetic signal (Pagel’s λ ≈ 0) and no significant temperature effect (coefficient = 0.0053, *P* = .343), indicating overall defense gene number is not directly driven by temperature after phylogenetic correction (Supplementary Results). Phylogenetic logistic regression of individual defense system categories showed consistent results across all categories. RM, Abi, and “Other” systems showed nominally positive associations with temperature in temperature-only models (*P* = .012–0.016), but all failed BH FDR correction (FDR ≈ 0.052). All effects disappeared when domain (Bacteria vs. Archaea) was included as a covariate (*P* = .305–0.919), indicating that the apparent temperature associations reflect domain-level phylogenetic structure. CRISPR–Cas systems showed no significant association with temperature in either model (*P* = .222/0.190). Finally, pAgo systems showed no significant temperature association (*P* = .368/0.202), although inference for pAgo remains limited by the small sample size (Supplementary Results). Thus, no individual defense system category shows a statistically significant temperature-dependent distribution, and observed patterns across temperature bins (Fig. S2) are primarily attributable to domain-level (bacteria vs. archaea) phylogenetic structure rather than direct environmental selection by temperature.

### Defense-system occurrence across MAG abundance gradients

**Figure 6:**
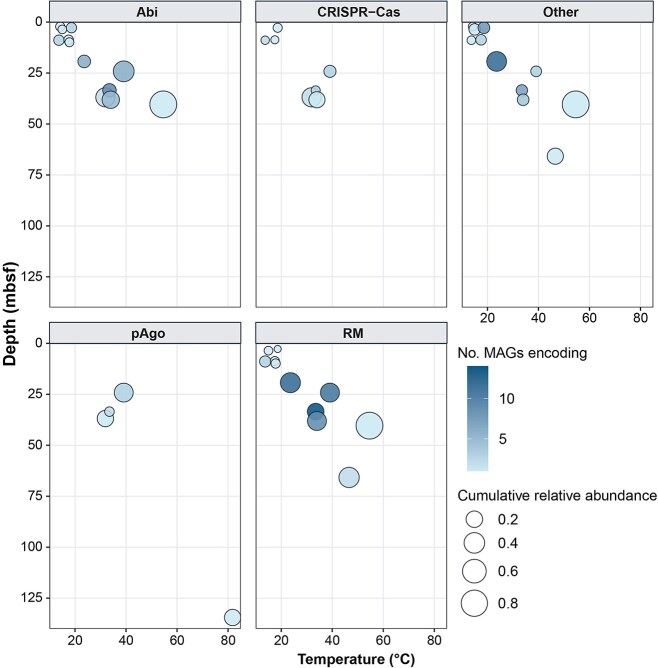
Depth and temperature distribution of defense system occurrence across Guaymas Basin subsurface communities. Each panel represents a distinct defense system category: RM (restriction–modification), Abi (abortive infection), CRISPR-Cas, pAgo (prokaryotic Argonaute), and Other. Data points represent individual samples containing defense genes (*n* = 14 samples spanning 2.2–134.5 mbsf and 13.7°C–81.8°C); the *x*-axis shows *in situ* temperature and the *y*-axis shows sediment depth, increasing downward. Bubble size indicates the cumulative relative abundance (sum of RPKM values across all MAGs encoding that defense system in a given sample); bubble shading indicates the number of MAGs encoding that defense type detected per sample. RPKM, reads per kilobase per million; mbsf, meters below seafloor.

To determine whether increased numbers of defense systems in a MAG lead to increased fitness (increased relative abundance) of any organism, we performed beta diversity analyses of MAG community composition across all 18 samples using Bray–Curtis dissimilarity calculated from RPKM abundance data. NMDS ordinations showed moderate visual clustering by temperature but no statistically significant separation (Fig. S3). PERMANOVA (marginal tests, 999 permutations) confirmed that neither temperature (*R*^2^ = 0.072, *F* = 1.245, *P* = .231) nor depth (*R*^2^ = 0.064, *F* = 1.100, *P* = .330) individually explained a significant fraction of community variation (Residual *R*^2^ = 0.872). This is consistent with MAG-level analyses showing taxonomy as the primary driver of defense repertoire composition. By mapping defense system occurrence against host MAG relative abundance across depth- and temperature-sorted samples, RM and Abi systems are often observed in relatively abundant MAGs, while CRISPR–Cas and pAgo are associated with lower-abundance hosts (Fig. 6). When checking the presence of defense systems vs MAG relative abundance for Abi, CRISPR–Cas, pAgo, RM and “Other” systems, it turns out that the absence or presence of defense genes in MAGs does not influence MAG abundance in our sample set (Fig. S4).

### Restriction–modification enzyme and abortive infection systems

RMs, in particular endonucleases and methyltransferases, were identified primarily in bacterial, and to a lesser extent, archaeal MAGs. Their broad distribution extended from shallow (~2 mbsf) to deeper sediment horizons at 65.8 mbsf, spanning temperature regimes up to 54.6°C. RM genes were detected in nearly all recovered genomes except for bacterial taxa associated with TA06, Spirochaetota, Blakebacterota, and Atribacterota, which were represented by relatively few MAGs (Fig. 4 and Table S8). The identified RM systems comprised five RM variants (I, II, III, IV, and subtype IIG). Most MAGs encoded more than one RM type, with the most frequent combinations involving Type II, subtype IIG, and Type III (Table S8). In contrast, RM Types I and IV are comparatively rare in our MAG dataset as are other types of Abi systems (AbiV, AbiU, AbiJ). RMs were not detected from sediment depths greater than 70 mbsf despite temperatures not exceeding ~51°C (i.e. samples U1547B_9H2 and 9H3; Table 1).

Among identified Abi systems, AbiE was the most prevalent, detected in both bacterial and archaeal MAGs. Similar to RM systems, AbiE occurred in MAGs from shallow sediments (~2 mbsf) down to 65.8 mbsf, spanning temperatures between 13.7°C and 54.6°C. AbiE antiviral systems were particularly abundant in archaeal and bacterial MAGs affiliated with Thermoproteota (Bathyarchaeales), Hadarchaeota (Hadarchaeales), Acidobacteriota, Actinomycetota (limited to the class Humimicrobiia), Aerophobota, Atribacterota, Bipolaricaulota, Chloroflexota (*Anaerolineae* and a single Dehalococcoidia MAG), Desulfobacterota, Marinisomatota, Planctomycetota, Gammaproteobacteria, and WOR-3 (Fig. 4; Table S8). One Hadarchaeota MAG recovered from sample U1548C_3H6 (24.2 mbsf; 39.1°C) (Table S7; Fig. 3) encoded both the AbiJ and AbiE systems. AbiU and AbiV were detected in one MAG affiliated with Thermoproteota (Bathyarchaeales) from sample U1548C_3H6 (24.2 mbsf; 39.1°C). The AbiV system was also encoded in three Chloroflexota MAGs (*Anaerolineae*) recovered from shallow Ringvent sediments (2.2–8.9 mbsf; 14.2°C). We did not recover Abi systems in MAGs from deep (>70 mbsf) or hot (62.4°C) Ringvent sediments or the rock sample. Other abortive systems that target phage DNA and host DNA to prevent phage replication (BREX, Paris, CBASS, and Septu; [5, 53, 54]; Table S7), were identified in 15 bacterial MAGs, primarily affiliated with Chloroflexota (*Anaerolineae* and Dehalococcoidia), recovered from shallow, warm (13.7°C–23.6°C) sediment samples (Table S7).

### CRISPR–Cas systems and spacer matches

The dominant CRISPR–Cas systems identified in the Guaymas Basin subsurface belonged to Class I, subtypes I, III, and IV (Fig. 4 and Table S9). Subtypes I and IV recognize, target, and cleave DNA, whereas CRISPR–Cas subtype III cleaves RNA (see below) and single-stranded DNA [55]. Subsurface bacterial and archaeal lineages encoded up to four genes from the CRISPR–Cas loci (*cas1–cas10*) and at least one *csc* gene (*csc1* or *csc2*), a component of the cascade complex (CRISPR-associated complex for antiviral defense) involved in recognizing and degrading viral or exogenous DNA (Table S9). CRISPR–Cas and associated genes were detected primarily in shallow to intermediate subsurface sediments (2.8–38.1 mbsf) at temperatures below 39.1°C. Effector genes associated with the CRISPR–Cas subtype III, such as *csm* (*csm*1–5) and the *csx1* genes that act selectively on single-stranded RNA [56–58] were identified in four prokaryotic MAGs from shallow cool Ringvent sediments (2.8–8.9 mbsf; 13.7°C–18.9°C), including a MAG from the Desulfatiglandales, a lineage of potentially hydrocarbon-degrading sulfate-reducing bacteria (Table S9). When quantified across temperature bins, CRISPR–Cas prevalence was 19.0% in MAGs from <20°C samples, 23.1% in MAGs from 20°C–45°C samples, and 8.3% in MAGs from >45°C samples. However, this apparent distribution was not statistically significant based on Fisher’s exact test (*P* = .166) or logistic regression (*P* = .254), indicating that CRISPR–Cas occurrence was not independently associated with temperature in this dataset.

CRISPR spacer analysis provided an indirect window into potential virus–host and mobile–element interactions in these samples. We identified 577 CRISPR spacers in 141 MAGs (112 bacteria, 29 archaea). These were queried against both the IMG/VR viral database and our assembled metagenomic contigs (requiring BlastN identity ≥95% and ≥95% query coverage). Only 15 spacers (2.6%) matched known viruses in IMG/VR. These were derived from 32 unique viral genomes across 7 MAGs from samples with *in situ* temperatures of 17.5°C–81.8°C. In contrast, 394 spacers (68.3%) matched local metagenomic contigs across 121/141 MAGs (85.8%). The mean self-hit rate per MAG was 64.9% (2335 unique self-target contigs). This low match rate to known viruses relative to the extensive local targeting indicates deep subsurface viral diversity is severely underrepresented in current databases, while also suggesting contributions from local mobile elements and possible self-targeting loci.

### Detection and phylogeny of pAgo genes

pAgo sequences were identified only in MAGs recovered from warm sediment samples (31.9°C–39.1°C) and the hot, veined rock sample (81.8°C) (Table S2). Argonaute systems were primarily found in archaeal lineages including Thermoproteota (Bathyarchaeia) and to a lesser extent in Aenigmatarchaeia and Hadarchaeota MAGs. Bacterial pAgos were obtained from an Aerophobota MAG. The predominant archaeal pAgo subtype was the long variant pAgo (pAgo_LongA), and the pAgo_LongB and pAgo_LongS1B variants were present in the Aerophobota MAG (Fig. 4 and Table S8).

**Figure 7:**
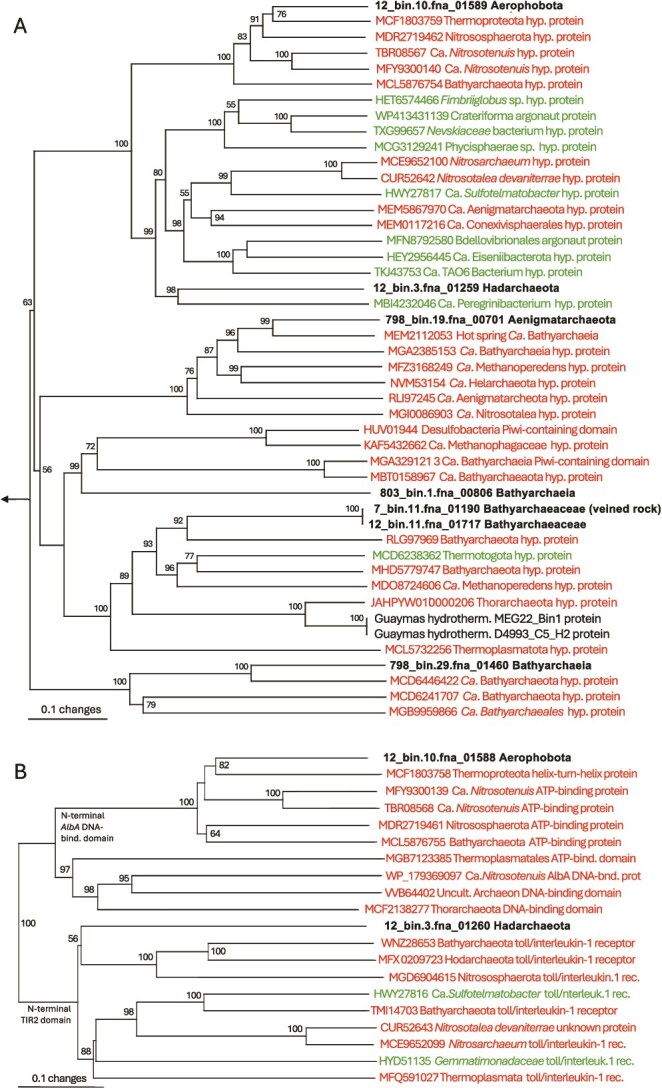
Minimum evolution phylogeny (averaged distances) of Argonaute protein sequences and closest GenBank matches identified with BlastX and BlastP, and in curated databases [46]. Archaeal database genes are highlighted in red, bacterial database genes in green. Taxon labels retain the taxonomic annotation of their GenBank entries. SRR suffixes (Table S2) are abbreviated to the last three numbers. Node stability was checked by 1000 full heuristic bootstrap iterations. (A) The phylogeny of C-terminal Piwi domains (Piwi_piwi-like_ProArk domains) was rooted with the deeply-branching HrAgo1 gene of the Asgard archaeon Ca. Harpocratesius repetitus [12]. (B) The phylogeny of N-terminal *AlbA* DNA-binding domains and N-terminal TIR2 domains was rooted at midpoint.

In addition to removing reads that mapped to a contamination control, we excluded gamma- and alphaproteobacterial pAgos related to human skin epibionts and tap water bacteria—repeatedly flagged as contaminants [59]—from our focused phylogenetic analysis of pAgo sequences. Nine remaining pAgo sequences were analyzed with their best available matches from GenBank (Table S2); mostly archaeal homologs within the Thermoproteota, in particular Bathyarchaeia and Nitrososphaeria (Fig. 7). Of the nine pAgo sequences, seven contained a C-terminal PIWI domain, specifically the Piwi_piwi-like_ProArk domain found in Archaea and Bacteria [60] (Fig. 7a). Two pAgo sequences belonged to clusters of N-terminal toll/interleukin receptors (TIR2) and of N-terminal DNA-Binding domains, identified as members of the *AlbA* family [61] (Fig. 7b).

Two identical bathyarchaeal sequences with C-terminal PIWI domains were recovered from the veined rock sample (U1547D_10R1, 7_bin.1.fna_01190) and the sediment sample (1548C_3H6, 12_bin.11.fna_01717), indicating these sequences share the same bathyarchaeal source organism that occurs in both samples. Implicitly, this source organism tolerates elevated temperatures (81.8°C for the rock sample, 39.1°C for the sediment sample). Bathyarchaeia and Jordarchaea were the closest taxonomic matches identified via GenBank BlastP queries. To further explore potential archaeal matches, we compared these bathyarchaeal subsurface pAgo genes against a curated database generated from samples of shallow hydrothermal sediments in Guaymas Basin [46]. This search yielded two identical shallow sediment sequences, D4993_C5_H2 protein and MEG22_Bin1 protein (Fig. 7a). Other archaeal pAgo sequences from Ringvent holes U1547B and U1548B, including the relatively short pAgo SRR22580798_bin.29.fna_01460 (Bathyarchaeia), pAgo SRR22580798_bin.19.fna_00701 (Aenigmarchaeota), and pAgo SRR22580803_bin.1.fna_00806 (Bathyarchaeia), all formed separate lineages along with their primarily archaeal database matches. Nevertheless, these pAgo sequences shared the C-terminal PIWI domains with the hot veined rock pAgo sequence (7_bin.1.fna_01190) and the sediment pAgo (12_bin.11.fna_01717), indicating a common phylogenetic root (Fig. 7a).

In several cases, it would be hard to infer the source of Argonaute sequences only based on homologs from databases. For example, the Hadarchaeotal sequence 12_bin.3.fna_01259 from Ringvent site 1548C (39.1°C) lacked close matches to sequences from other samples, and was only distantly affiliated to bacterial and archaeal counterparts (Ca. Peregrinibacter and Bathyarchaeia) (Fig. 7a). Similarly, the pAgo sequence SRR22580803_bin.1.fna_00806 from a Bathyarchaeia MAG at site U1547B (36.9 mbsf, 31.9°C) lacked close database matches and was positioned as a lineage basal to Argonaute genes from Bathyarchaeia, Methanophagales and Desulfobacteria (Fig. 7a), leaving the source organism uncertain. Inferences based solely on database matches can lead to erroneous conclusions, as shown by sequences 12_bin.10.fna_01589 and 12_bin.10.fna_01588 (39.1°C, U1548C), representing different protein domains (C-terminal Piwi_piwi-like_ProArk domain versus N-terminal *AlbA* family DNA-binding domain) but coming from the same bin. Both sequences share the same archaeal homologs (*Nitrosotenuis* sp. TBR08567 and TBR085568; *Nitrosotenuis* sp. MFY9300140 and MFY9300139; Thermoproteota MCF1803759 and MCF1803758; Bathyarchaeota sp. MCL5876754 and MCL5876755), suggesting a shared archaeal source. However, these Argonaute sequences come from a bacterial MAG affiliated with Ca. Aerophobota; thus, the consistently observed archaeal homologs indicate lateral gene transfer from archaea into Ca. Aerophobota.

## Discussion

### Defense genes: bonus or burden?

The diverse and ubiquitous cellular defense mechanisms present in Guaymas Basin subsurface sediments defend microbial cells against viral infection, which otherwise would compromise cellular integrity and divert a substantial fraction of the host’s ATP away from cellular maintenance and growth in favor of viral replication [62, 63]. Such defenses are likely very important for microorganisms living in habitats where occasional or routine energy limitation presents a challenge to survival. On the other hand, chronic energy limitation in the deep biosphere [64] selects for smaller, more easily maintained genomes in deep, hot Guaymas sediments [19]. Energy-limited microorganisms may favor persistent defense strategies that balance genome economy with the long-term benefits of viral protection [3]. This trade-off may help explain the prevalence of RM and Abi systems, which provide constitutive protection using a small number of genes and do not require spacer acquisition or immune memory, unlike adaptive CRISPR–Cas systems, which require spacer acquisition, maintenance of diverse CRISPR arrays, and ongoing adaptation to changing viral populations.

### CRISPR–Cas diversity and function

CRISPR–Cas systems and associated *cas* genes identified in our MAGs were primarily subtypes I and IV, which are known to recognize and interfere with invading DNA from phages or plasmids [55]. CRISPR–Cas subtype III and associated *csm* and *csc* genes targeting RNA and single-stranded DNA were detected only in four bacterial MAGs affiliated with Chloroflexota, Desulfobacterota, TA06, and Zixibacteria. All targeting systems were observed in MAGs from shallow to intermediate subsurface sediments (2.8–38.1 mbsf) at Ringvent under temperatures not exceeding 39.1°C. CRISPR–Cas systems are broadly distributed in marine bacterial and archaeal genomes [55, 65], with *cas* genes showing high prevalence in thermophilic and hyperthermophilic prokaryotic lineages [66]. As above, MAG incompleteness may hinder detection of CRISPR–Cas systems in some samples within our MAG dataset. In contrast, RMs and Abi systems (below) occur across a broader range of depths and temperature regimes (up to 65.8 mbsf and up to 54.6°C). These distribution patterns for distinct defense mechanisms may not only reflect varying thermal and geochemical conditions but also differences in viral diversity across sediment temperatures and depths. In addition, we hypothesize that CRISPR–Cas systems at shallow and intermediate subsurface depths may serve additional functional roles, such as host self-targeting, gene regulation, or chromosome rearrangement (see Supplementary Discussion). Published metatranscriptome analyses showed expression of genes associated with genome editing in the deep biosphere of Guaymas Basin [18].

### RM and Abi systems

For the Guaymas Basin subsurface, metagenome data suggest RM and Abi systems are the primary defense against exogenous DNA, as they were detected in MAGs across a wide range of temperatures, including sediments up to 54.6°C. The function and possible combined action of RM and Abi systems, specifically of restriction enzymes required for RM systems or the expression of toxins employed by Abi systems [67], is consistent with their shared presence in samples below 54.6°C. Typically, RM systems act during early stages of infection, whereas Abi systems function at later stages [68]. Abi systems are commonly found in prokaryotic genomes [5], and induce programmed cell death in the host, limiting phage propagation within prokaryotic populations [8]. Thus, tight regulation of Abi systems is required to trigger host cell death only after phages have evaded other intracellular defenses [68]. Many taxa in the Guaymas dataset encode at least one Abi and one RM system, or a combination of different RMs and one Abi systems, consistent with prokaryotic genomes that commonly encode both RM and Abi systems.

RM systems are the simplest and most abundant defense mechanisms in prokaryotic genomes and are categorized into four main types (I, II, III, and IV), encompassing multiple subtypes (e.g. subtype IIG) [6, 69]. The most common combination of RM and Abi systems that we find in Ringvent MAGs was RM Type III or RM subtype IIG, in combination with AbiE (Fig. 4). Type III RM systems cleave foreign DNA through long-range DNA interactions (enabling recognition of methylation patterns spanning two distant recognition sequences), while critically operating with substantially lower ATPase activity (approximately 1000-fold lower) than Type I RM systems [70]. The reduced energetic demand of Type III RM systems, together with their capacity to mediate precise and rapid gene regulation via long-range DNA interactions, may be an advantage in the hydrothermally active Guaymas subsurface, enabling effective prevention of phage infection while minimizing metabolic costs under increasing temperature and energy limitation. Similarly, subtype IIG RM systems, which co-occur with Type III RM systems in many Guaymas MAGs, differ from the commonly found Type I and Type II RM systems in that subtype IIG RM encodes restriction endonuclease and methyltransferase functions within a single, uninterrupted polypeptide rather than as separate subunits [71].

The most prevalent Abi system in our MAG dataset was AbiE, a two-component TA mechanism in which the antitoxin is rapidly degraded upon phage infection, allowing the toxin to disrupt essential cellular processes, such as DNA replication [10, 11]. Among the Abi systems we detected, AbiE facilitates the maintenance of plasmids and other mobile genetic elements, and provides phage resistance [10]. Other Abi systems present, although occurring less frequently than AbiE, include effectors that degrade viral tRNAs (Septu), induce host dormancy or cell death via AMP degradation (SoFic), or encode proteins and domains (e.g. WYL domains in BREX) that recognize nucleotide derivatives produced during viral infection and block phage DNA replication (Fig. 4 and Table S7). Together, these findings highlight the diversity of RM and Abi systems across broad temperatures among Guaymas subsurface prokaryotes, underscoring the central role they likely play in controlling phage propagation.

Because several of these defense systems—particularly Abi and CRISPR–Cas systems—can also target host DNA to control phage replication, we hypothesize that they may also influence host population dynamics and cell fate decisions independently of viral infections. Defense mechanisms that induce host dormancy or programmed cell death in response to viral infection may constitute part of a broader survival toolkit for subsurface microorganisms. This additional role has been discussed previously regarding microbial life and ecological dynamics in the energy-limited deep subsurface [72–74]. Beyond viral triggers, environmental cues such as energy limitation or active hydrothermalism may activate these pathways, maintaining cell integrity by enabling controlled dormancy, as supported by the expression of defense and sporulation-related genes in the Guaymas subsurface transcriptome [18] (details in Supplementary Materials).

### Horizontal gene transfer

Finding evidence for horizontal gene transfer (HGT) among pAgo genes prompted us to consider whether different classes of defense systems are affected by HGT to different degrees. RM systems are thought to be evolutionarily linked, often evolving through structural rearrangement of domains; their distribution across prokaryotic genomes is highly irregular, indicating frequent HGT, rapid gain, and loss [75]. Different RM system types (I, II, and III) are believed to have evolved from smaller, independent DNA methyltransferases, with some systems bridging the gap between Type I (hetero-oligomeric) and Type II (smaller, often single-peptide) configurations [76]. Compared to irregularly distributed RM systems that bear the imprint of HGT, pAgo genes may be less affected, although this inference remains tentative. Long and short pAgo phylogenies for (mostly) bacteria, and a smaller selection of archaea and eukaryotes show that bacterial, archaeal, and eukaryotic branches are clearly separated (Fig. S2 in [77]). An overview phylogeny for pAgos [78] also shows distinct archaeal branches scattered among much more numerous bacterial branches. Thus, new genomic data will require updated phylogenies to investigate “misplaced” lineages indicative of HGT.

### pAgo protein diversity and function

Ago proteins constitute a conserved family widely distributed across all domains of life [79]. pAgos have been implicated in defense against phage infection; however, unlike their eukaryotic counterparts (eAgos), the functions of pAgos remain poorly understood, despite their widespread occurrence in bacteria and archaea [80, 81]. pAgos have been identified through bioinformatic mining (e.g. Asgardarchaeota; [12, 46]), or in cultured bacteria and archaea from marine and hot spring environments, such as *Pyrococcus furiosus* [81], *Methanocaldococcus jannaschii* [82], *Synechococcus elongatus* [83], *Marinitoga piezophila*, *Thermotoga profunda*, and *Rhodobacter sphaeroides* [84, 85]. pAgos from thermophilic bacteria and archaea (e.g. from *T. thermophilus, P. furiosus, M. jannaschii*) have higher thermostability and higher temperature optima of activity than mesophilic pAgos (see Table S1 in [86]); pAgos from such groups could have a selective advantage in the hot Guaymas Basin subsurface.

Until recently, only a limited number of pAgos were well characterized and structurally resolved [87, 88]. Current evidence indicates pAgos can suppress phage propagation by acting as NADases that deplete the cellular NAD^+^ pool required for phage replication, or as nucleases that cleave invading phage DNA [79, 89, 90]. This information derives primarily from structural studies of pAgo proteins from thermophilic bacteria (e.g. *Thermus thermophilus*) and thermophilic or hyperthermophilic archaea (e.g. *M. jannaschii*, *P. furiosus*) [91]. pAgos exhibit diverse domain architectures [90], and phylogenetic analyses classify them into three major groups: long-A, long-B, and short pAgos [78]. Phylogenetic analysis of our nine Guaymas pAgos reveals long-A and long-B pAgo protein variants and the presence of PIWI, Alba and Toll/interleukin-2 receptor (TIR)-like domains, characteristic of long pAgo variants [88]. Three-dimensional structure predictions [49, 50] confirm that all nine sequences are TIR and PIWI domain-containing pAgo proteins (Supplementary pdf files). These domains are well known for their roles in innate immunity, including nucleic acid recognition and binding, and immune signaling [89]. Notably, long-A and long-B pAgos share structural and functional similarities with eAgos [77], and emerging evidence suggests some long pAgos can mediate RNA cleavage and gene silencing when heterologously expressed in human cells [12]. This finding possibly extends the relevance of pAgos beyond prokaryotic antiviral defense and eukaryotic immunity [46], to the evolutionary origins of eukaryotic RNA interference and silencing pathways [12].

### Controls on defense gene systems

Viruses are common in hydrothermal fluids and shallow sediments associated with deep-sea hydrothermal systems [92–94] and in the sedimentary deep subsurface [95, 96], and they exceed prokaryotic abundance in deep subseafloor sediments by approximately an order of magnitude [97]. The abundant viral repertoire in surficial hydrothermal habitats [98] has been described in the context of specific bacterial and archaeal hosts [99, 100]. CRISPR spacer matches to the virome of diffuse fluids from Juan de Fuca vents in the northeast Pacific indicate viruses infect hosts from diverse taxonomic groups, not just a few well-studied microorganisms [101]. Viral populations in bottom waters, fluids, and near-surface sediments at different vent sites were dominated by abundant unclassified viruses, tailed phages that are common to global hydrothermal vents, and single-stranded DNA viruses, including Microviridae and small eukaryotic viruses [102]. Protein-sharing network analysis demonstrated that hydrothermal vent viruses are highly endemic to specific vent sites and habitat types and belong to novel genus-level viral clusters; only 11% of vOTUs could be linked to specific microbial hosts (Gammaproteobacteria and Campylobacterota) [102]. Our CRISPR spacer analysis indicates a viral subsurface community consisting primarily of previously unidentified viruses not yet adequately catalogued in available databases. Thus, the microbial defense systems discussed above are likely to interact with a viral subsurface community of complex and novel composition.

Any hypotheses on what controls the environmental defense gene repertoire in the deep hydrothermal subsurface must be qualified by the fact that this study is based on microbial counts and microbial metagenomes, not viral counts or metagenomes. Yet, cell counts and virus-to-prokaryote ratios (VPRs) from other subsurface sites provide a sense of scale. For cold open-ocean sediments in Porcupine Bay (Atlantic Ocean), VPRs range from a factor of 25 in surficial sediments to a factor of 5 downcore, while cell counts decline by half an order of magnitude [97]. For coastal, highly reducing sediments of Saanich Inlet (British Columbia, Canada), VPRs remain around 3 for the top 100 mbsf, while cell counts decrease by one order of magnitude [103]. In contrast to these profiles, microbial cell numbers for hydrothermally heated Guaymas Basin sediments at Sites U1547 and U1548 decline rapidly, five orders of magnitude within ca. seventy meters (Table S3). If VPRs in Guaymas Basin remain within a similar range as observed elsewhere (factor 3 to 30), subsurface virus density—and with it, microbial defense gene repertoire—should decline rapidly, in parallel to cell counts. Against a null hypothesis that the defense gene repertoire does not decline with depth, our results show that the defense gene repertoire declines downcore, as temperature increases and phylogenetic host range narrows toward specific archaea, with the latter as the decisive factor. Similarly, although CRISPR loci are often enriched in thermophilic and hyperthermophilic prokaryotes [104], our phylogeny-aware analyses indicate CRISPR–Cas occurrence in Guaymas Basin is better explained by host community structure than by temperature as an independent predictor. To extrapolate these insights toward a generalized working hypothesis, the phylogenetic composition of deep subsurface microbial communities—reflecting environmental selection and physiological adaptation—may have predictive potential for their defense gene repertoire.

## Supplementary Material

Supplementary_material_ycag200

## Data Availability

Previously obtained metagenomic sequences for samples from Holes U1547B and 1548B were re-analyzed (Bioproject accession number PRJNA909197: accession numbers SRR22580796–SRR22580804, SRR23614663–SRR23614665, SRR23614673–SRR23614675), in combination with new metagenomic sequence data from Holes U1548C and U1547D (Bioproject accession number PRJNA1422517; accession numbers SRR37204548–SRR37204551).

## References

[1] Mushegian AR . Are there 10^31^ virus particles on Earth, or more, or fewer? *J Bacteriol* 2020;202:e00052–20. 10.1128/JB.00052-20 32071093 PMC7148134

[2] Suttle CA . Marine viruses—major players in the global ecosystem. *Nat Rev Microbiol* 2007;5:801–12. 10.1038/nrmicro1750 17853907

[3] Beavogui A, Lacroix A, Wiart N. et al. The defensome of complex bacterial communities. *Nat Commun* 2024;15:2146. 10.1038/s41467-024-46489-0 38459056 PMC10924106

[4] Tesson F, Huiting E, Wei L. et al. Exploring the diversity of anti-defense systems across prokaryotes, phages and mobile genetic elements. *Nucleic Acids Res* 2025;53:gkae1171. 10.1093/nar/gkae1171 PMC11724313 39657785

[5] Millman A, Melamed S, Leavitt A. et al. An expanded arsenal of immune systems that protect bacteria from phages. *Cell Host Microbe* 2022;30:1556–1569.e5. 10.1016/j.chom.2022.09.017 36302390

[6] Shaw LP, Rocha EPC, MacLean RC. Restriction-modification systems have shaped the evolution and distribution of plasmids across bacteria. *Nucleic Acids Res* 2023;51:6806–18. 10.1093/nar/gkad452 37254807 PMC10359461

[7] Oliveira PH, Touchon M, Rocha EP. The interplay of restriction-modification systems with mobile genetic elements and their prokaryotic hosts. *Nucleic Acids Res* 2014;42:10618–31. 10.1093/nar/gku734 25120263 PMC4176335

[8] Aframian N, Eldar A. Abortive infection antiphage defense systems: separating mechanism and phenotype. *Trends Microbiol* 2023;31:1003–12. 10.1016/j.tim.2023.05.002 37268559

[9] Bethke JH, Kimbrel J, Jiao Y. et al. Toxin-antitoxin systems reflect community interactions through horizontal gene transfer. *Mol Biol Evol* 2024;41:msae206. 10.1093/molbev/msae206 PMC11523183 39404847

[10] Dy RL, Przybilski R, Semeijn K. et al. A widespread bacteriophage abortive infection system functions through a Type IV toxin-antitoxin mechanism. *Nucleic Acids Res* 2014;42:4590–605. 10.1093/nar/gkt1419 24465005 PMC3985639

[11] Hampton HG, Jackson SA, Fagerlund RD. et al. AbiEi binds cooperatively to the type IV abiE toxin-antitoxin operator via a positively-charged surface and causes DNA bending and negative autoregulation. *J Mol Biol* 2018;430:1141–56. 10.1016/j.jmb.2018.02.022 29518409

[12] Bastiaanssen C, Bobadilla Ugarte P, Kim K. et al. RNA-guided RNA silencing by an Asgard archaeal Argonaute. *Nat Commun* 2024;15:5499. 10.1038/s41467-024-49452-1 38951509 PMC11217426

[13] Wang X, Yu D, Yu J. et al. Toll/interleukin-1 receptor (TIR) domain-containing proteins have NAD-RNA decapping activity. *Nat Commun* 2024;15:2261. 10.1038/s41467-024-46499-y 38480720 PMC10937652

[14] Koonin EV, Makarova KS. Origins and evolution of CRISPR-Cas systems. *Philos Trans R Soc Lond B Biol Sci* 2019;374:20180087. 10.1098/rstb.2018.0087 30905284 PMC6452270

[15] van Beljouw SPB, Sanders J, Rodríguez-Molina A. et al. RNA-targeting CRISPR-Cas systems. *Nat Rev Microbiol* 2023;21:21–34. 10.1038/s41579-022-00793-y 36171275

[16] Einsele G, Gieskes JM, Curray J. et al. Intrusion of basaltic sills into highly porous sediments, and resulting hydrothermal activity. *Nature* 1980;283:441–5. 10.1038/283441a0

[17] Teske A, Lizarralde D, Höfig TW. et al. Expedition 385 summary. In Teske, A., Lizarralde, D., and Höfig, T.W. (eds), Guaymas Basin Tectonics and Biosphere. Proceedings of the International Ocean Discovery Program, Vol. 385. College Station, TX: International Ocean Discovery Program, 2021, 10.14379/iodp.proc.385.101.2021

[18] Mara P, Zhou YL, Teske A. et al. Microbial gene expression in Guaymas Basin subsurface sediments responds to hydrothermal stress and energy limitation. *ISME J* 2023;17:1907–19. 10.1038/s41396-023-01492-z 37658181 PMC10579382

[19] Mara P, Geller-McGrath D, Edgcomb V. et al. Metagenomic profiles of archaea and bacteria within thermal and geochemical gradients of the Guaymas Basin deep subsurface. *Nat Commun* 2023;14:7768. 10.1038/s41467-023-43296-x 38012208 PMC10681998

[20] Mara P, Beaudoin D, Aiello I. et al. Deep subseafloor sediments in Guaymas Basin harbor cosmopolitan microbiota and traces of hydrothermal populations. *Commun Earth Environ* 2024;5:505. 10.1038/s43247-024-01662-7

[21] Ramírez GA, Mara P, Beaudoin D. et al. Data Report: The DNA Event Horizon in the Guaymas Basin Subsurface Biosphere: Technical Advances and Redefined Limits in Bulk Extractions of Nucleic Acids from Deep Marine Sediments, IODP Expedition 385. In: Teske A., Lizarralde D., Höfig T.W. (eds.), Guaymas Basin Tectonics and Biosphere. Proceedings of the International Ocean Discovery Program, Vol. 385. College Station, TX: International Ocean Discovery Program, 2024.

[22] Knoke M, Dittmar T, Günther J. et al. Hydrothermal mobilization and molecular transformation of dissolved organic matter from deep subsurface sediments. *Geochim Cosmochim Acta* 2025;402:359–71. 10.1016/j.gca.2025.06.005

[23] Nagakura T, Schubert F, Wagner D. et al. 385 shipboard scientific party. Biological sulfate reduction in deep subseafloor sediment of Guaymas Basin. *Front Microbiol* 2022;13:845250. 10.3389/fmicb.2022.845250 35308366 PMC8927301

[24] Torres ME, Kim J-H. Data Report: Concentration and Carbon Isotopic Composition in Pore Fluids from IODP Expedition 385. In Teske, A., Lizarralde, D., and Höfig, T.W. (eds), Guaymas Basin Tectonics and Biosphere. Proceedings of the International Ocean Discovery Program, Vol. 385. College Station, TX, USA: International Ocean Discovery Program, 2022, 10.14379/iodp.proc.385.201.2022

[25] Teske A, Lizarralde D, Höfig TW. et al. Sites U1547 and U1548*.* In Teske, A., Lizarralde, D., and Höfig, T.W. (eds), Guaymas Basin Tectonics and Biosphere. Proceedings of the International Ocean Discovery Program, Vol. 385. College Station, TX, USA: International Ocean Discovery Program, 2021, 10.14379/iodp.proc.385.105.2021

[26] Williams JRA, Biddle JF. Viruses in marine sediments: a review of their effect on biogeochemistry and microbial interactions. *Appl Environ Microbiol* 2026;92:e0027525. 10.1128/aem.00275-25 41784386 PMC13101502

[27] Uritskiy GV, DiRuggiero J, Taylor J. MetaWRAP-a flexible pipeline for genome-resolved metagenomic data analysis. *Microbiome* 2018;6:158. 10.1186/s40168-018-0541-1 30219103 PMC6138922

[28] Li D, Luo R, Liu CM. et al. MEGAHIT v1.0: a fast and scalable metagenome assembler driven by advanced methodologies and community practices. *Methods* 2016;102:3–11. 10.1016/j.ymeth.2016.02.020 27012178

[29] Chklovski A, Parks DH, Woodcroft BJ. et al. CheckM2: a rapid, scalable and accurate tool for assessing microbial genome quality using machine learning. *Nat Methods* 2023;20:1203–12. 10.1038/s41592-023-01940-w 37500759

[30] Chaumeil PA, Mussig AJ, Hugenholtz P. et al. GTDB-Tk v2: memory friendly classification with the genome taxonomy database. *Bioinformatics* 2022;38:5315–6. 10.1093/bioinformatics/btac672 36218463 PMC9710552

[31] Aroney STN, Newell RJP, Nissen JN. et al. CoverM: read alignment statistics for metagenomics. *Bioinformatics* 2025;41:btaf147. 10.1093/bioinformatics/btaf147 PMC11993303 40193404

[32] Seemann T . Prokka: rapid prokaryotic genome annotation. *Bioinformatics* 2014;30:2068–9. 10.1093/bioinformatics/btu153 24642063

[33] Shaffer M, Borton MA, McGivern BB. et al. DRAM for distilling microbial metabolism to automate the curation of microbiome function. *Nucleic Acids Res* 2020;48:8883–900. 10.1093/nar/gkaa621 32766782 PMC7498326

[34] Zhou Z, Tran PQ, Breister AM. et al. METABOLIC: high-throughput profiling of microbial genomes for functional traits, metabolism, biogeochemistry, and community-scale functional networks. *Microbiome* 2022;10:33. 10.1186/s40168-021-01213-8 35172890 PMC8851854

[35] Tesson F, Hervé A, Mordret E. et al. Systematic and quantitative view of the antiviral arsenal of prokaryotes. *Nat Commun* 2022;13:2561. 10.1038/s41467-022-30269-9 35538097 PMC9090908

[36] Na SI, Kim YO, Yoon SH. et al. UBCG: up-to-date bacterial core gene set and pipeline for phylogenomic tree reconstruction. *J Microbiol* 2018;56:280–5. 10.1007/s12275-018-8014-6 29492869

[37] Kalyaanamoorthy S, Minh B, Wong TKF. et al. ModelFinder: fast model selection for accurate phylogenetic estimates. *Nat Methods* 2017;14:587–9. 10.1038/nmeth.4285 28481363 PMC5453245

[38] Hoang DT, Chernomor O, von Haeseler A. et al. UFBoot2: improving the ultrafast bootstrap approximation. *Mol Biol Evol* 2018;35:518–22. 10.1093/molbev/msx281 29077904 PMC5850222

[39] Schliep KP . Phangorn: phylogenetic analysis in R. *Bioinformatics* 2011;27:592–3. 10.1093/bioinformatics/btq706 21169378 PMC3035803

[40] Orme D . The Caper Package: Comparative Analysis of Phylogenetics and Evolution in R, 2018, Available from: https://cran.r-project.org/web/packages/caper/vignettes/caper.pdf.

[41] Pagel M . Inferring the historical patterns of biological evolution. *Nature* 1999;401:877–84. 10.1038/44766 10553904

[42] Ho LT, Ané C. A linear-time algorithm for Gaussian and non-Gaussian trait evolution models. *Syst Biol* 2014;63:397–408. 10.1093/sysbio/syu005 24500037

[43] Wood DE, Lu J, Langmead B. Improved metagenomic analysis with Kraken 2. *Genome Biol* 2019;20:257. 10.1186/s13059-019-1891-0 31779668 PMC6883579

[44] Lu J, Breitwieser FP, Thielen P. et al. Bracken: estimating species abundance in metagenomics data. *PeerJ Comput Sci* 2017;3:e104. 10.7717/peerj-cs.104 PMC12016282 40271438

[45] Altschul SF, Gish W, Miller W. et al. Basic local alignment search tool. *J Mol Biol* 1990;215:403–10. 10.1016/S0022-2836(05)80360-2 2231712

[46] Leão P, Little ME, Appler KE. et al. Asgard archaea defense systems and their roles in the origin of eukaryotic immunity. *Nat Commun* 2024;15:6386. 10.1038/s41467-024-50195-2 39085212 PMC11291487

[47] Gilbert D . *SeqPup v.0.6f*: A Biosequence Editor and Analysis Application Available through Anonymous. 1996. https://iubio.bio.indiana.edu/molbio/seqpup/

[48] Swofford DL . PAUP. Phylogenetic Analysis Using Parsimony (and Other Methods). Version 4. Sunderland, Massachusetts: Sinauer Associates, 2022.

[49] Kelley LA, Mezulis S, Yates CM. et al. The Phyre2 web portal for protein modeling, prediction and analysis. *Nat Protoc* 2015;10:845–58. 10.1038/nprot.2015.053 25950237 PMC5298202

[50] Powell HR, Islam SA, David A. et al. Phyre2.2: a community resource for template-based protein structure prediction. *J Mol Biol* 2025;437:168960. 10.1016/j.jmb.2025.168960 40133783 PMC7617537

[51] Teske A, McKay LJ, Ravelo AC. et al. Characteristics and evolution of sill-driven off-axis hydrothermalism in Guaymas Basin - the Ringvent site. *Sci Rep* 2019;9:13847. 10.1038/s41598-019-50200-5 31554864 PMC6761151

[52] Neumann F, Negrete-Aranda R, Harris RN. et al. Heat flow and thermal regime in the Guaymas Basin, Gulf of California: estimates of conductive and advective heat transport. *Basin Res* 2023;35:1308–28. 10.1111/bre.12755

[53] Wang L, Zhang L. The arms race between bacteria CBASS and bacteriophages. *Front Immunol* 2023;14:1224341. 10.3389/fimmu.2023.1224341 37575224 PMC10419184

[54] Gu Y, Li H, Deep A. et al. Bacterial Shedu immune nucleases share a common enzymatic core regulated by diverse sensor domains. *Mol Cell* 2025;85:523–536.e6. 10.1016/j.molcel.2024.12.004 39742666 PMC11805627

[55] Makarova KS, Shmakov SA, Wolf YI. et al. An updated evolutionary classification of CRISPR-Cas systems including rare variants. *Nat Microbiol* 2025;10:3346–61. 10.1038/s41564-025-02180-8 41198952 PMC12669027

[56] Molina R, Stella S, Feng M. et al. Structure of Csx1-cOA4 complex reveals the basis of RNA decay in type III-B CRISPR-Cas. *Nat Commun* 2019;10:4302. 10.1038/s41467-019-12244-z 31541109 PMC6754442

[57] Sheppard NF, Glover CV 3rd, Terns RM. et al. The CRISPR-associated Csx1 protein of *Pyrococcus furiosus* is an adenosine-specific endoribonuclease. *RNA.* 2016;22:216–24. 10.1261/rna.039842.113 26647461 PMC4712672

[58] Anantharaman V, Makarova KS, Burroughs AM. et al. Comprehensive analysis of the HEPN superfamily: identification of novel roles in intra-genomic conflicts, defense, pathogenesis and RNA processing. *Biol Direct* 2013;8:15. 10.1186/1745-6150-8-15 23768067 PMC3710099

[59] Sheik CS, Reese BK, Twing KI. et al. Identification and removal of contaminant sequences from ribosomal gene databases: Lessons from the Census of Deep Life. *Front Microbiol* 2018;9:840. 10.3389/fmicb.2018.00840 29780369 PMC5945997

[60] Song JJ, Joshua-Tor L. Argonaute and RNA--getting into the groove. *Curr Opin Struct Biol* 2006;16:5–11. 10.1016/j.sbi.2006.01.010 16434185

[61] Jagadeesh J, Vembar SS. Evolution of sequence, structural and functional diversity of the ubiquitous DNA/RNA-binding Alba domain. *Sci Rep* 2024;14:30363. 10.1038/s41598-024-79937-4 39638848 PMC11621453

[62] Mahmoudabadi G, Milo R, Phillips R. Energetic cost of building a virus. *Proc Natl Acad Sci U S A* 2017;114:4–E4333. 10.1073/pnas.1701670114 PMC5465929 28512219

[63] Thomas E, Anderson RE, Li V. et al. Diverse viruses in deep-sea hydrothermal vent fluids have restricted dispersal across ocean basins. *mSystems* 2021;6:e0006821. 10.1128/mSystems.00068-21 34156293 PMC8269205

[64] Hoehler TM, Jørgensen BB. Microbial life under extreme energy limitation. *Nat Rev Microbiol* 2013;11:83–94. 10.1038/nrmicro2939 23321532

[65] Burstein D, Harrington LB, Strutt SC. et al. New CRISPR-Cas systems from uncultivated microbes. *Nature* 2017;542:237–41. 10.1038/nature21059 28005056 PMC5300952

[66] Salgado O, Guajardo-Leiva S, Moya-Beltrán A. et al. Global phylogenomic novelty of the Cas1 gene from hot spring microbial communities. *Front Microbiol* 2022;13:1069452. 10.3389/fmicb.2022.1069452 36532491 PMC9755687

[67] Ostyn E, Augagneur Y, Pinel-Marie ML. Insight into the environmental cues modulating the expression of bacterial toxin-antitoxin systems. *FEMS Microbiol Rev* 2025;49:fuaf007. 10.1093/femsre/fuaf007 40052347 PMC11951105

[68] Arias CF, Acosta FJ, Bertocchini F. et al. The coordination of anti-phage immunity mechanisms in bacterial cells. *Nat Commun* 2022;13:7412. 10.1038/s41467-022-35203-7 36456580 PMC9715693

[69] Pingoud A, Jeltsch A. Structure and function of type II restriction endonucleases. *Nucleic Acids Res* 2001;29:3705–27. 10.1093/nar/29.18.3705 11557805 PMC55916

[70] van Aelst K, Tóth J, Ramanathan SP. et al. Type III restriction enzymes cleave DNA by long-range interaction between sites in both head-to-head and tail-to-tail inverted repeat. *Proc Natl Acad Sci U S A* 2010;107:9123–8. 10.1073/pnas.1001637107 20435912 PMC2889075

[71] Shen BW, Xu D, Chan SH. et al. Characterization and crystal structure of the type IIG restriction endonuclease RM.BpuSI. *Nucleic Acids Res* 2011;39:8223–36. 10.1093/nar/gkr543 21724614 PMC3185434

[72] Jørgensen BB . Shrinking majority of the deep biosphere. *Proc Natl Acad Sci U S A* 2012;109:15976–7. 10.1073/pnas.1213639109 23012471 PMC3479554

[73] Orcutt BN, Sylvan JB, Knab NJ. et al. Microbial ecology of the dark ocean above, at, and below the seafloor. *Microbiol Mol Biol Rev* 2011;75:361–422. 10.1128/MMBR.00039-10 21646433 PMC3122624

[74] Cario A, Oliver GC, Rogers KL. Exploring the deep marine biosphere: challenges, innovations, and opportunities. *Front Earth Sci* 2019;7:225. 10.3389/feart.2019.00225

[75] Fullmer MS, Ouellette M, Louyakis AS. et al. The patchy distribution of restriction^−^modification system genes and the conservation of orphan Methyltransferases in Halobacteria. *Genes.* 2019;10:233. 10.3390/genes10030233 30893937 PMC6471742

[76] Bower EKM, Cooper LP, Roberts GA. et al. A model for the evolution of prokaryotic DNA restriction-modification systems based upon the structural malleability of type I restriction-modification enzymes. *Nucleic Acids Res* 2018;46:9067–80. 10.1093/nar/gky760 30165537 PMC6158711

[77] Swarts DC, Makarova K, Wang Y. et al. The evolutionary journey of Argonaute proteins. *Nat Struct Mol Biol* 2014;21:743–53. 10.1038/nsmb.2879 25192263 PMC4691850

[78] Ryazansky S, Kulbachinskiy A, Aravin AA. The expanded universe of prokaryotic Argonaute proteins. *mBio* 2018;9:e01935–18. 10.1128/mBio.01935-18 30563906 PMC6299218

[79] Zhang W, Jiang Y, Li Y. et al. Filament assembly induced by the recognition of target DNA activates the prokaryotic Argonaute SPARDA system. *Nat Commun* 2026;17:115. 10.1038/s41467-025-68195-1 41495039 PMC12774872

[80] Chen Y, Zeng Z, She Q. et al. The abortive infection functions of CRISPR-Cas and Argonaute. *Trends Microbiol* 2023;31:405–18. 10.1016/j.tim.2022.11.005 36463018

[81] Swarts DC, Hegge JW, Hinojo I. et al. Argonaute of the archaeon *Pyrococcus furiosus* is a DNA-guided nuclease that targets cognate DNA. *Nucleic Acids Res* 2015;43:5120–9. 10.1093/nar/gkv415 25925567 PMC4446448

[82] Zander A, Willkomm S, Ofer S. et al. Guide-independent DNA cleavage by archaeal Argonaute from *Methanocaldococcus jannaschii*. *Nat Microbiol* 2017;2:17034. 10.1038/nmicrobiol.2017.34 28319081 PMC7616673

[83] Olina A, Kuzmenko A, Ninova M. et al. Genome-wide DNA sampling by Ago nuclease from the cyanobacterium *Synechococcus elongatus*. *RNA Biol* 2020;17:677–88. 10.1080/15476286.2020.1724716 32013676 PMC7237159

[84] Kaya E, Doxzen KW, Knoll KR. et al. A bacterial Argonaute with noncanonical guide RNA specificity. *Proc Natl Acad Sci U S A* 2016;113:4057–62. 10.1073/pnas.1524385113 27035975 PMC4839417

[85] Miyoshi T, Ito K, Murakami R. et al. Structural basis for the recognition of guide RNA and target DNA heteroduplex by Argonaute. *Nat Commun* 2016;7:11846. 10.1038/ncomms11846 27325485 PMC4919518

[86] Pantaleev V, Kropocheva E, Esyunina D. et al. Strong temperature effects on the fidelity of target DNA recognition by a thermophilic pAgo nuclease. *Biochemie* 2023;209:142–9. 10.1016/j.biochi.2023.02.007 36804511

[87] Moya-Beltrán A, Rojas-Villalobos C, Díaz M. et al. Nucleotide second messenger-based signaling in extreme acidophiles of the *Acidithiobacillus* species complex: partition between the core and variable gene complements. *Front Microbiol* 2019;10:381. 10.3389/fmicb.2019.00381 30899248 PMC6416229

[88] Wang X, Li X, Yu G. et al. Structural insights into mechanisms of Argonaute protein-associated NADase activation in bacterial immunity. *Cell Res* 2023;33:699–711. 10.1038/s41422-023-00839-7 37311833 PMC10474274

[89] Swarts DC, Jore MM, Westra ER. et al. DNA-guided DNA interference by a prokaryotic Argonaute. *Nature* 2014;507:258–61. 10.1038/nature12971 24531762 PMC4697943

[90] Kottur J, Malik R, Aggarwal AK. Nucleic acid mediated activation of a short prokaryotic Argonaute immune system. *Nat Commun* 2024;15:4852. 10.1038/s41467-024-49271-4 38844755 PMC11156904

[91] Graver BA, Chakravarty N, Solomon KV. Prokaryotic Argonautes for in vivo biotechnology and molecular diagnostics. *Trends Biotechnol* 2024;42:61–73. 10.1016/j.tibtech.2023.06.010 37451948

[92] Langwig MV, Koester F, Martin C. et al. Endemism shapes viral ecology and evolution in globally distributed hydrothermal vent ecosystems. *Nat Commun* 2025;16:4076. 10.1038/s41467-025-59154-x 40307239 PMC12043954

[93] Wirth J, Young M. Viruses in subsurface environments. *Annu Rev Virol* 2022;9:99–119. 10.1146/annurev-virology-093020-015957 36173700

[94] Han Y, Gao C, Liang Y. et al. Diversity and ecological roles of deep-sea viruses. *Ocean-Land-Atmos Res* 2024;3:0067. 10.34133/olar.0067

[95] Engelhardt T, Kallmeyer J, Cypionka H. et al. High virus-to-cell ratios indicate ongoing production of viruses in deep subsurface sediments. *ISME J* 2014;8:1503–9. 10.1038/ismej.2013.245 24430483 PMC4069387

[96] Cai L, Jørgensen BB, Suttle CA. et al. Active and diverse viruses persist in the deep sub-seafloor sediments over thousands of years. *ISME J* 2019;13:1857–64. 10.1038/s41396-019-0397-9 30877284 PMC6776017

[97] Middelboe M, Glud RN, Filippini M. Viral abundance and activity in the deep sub-seafloor biosphere. *Aquat Microb Ecol* 2011;63:1–8. 10.3354/ame01485

[98] Ortmann AC, Suttle CA. High abundances of viruses in a deep-sea hydrothermal vent system indicates viral mediated microbial mortality. *Deep Sea Res Part* 2005;52:1515–27. 10.1016/j.dsr.2005.04.002

[99] Lossouarn J, Dupont S, Gorlas A. et al. An abyssal mobilome: viruses, plasmids and vesicles from deep-sea hydrothermal vents. *Res Microbiol* 2015;166:742–52. 10.1016/j.resmic.2015.04.001 25911507

[100] Takaki Y, Shimamura S, Nakagawa S. et al. Bacterial lifestyle in a deep-sea hydrothermal vent chimney revealed by the genome sequence of the thermophilic bacterium *Deferribacter desulfuricans* SSM1. *DNA Res* 2010;17:123–37. 10.1093/dnares/dsq005 20189949 PMC2885270

[101] Anderson RE, Brazelton WJ, Baross JA. Using CRISPRs as a metagenomic tool to identify microbial hosts of a diffuse flow hydrothermal vent viral assemblage. *FEMS Microbiol Ecol* 2011;77:120–33. 10.1111/j.1574-6941.2011.01090.x 21410492

[102] Cheng R, Li X, Jiang L. et al. Virus diversity and interactions with hosts in deep-sea hydrothermal vents. *Microbiome* 2022;10:235. 10.1186/s40168-022-01441-6 36566239 PMC9789665

[103] Bird DF, Juniper SK, Ricciardi-Rigault M. et al. Subsurface viruses and bacteria in Holocene/Late Pleistocene sediments of Saanich Inlet, BC: ODP Holes 1033B and 1034B, Leg 169S. *Mar Geol* 2001;174:227–39. 10.1016/S0025-3227(00)00152-3

[104] Anderson RE, Brazelton WJ, Baross JA. Is the genetic landscape of the deep subsurface biosphere affected by viruses? *Front Microbiol* 2011;2:219. 10.3389/fmicb.2011.00219 22084639 PMC3211056

