# Supplementary material for: From CRISPR–Cas to Argonautes: Defense systems of bacteria and archaea in the hydrothermal deep subsurface
Source: ISME Commun. 2026 Jul 10;6(1):ycag200. doi: 10.1093/ismeco/ycag200 (PMC13477220; doi:10.1093/ismeco/ycag200)
Supplement: Supplementary file 1 [file supplementary_material_ycag200.zip › Supplementary Materials revised July 21.docx]

**From CRISPR-Cas to Argonautes: Defense systems of bacteria and archaea in the hydrothermal deep subsurface**

**Supplementary Materials**

**Supplementary Methods, p. 2-5**

**Supplementary Results, p. 6**

**Supplementary Tables, p. 7- 11**

**Supplementary Figures, p. 12-14**

**Supplementary Discussion, p. 15-17**

**Supplementary References, p. 18-19**

***Supplementary Methods***

***Sampling site overview*.** IODP Expedition 385 drilled eight sites (U1545-U1552) in Guaymas Basin, each representing different levels of hydrothermal influence [1]; thus, the Guaymas Basin sample set is the only one that tracked hydrothermal subsurface gradients along well-defined depth and temperature gradients by deep ocean drilling (IODP Expedition 385). Paired sites U1547 and U1548, located ~27 km northwest of the axial valley, targeted high-temperature sediments close to a shallow, recently emplaced intrusion of stacked sills (the Ringvent area; ca. 5,000 to 10,000 years old) that still creates steep thermal gradients and drives active hydrothermal circulation [2-4]. The near-surface edge of this bowl-shaped sill complex (Figure 1A) channels hydrothermal fluids upward toward the elevated, ring-shaped hydrothermal crest of the system. Hydrothermal activity at Ringvent is evident from silica-dominated precipitates, warm fluid flows, sulfur-oxidizing microbial mats, and chemosynthetic clams and tubeworms [3]. Ringvent Sites U1547 and U1548 not only display the steepest geothermal gradients among the eight IODP-drilled sites [1, 4], but they also show the most pronounced downcore decline in DNA yield and microbial cell abundance (Table S1) [5]. Genomic sequencing surveys indicate that Ringvent sediments sustain active hydrothermal microbial assemblages, including hyperthermophilic methanogens [6, 7].

***DNA extraction from sill rock***. For veined and crushed rock samples (Table S2), the DNA extraction was performed with MP Biomedical FastDNA™ SPIN Kit with additions and modifications. In brief, approx. 20 ml crushed rock is placed in sterile 50 ml Falcon tube, filled up with 30 ml DNA and RNAse-free water, and vortexed 5 min vigorously to dislodge cells. The resulting grey sludge of fine-grained particles that do not sink is filtered through a 0.1 µm VCTP filter (Millipore) using suction (Rocker 300 pump, moderate vacuum of 70 kpa). Filtering the fine-grained suspension continues for ca. 2 h, as the rocky dust is slowly covering and clogging the filter. Once filtration is complete, the filter (covered with grey rock dust) is taken up with a forceps, rolled up and placed in 2 ml Eppendorf tube, frozen at -80°C until further processing. Each filter is cut into small pieces with sterile scissors or razor blades, and extracted following the modified MP Biomedical extraction protocol [5]. Briefly, the filter pieces are added to an MP extraction vial with beads, and extraction buffers are added according to the protocol (122 µl MT buffer and approx. 900 µl phosphate buffer). A vortexing step of ca. 5 minutes dislodges the rock dust from the filter, resulting in a grey-colored suspension. Aqueous solution and rock powder are separated by 2 x 5 minutes at 13,000 rpm; a single round is not sufficient since beads and rock powder will settle only to some extent, which limits the amount of solution that can be recovered. After collecting the aqueous buffer solution (now containing cells and DNA) as much as possible, the sample is extracted with another buffer addition (approx. 0.4 ml); both portions of aqueous extract are subsequently pooled. The two pooled extractions from rock samples yield approx. the same volume as a single extraction from mudline fluid, or from the negative control. From here on, the protocol follows published procedure [5].

**DNA processing for Library construction**. Submitted DNA was processed on a sample-per-sample basis at the University of Delaware Sequencing and Genotyping center (UDSG). For the rock DNA sample, submitted DNA was cleaned and concentrated using Genomic DNA Clean & Concentrator-10 (Zymo Research Corp.) and eluted in 11.5 ul. Concentrated DNA was used as input for sequencing library preparation using Illumina DNA library preparation kit at a 1/3rd reaction volume. 25 PCR cycles were used in the library prep. An additional SPRI bead cleanup was performed on the final sequencing library to remove shorter library fragments and adapter dimers. For sediments from site U1548C, up to 10ng or 24uL of submitted DNA (see chart below) was used for library preparation using Illumina DNA preparation kit. The on-bead PCR reaction was performed at 1/3^rd^ reaction volumes to increase tagmentation product concentration in the reaction.  20 PCR cycles were used.  An additional SPRI bead cleanup was performed on the final sequencing library to remove shorter library fragments and adapter dimers.

| **Sample** | **C(ng/uL)** | **V input(uL)** | **Mass input(ng)** |
| --- | --- | --- | --- |
| 11 (U1548C_1H3) | 10.8 | 0.93 | 10 |
| 12 (U1548C_3H6) | 0.192 | 22 | 4.224 |
| 13 (U1548C_5H4) | 0.00345 | 24 | 0.0828 |
| 14 (U1548C_6H4) | 0.00247 | 1 | 0.00247 |
| 15 (U1548C_7H4) | 7.94E-05 | 13 | 0.0010322 |
| 16 (blind prep) | 7.82E-05 | 22 | 0.0017204 |

**Metagenomic profiling before MAG assembly**. Kraken2 profiling detected substantially more phyla per sample (mean 48.8 in cool, 47.2 in warm, and 58.1 in hot samples) than MAG assembly alone (mean 12.8, 9.3, and 16.4 phyla per sample, respectively), confirming that MAGs provide only a partial window into community composition. Kraken2 profiles were dominated by Pseudomonadota (39–60%), Actinomycetota (19–24%), and Bacillota (10–22%) across all temperature bins, consistent with these phyla being well-represented in current reference databases (Supplementary Figure S1). The high proportions of Pseudomonadota, unusual for deep subsurface sediments, suggest that sequences of non-subsurface origin may have passed the automated quality control and decontamination procedures. Interestingly, the relative contribution of Bacillota was approximately twice as high in hot sediments (22.3%) compared with cool sediments (10.3%), suggesting a relative enrichment of thermophilic Firmicutes-related lineages at elevated temperatures (Figure S1). Kraken2 also detected viral sequences (Artverviricota, Uroviricota, Dividoviricota, Cossaviricota) in all samples, consistent with the high viral abundance expected in deep subseafloor environments. PERMANOVA of Kraken2-based community composition showed that neither temperature (R² = 0.024, F = 0.422, P = 0.732) nor depth (R² = 0.021, F = 0.360, P = 0.775) significantly structured community composition.

GTDB-Tk (used for MAG classification) and Kraken2 (using NCBI taxonomy) differ in phylum-level nomenclature conventions (GTDB uses prefix notation, e.g., "p__Actinomycetota" vs. NCBI "Actinomycetota"). Thus, direct phylum-level overlap was assessed by name harmonization; major lineages including Actinomycetota, Chloroflexota, Planctomycetota, Spirochaetota, and Pseudomonadota were detected by both approaches. Several lineages specific for the deep subsurface were recovered by MAG assembly (Hadarchaeota, Thermoproteota, WOR-3, Zixibacteria, Zhuqueibacterota), but were absent from Kraken2 profiles, reflecting their limited representation in NCBI taxonomy and databases. These results confirm that Kraken2 profiling and MAG-based assembly are complementary: Kraken2 provides broad community context anchored to well-characterized reference genomes, while MAG assembly preferentially recovers novel deep-subsurface lineages inadequately represented in current databases.

Pairwise Wilcoxon comparisons among temperature groups were corrected using the Benjamini–Hochberg procedure, and effect sizes were estimated using Cliff’s delta with 95% confidence intervals. Although MAG abundance and diversity showed visible differences among temperature categories, pairwise effect sizes were negligible across comparisons (|δ| ≤ 0.097), indicating that these patterns should be interpreted cautiously. For the 18-sample dataset of all available MAGs (116 MAGs with defense systems) Pairwise Wilcoxon comparisons were corrected using the Benjamini-Hochberg (BH) procedure and Cliff's delta. 95% confidence intervals were reported for all pairwise comparisons (all were negligible; |δ| ≤ 0.097). CRISPR-Cas prevalence was quantified by temperature bin (19.0%, 23.1%, and 8.3% for <20°C, 20-45°C, >45°C) and formally tested (Fisher P = 0.166; logistic regression P = 0.254). PERMANOVA: phylum R² = 0.162, P = 0.001; temperature R² = 0.007, P = 0.571 (ns). All PERMDISP tests turned out to be non-significant.

***Supplementary Results***

Summary of sequence proportion passing decontamination steps. Reads retained range from 72 to 99%, with a mean of 90.9%. Reads aligned to controls range from 1 to 28%, with a mean of 9.1%. Sequencing depth: 1.606 billion total, 13.4 to 249.4 M per sample.

| Sample | Raw_Reads | After_Decontam | Pct_Passed_Decontam |
| --- | --- | --- | --- |
| 1547B_1H2 | 159045054 | 158460557 | 99 |
| 1547B_1H3 | 27664813 | 27566880 | 99 |
| 1547B_2H2 | 119373475 | 118819140 | 99 |
| 1547B_2H3 | 22959287 | 22866209 | 99 |
| 1547B_3H3 | 41838996 | 41544275 | 99 |
| 1547B_5H2 | 99353865 | 81822167 | 82 |
| 1547B_5H3 | 24386624 | 17616259 | 72 |
| 1547B_8H2 | 43526662 | 43192007 | 99 |
| 1547B_9H2 | 110148402 | 96805978 | 87 |
| 1547B_9H3 | 27384794 | 20614634 | 75 |
| 1547B-7H3 | 13448521 | 10481384 | 77 |
| 1547D_10R1 | 101928358 | 82847026 | 81 |
| 1548B_2H3 | 129924898 | 129261288 | 99 |
| 1548B_4H7 | 80360682 | 79998155 | 99 |
| 1548B_8H5 | 110546687 | 96881801 | 87 |
| 1548C_1H3 | 249433765 | 249185474 | 99 |
| 1548C_3H6 | 131637655 | 128697511 | 97 |
| 1548C_5H4 | 112983865 | 99387782 | 87 |

**Phylogenetic vs temperature control of defense gene distribution**

- PGLS: Pagel's λ ≈ 0 (1×10⁻⁶; near-zero phylogenetic signal in defense gene count); temperature P = 0.343

- RM systems: nominally positive association in temp-only model (P = 0.016), but fails FDR correction (FDR = 0.052) and disappears with domain covariate (P = 0.305, ns) — domain-level phylogenetic effect

- Abi systems: nominally positive in temp-only (P = 0.012), fails FDR (0.052), disappears with domain (P = 0.919) — phylogenetically driven

- CRISPR-Cas: no significant association in either model (temp-only P = 0.222; temp+domain P = 0.190)

- pAgo: inference remains limited by sample size (n=7); no significant association (temp-only P = 0.368; temp+domain P = 0.202)

- Other systems: nominally positive in temp-only (P = 0.014), fails FDR (0.052), disappears with domain (P = 0.368) — phylogenetically driven

- Overall: after phylogenetic correction and FDR adjustment, no defense system category shows a statistically significant temperature association; patterns are attributable to domain-level phylogenetic structure (see also Supplementary Figure 2)

Results of phylogenetic logistic regression analyses testing associations between defense system occurrence and temperature across Guaymas Basin MAGs.

| defense_type | model | Estimate | std_error | z_value | p_value | alpha | n_present | n_absent | FDR_BH |
| --- | --- | --- | --- | --- | --- | --- | --- | --- | --- |
| Abi | Temperature | 0.022469161 | 0.010209291 | 2.200854071 | 0.027746356 | 0.74770703 | 33 | 38 | 0.080041812 |
| Abi | temperature + domain | -0.000839976 | 0.009143613 | -0.091864783 | 0.926805472 | 0.747142167 | 33 | 38 | 0.926805472 |
| CRISPR_Cas | Temperature | -0.016275362 | 0.013069452 | -1.2452979 | 0.213022267 | 0.747298456 | 10 | 61 | 0.275874453 |
| CRISPR_Cas | temperature + domain | -0.03639415 | 0.017665201 | -2.060217178 | 0.039377783 | 4.306101009 | 10 | 61 | 0.080041812 |
| RM | Temperature | 0.050211251 | 0.017650585 | 2.844735874 | 0.004444826 | 0.748185627 | 45 | 26 | 0.03555861 |
| RM | temperature + domain | 0.027150709 | 0.013854237 | 1.959740468 | 0.050026133 | 40.37648119 | 45 | 26 | 0.080041812 |
| Other | Temperature | 0.018307544 | 0.009295426 | 1.969521836 | 0.048893198 | 0.747303169 | 35 | 36 | 0.080041812 |
| Other | temperature + domain | 0.01382863 | 0.011804015 | 1.171519197 | 0.241390146 | 0.746941423 | 35 | 36 | 0.275874453 |

**Supplementary Tables**

**Table S1**. Sill rock samples used for DNA extraction attempts. Veined rock samples were crushed before DNA extraction using sterile hammers; 20-30 ml of the resulting debris was used for DNA isolation. Resulting DNA quantities were below quantification limit, in the sub-nanogram range.

| Sample | Description | Depth [m] | In-situ Temp. [°C] | Comments |
| --- | --- | --- | --- | --- |
| 1547E-10R1 | Veined rock | 134.5 | 81.8 | Metagenomic library |
| 1547E-15R2 | veined rock pieces 1 and 2 | 155.65 | 92.6 | Metagenomic libraries unsuccessful |
| 1547E-15R2 | Veined rock pieces 4 to 7 | 155.65 | 92.6 |  |
| 1547E-17R3 | Solid rock | 166.35 | 98.1 |  |
| 1547E-19R2 | Solid rock | 174.1 | 102.0 |  |
| 1548A-20X | Solid rock | 101.9 | 74.2 |  |

**Table S2.** Overview on samples yielding prokaryotic argonaute genes (pAgo). MAGs and argonaute genes of sediment samples 1547B-5H2 and 1548B-4H7 were obtained from previously published unassembled genomes (Mara et al. 2023b). Argonaute genes from samples 1547D-10R1 (veined rock, crushed) and 1548C-3H6 (sediment) were obtained from newly isolated and sequenced DNA (this study).

| IODP hole | core & subcore | Sample description | depth[m] CSF-A | in-situ temp[C] | Bin yielding  Argonaute genes | Phylogenetic affiliation |
| --- | --- | --- | --- | --- | --- | --- |
| 1547B | 5H2 | 0.5 g sediment | 36.9 | 31.9 | SRR22580803_  bin.1.fna_00806 | Bathyarchaeaceae  g_SOJZ01 |
| 1547D | 10R1 | veined rock 20 ml | 134.5 | 81.8 | 7_bin.1.fna_01190 | Bathyarchaeota |
| 1548B | 4H7 | 0.5 g sediment | 33.5 | 33.5 | SRR22580798_  bin.19.fna_00701 | Aenigmatarchaeia  o_CG10238-14  f_SCSR01 |
| 1548B | 4H7 | 0.5 g sediment | 33.5 | 33.5 | SRR22580798_  bin.29.fna_01460 | Bathyarchaeales  f_JAYEKF01  g_JAYEKF01 |
| 1548C | 3H6 | 3 x 0.5 g sediment | 24.17 | 39.08 | 12_bin.3.fna_01259  12_bin.3.fna_01260 | Hadarchaeaceae  g_DG-33 |
| 1548C | 3H6 | 3 x 0.5 g sediment | 24.17 | 39.08 | 12_bin.10.fna_01588  12_bin.10.fna_01589 | Aerophobales  f_AE-B3A  g_AE-B3B |
| 1548C | 3H6 | 3 x 0.5 g sediment | 24.17 | 39.08 | 12_bin.11.fna_01717 | Bathyarchaeaceae |

**Table S3.** Cell numbers and extractable DNA content for Ringvent samples (1). Note the sharp decline in cell numbers and DNA yield at ca. 45 to 50°C in-situ temperature.

| **Sample ID** | **Depth (m)** | **Interpolated Temp. (°C)** | **Cells/ml sediment** | **DNA conc.**  **(ng/g sediment)** |
| --- | --- | --- | --- | --- |
| 1547B-1H2 | 2.2 | 14.2 | No data | 300 |
| 1547B-2H2 | 8.7 | 17.5 | 1.25 x 10^8^ | 250 |
| 1547B-3H2 | 17.7 | 22.0 | 9.32 x 10^6^ | 42.86 |
| 1547B-5H2 | 36.9 | 31.9 | 1.35 x 10^6^ | 20.0 |
| 1547B-7H2 | 55.9 | 41.6 | 3.20 x 10^5^ | 2.0 |
| 1547B-8H2 | 65.7 | 46.6 | 4.30 x 10^4^ | 2.0 |
| 1547B-9H2 | 74.3 | 51.0 | 3.85 x 10^4^ | 0.91 |
| 1547B-12F2 | 94.6 | 60.7 | 1.22 x 10^3^ | Below detection limit |
| 1547B-25H2 | 132.1 | 80.6 | 2.77 x 10^3^ | Below detection limit |
| 1548B-1H2 | 2.1 | 8.2 | 5.29 x 10^8^ | 700 |
| 1548B-2H3 | 8.9 | 13.7 | 9.96 x 10^7^ | 135 |
| 1548B-3H4 | 20.4 | 22.9 | 7.68 x 10^7^ | 33.9 |
| 1548B-4H7 | 33.5 | 33.5 | 1.78 x 10^7^ | 10 |
| 1548B-5H5 | 39.6 | 38.3 | 7.52 x 10^6^ | 10 |
| 1548B-6H2 | 46.2 | 43.6 | 1.17 x 10^6^ | 5.9 |
| 1548B-7H3 | 56.4 | 51.8 | No data | No data |
| 1548B-8H5 | 69.5 | 62.4 | 1.01 x 10^3^ | 1.0 |
| 1548B-9H3 | 76.5 | 68.0 | 1.62 x 10^3^ | 2.5 |

**Table S4.** In-situ temperature measurements from Ringvent sites U1547 and U1548. The temperature at the surface (“surface intersect”) is inferred from the linearized best fit of each temperature profile; note that actual surface temperatures measured during *Alvin* dives are cooler, near 3-4°C (3). The data are plotted in Figure 1B. Depth is given in meters below seafloor, mbsf.

| **Site/Hole** | **U1547A** | **U1547B** | **U1548D/E** | **U1548A** | **U1548B** | **U1548C** |
| --- | --- | --- | --- | --- | --- | --- |
| Surface intersect | 13°C | 13°C | 3.25°C | 8.3°C | 6.5°C | 16°C |
| 1^st^ datapoint (T^o^C/depth) | 25.65°C  /33.8m | 27.97°C  /34.7m | 12.21°C  /34.8m | 26.96°C  /30.4m | 33.88°C  /34.4m | 48.45°C  /36.1m |
| 2^nd^ datapoint (T^o^C/depth) | 37.36°C  /52.8m | 40.97°C  /53.7m | 20.86°C  /62.7m | 40.28°C  /49.4m | 49.14°C  /43.4m | 63.38°C  /46.6m |
| 3^rd^ datapoint (T^o^C/depth) | 49.68°C  /71.8m | 53.95°C  /72.7m | 22.33°C  /72.8m | 62.86°C  /87.4m | 59.59°C  /63.9m | 70.46°C  /56.1m |
| 4^th^ datapoint  (T^o^C/depth) | 56.25°C  /90.8m | 60.18°C  /91.7m | 28.78°C  /91.2m |  | 64.34°C  /73.4m | 77.25°C  /65.6m |
| 5^th^ datapoint (T^o^C/depth) | 66.61°C  /96.4m | 66.83°C  /101.9m |  |  |  |  |
| 6^th^ datapoint (T^o^C/depth) | 77.45°C  /115.6m | 74.21°C  /124.2m |  |  |  |  |

**Table S6.** Pairwise Wilcoxon comparisons for MAG read recruitment and MAG alpha diversity (Figure 2) among temperature groups (3-20°C, 20–45°C, >45°C) using the Benjamini–Hochberg procedure.

**Tables S5 , S7-to S9, and S10,** are provided as separate excel files.

**Table** **S5**. Summary and genomic statistics of metagenome-assembled genomes (MAGs) recovered from 17 sediment samples and one rock sample.

**Table** **S7**. Distribution of antiviral defense systems and total defense gene counts per MAG identified using DefenseFinder.

**Table** **S8**. Detailed annotation and distribution of antiviral defense genes across all reconstructed MAGs identified via DefenseFinder.

**Table** **S9**. Distribution of CRISPR-Cas systems and associated genes in MAGs identified via CasFinder.

**Table** **S10**. Presence of genes and protein domains associated with nucleotide-based signaling pathways in metatranscriptomic data [8].

**Supplementary Figures**

**Figure S1.** Phylum composition of quality-filtered reads before MAG recruitment. Several warm or hot samples are particularly rich in Pseudomonadota (1547B-5H2 (31.9°C); 1548B_4H7 (33.5°C); 1547B_8H2 (46.6°C); 1547B_9H2 (51°C); 1548B_8H5 (62.4°C).

**Figure S2**. Prevalence of different defense systems (Abi, CRISPR-Cas, pAgo, RM, others) within MAGs plotted as a function of temperature class, for the archaeal and bacterial domains.

**Figure S3.** MAG community composition beta diversity was analyzed across all 18 samples using Bray-Curtis dissimilarity (stress = 0.037) calculated from RPKM abundance data. NMDS ordinations of MAG community composition showed moderate visual clustering by temperature.

**Figure S4**. Defense system presence vs MAG relative abundance for Abi, CRISPR-Cas, pAgo, RM and other systems. The absence or presence of defense genes in MAGs does not influence MAG abundance.

**Supplementary Discussion: Signaling pathways and their gene expression in the Guaymas Basin deep biosphere**

*Evidence for defense systems under energy limitation*. In the context of prokaryotic defense and its relationship to energy limitation, we report the expression of prokaryotic defense, motility, and chemotaxis genes across the Guaymas subsurface transect at all sites. Independently of drilling site, these processes are most highly expressed between 0.8 and 36.8 mbsf, with a pronounced decline in expression of these genes at greater depths and higher temperatures, consistent with energy limitation effects (see Figure 3 and associated Source Data in ref. 8). Nonetheless, prokaryotic defense systems (grouped via KEGG metabolism) are expressed across all samples, including down to 101.9 mbsf at Site U1546B (25°C). All underlying data are provided in the Source Data. However, KEGG-level annotation does not resolve specific defense systems or proteins, as higher-resolution interpretation requires a dedicated defense gene-level pipeline, as applied in our present work.

*Dormancy and sporulation.* In the Guaymas subsurface cell counts drop drastically below 50 mbsf; with increasing temperature and depth, cells may walk a fine line between utilizing strategies that salvage critical nutrients to remain active, vs. entering stationary-phase, long-term dormancy (e.g., spore formation), or cell death (Fig. 2, ref. 8). Consistent with this interpretation, analysis of Figure 3 and Source Data [ref. 8] shows that sporulation-related genes are expressed in nearly all samples, supporting the widespread presence of dormancy-associated strategies in the subsurface system.

*Evidence for activity of nucleotide-based signaling pathways*. While signaling pathways were examined in the metatranscriptomic dataset [8] they were not emphasized as they represented an emerging aspect of subsurface microbial functioning at the time. We re-examined the available metatranscriptomic datasets and confirmed the presence of genes and protein domains associated with nucleotide-based signaling pathways (Table S10). Specifically, we identified expression of the core gene diguanylate cyclase, which catalyzes the formation of c-di-GMP via lyase activity. This gene is primarily associated with Deltaproteobacteria, Atribacteria, Aminicenantes, Cloacimonetes, and Desulfurispirillum. In addition, we detected expression of the GGDEF domain, a hallmark of diguanylate cyclase activity, associated with Deltaproteobacteria, Atribacteria, Aminicenantes, Sulfurimonas, and Desulfobacteraceae. The concurrent detection of both the gene and its characteristic functional domain provides strong evidence for active c-di-GMP signaling in these subsurface communities. Similarly, we detected expression of (p)ppGpp synthetase, a key enzyme of the stringent response that mediates stress adaptation by downregulating transcription, translation, and central metabolic pathways under nutrient limitation and environmental stress. This gene is primarily associated with Chloroflexi, Atribacteria, Aminicenantes, and Epsilonproteobacteria (Table S10).

***CRISPR-Cas diversity and function*** Beyond its canonical role in cell defense, CRISPR–Cas I and IV may possibly play alternative or derived roles in mediating self-targeting of bacterial or archaeal host DNA [9-11]. Discriminating between these roles – especially in rare or alternative variants - requires a combination of targeted experimental approaches including cultivation, isotope labeling, spacer-level analyses, and controlled infection assays. Self-targeting mechanisms are often associated with gene regulation [12] and genome evolution [13] and can lead to cytotoxicity [14]. But when rearrangements are non-lethal, they have the potential to generate a genetic mosaic that can facilitate rapid niche adaptation [13]. While analyses of viral diversity were outside the scope of our funded Guaymas Basin study, we gained some nominal insight into in situ viral diversity by analyzing CRISPR spacers. Aside from finding that only 2.6% of identified spacer sequences matched known viruses in IMG/VR, we compared spacer sequences to our assembled metagenomic contigs to identify self-targeting spacers. The mean self-hit rate per MAG was 64.9%. In addition to the canonical role of CRISPR in defense against viruses, self-targeting may play a role in gene regulation that confers fitness advantages [12], a concept that can be explored for the subsurface in the future, incorporating bioinformatic mining of self-targeting spacers that are distinct from those matching known phages, viruses, or plasmids [15].

CRISPR-Cas subtype III systems were detected in our data sets within four bacterial MAGs affiliated with Chloroflexota, Desulfobacterota, and TA06 Zixibacteria from warm, shallow sediments (up to 18.6°C; 8.9 mbsf). Compared to CRISPR-Cas subtypes I and IV found in Ringvent, CRISPR-Cas III appears even more restricted to specific depths and temperatures (Figure 4; Table S7 and S8). This pattern may reflect either lower abundance of this subtype, or lower activity/abundance of the viruses targeted by CRISPR-Cas III at greater depths and higher temperatures (restriction of CRISPR-Cas III to the upper, less thermally stressed sediment layers). As suggested for CRISPR-Cas I and IV [16]. CRISPR-Cas III may also perform additional roles in the hydrothermal subsurface beyond antiphage defense. CRISPR-Cas subtype III functions through a secondary messenger (cyclic oligoadenylate; cOA) that degrades the RNA of the invader but also the mRNA of the host, interfering with growth of both the virus and the host [17]. This non-specific effect of CRISPR-Cas subtype III may reflect the ancestral function of CRISPR-Cas systems that use a nucleotide-based stress-signaling pathway, similar to the cOA-signaling pathway, to induce RNA degradation [18]. CRISPR-Cas systems evolved to be defense systems against viral RNA later, but they retained their ability to also degrade the host’s mRNA. Degrading host mRNA can lead to dormancy or arrest of cellular growth as a response to viral attack. Because the ancestral function of the CRISPR-Cas systems is retained, and because these systems are activated under nucleotide-based stress-signaling pathways, we suggest that the CRISPR-Cas systems for RNA and single stranded DNA degradation found in Guaymas Basin could be mechanisms involved in regulating gene expression (degrade/silence mRNA) and arresting cellular growth under viral but also under non-viral stress, via nucleotide based signaling, as documented in the Guaymas deep transcriptome. Many nucleotide-based pathways (e.g., c-di-GMP and (p)ppGpp) are conserved stress-sensing and signal-transduction mechanisms in prokaryotes that act as second messengers [19]. By conveying information about environmental changes to trigger appropriate cellular responses [20], they link environmental perception to specific behaviors in prokaryotes, including extremophilic lineages [21]. Responses can include regulation of biofilm formation, motility, and starvation-induced growth attenuation. Nucleotide-based signaling may be more feasible in the upper sediment layers, as these mechanisms likely require higher cell densities to effectively coordinate population-level responses (higher cell densities exist in the shallower sediments in Ringvent; Table S3). Further investigation into RNA-guided cyclic oligoadenylate (cOA) signaling could reveal connections between CRISPR–Cas immunity and broader nucleotide-signaling pathways as mechanisms of survival in Guaymas hydrothermal subsurface.

**Supplementary References**

1. Teske A, Lizarralde D, Höfig TW, Aiello IW, Ash JL, Bojanova DP, et al. Expedition 385 summary. *In* Teske, A., Lizarralde, D., Höfig, T.W., and the Expedition 385 Scientists, *Guaymas Basin Tectonics and Biosphere*. Proceedings of the International Ocean Discovery Program, 385: College Station, TX (International Ocean Discovery Program). 2021. <https://doi.org/10.14379/iodp.proc.385.101.2021>

2. Teske A, Lizarralde D, Höfig TW, Aiello IW, Ash JL, Bojanova DP, et al. Sites U1547 and U1548. In Teske, A., Lizarralde, D., Höfig, T.W., and the Expedition 385 Scientists, Guaymas Basin Tectonics and Biosphere. Proceedings of the International Ocean Discovery Program, 385: College Station, TX (International Ocean Discovery Program). 2021. [doi:10.14379/iodp.proc.385.105.2021](https://doi.org/10.14379/iodp.proc.385.105.2021).

3. Teske A, McKay LJ, Ravelo AC, Aiello I, Mortera C, Núñez-Useche F, et al. Characteristics and Evolution of sill-driven off-axis hydrothermalism in Guaymas Basin - the Ringvent site. *Sci Rep*. 2019;9(1):13847. doi:10.1038/s41598-019-50200-5.

4. Galerne C, Cheviet A, Kahl W-A, Wiggers C, Bach W, Neumann F, et al. Sill stacking in subseafloor unconsolidated sediments and control on sustained hydrothermal systems: Evidence from IODP drilling in the Guaymas Basin, Gulf of California. *Journal of Geophysical Research: Solid Earth*. 2025; 130(3), e2024JB030354. doi:10.1029/2024JB030354.

5. Ramírez GA, Mara P, Beaudoin D, Bojanova D, Hinkle JE, Kingham B, et al. Data report: the DNA event horizon in the Guaymas Basin subsurface biosphere: technical advances and redefined limits in bulk extractions of nucleic acids from deep marine sediments, IODP Expedition 385. In Teske, A., Lizarralde, D., Höfig, T.W., and the Expedition 385 Scientists, Guaymas Basin Tectonics and Biosphere. Proceedings of the International Ocean Discovery Program, 385: College Station, TX (International Ocean Discovery Program). 2024.

6. Mara P, Beaudoin D, Aiello I, Morono Y, Geller-McGrath D, Edgcomb VP, et al. Deep subseafloor sediments in Guaymas Basin harbor cosmopolitan microbiota and traces of hydrothermal populations. *Comm, Earth & Environ*. 2024; 5, 505. doi: 10.1038/s43247-024-01662-7.

7. Hinkle JE, Mara P, Beaudoin DJ, Edgcomb VP, Teske AP. A PCR-Based Survey of Methane-Cycling Archaea in Methane-Soaked Subsurface Sediments of Guaymas Basin, Gulf of California. *Microorganisms*. 2023;11(12):2956. doi:10.3390/microorganisms11122956

8. Mara P, Zhou YL, Teske A, Morono Y, Beaudoin D, Edgcomb V. Microbial gene expression in Guaymas Basin subsurface sediments responds to hydrothermal stress and energy limitation. *ISME J*. 2023;17(11):1907-1919. doi:10.1038/s41396-023-01492-z.

9. Stern A, Keren L, Wurtzel O, Amitai G, Sorek R. Self-targeting by CRISPR: gene regulation or autoimmunity?. *Trends Genet*. 2010;26(8):335-340. doi:10.1016/j.tig.2010.05.008.

10. Brodt A, Lurie-Weinberger MN, Gophna U. CRISPR loci reveal networks of gene exchange in archaea. *Biol Direct*. 2011;6(1):65. doi:10.1186/1745-6150-6-65.

11. Marraffini LA, Sontheimer EJ. CRISPR interference: RNA-directed adaptive immunity in bacteria and archaea. *Nat Rev Genet*. 2010;11(3):181-190. doi:10.1038/nrg2749.

12. Wimmer F, Beisel CL. CRISPR-Cas Systems and the Paradox of Self-Targeting Spacers. *Front Microbiol*. 2020;10:3078. doi:10.3389/fmicb.2019.03078.

13. Shabbir MA, Hao H, Shabbir MZ, Wu Q, Sattar A, Yuan Z. Bacteria vs. Bacteriophages: Parallel Evolution of Immune Arsenals. *Front Microbiol*. 2016;7:1292. doi:10.3389/fmicb.2016.01292.

14. Vercoe RB, Chang JT, Dy RL, Taylor C, Gristwood T, Clulow JS, et al. Cytotoxic chromosomal targeting by CRISPR/Cas systems can reshape bacterial genomes and expel or remodel pathogenicity islands. *PLoS Genet*. 2013;9(4):e1003454. doi:10.1371/journal.pgen.1003454.

15. Haft DH, Selengut J, Mongodin EF, Nelson KE. A guild of 45 CRISPR-associated (Cas) protein families and multiple CRISPR/Cas subtypes exist in prokaryotic genomes. *PLoS Comput Biol*. 2005;1(6):e60. doi:10.1371/journal.pcbi.0010060.

16. Makarova KS, Shmakov SA, Wolf YI, Mutz P, Altae-Tran H, Beisel CL, et al. An updated evolutionary classification of CRISPR-Cas systems including rare variants. *Nat Microbiol*. 2025;10(12):3346-3361. doi:10.1038/s41564-025-02180-8.

17. Rostøl JT, Marraffini LA. Non-specific degradation of transcripts promotes plasmid clearance during type III-A CRISPR-Cas immunity. *Nat Microbiol*. 2019;4(4):656-662. doi:10.1038/s41564-018-0353-x.

18. Koonin EV, Makarova KS. Origins and evolution of CRISPR-Cas systems. *Philos Trans R Soc Lond B Biol Sci*. 2019;374(1772):20180087. doi:10.1098/rstb.2018.0087.

19. Hengge R, Pruteanu M, Stülke J, Tschowri N, Turgay K. Recent advances and perspectives in nucleotide second messenger signaling in bacteria. *Microlife*. 2023;4:uqad015. doi:10.1093/femsml/uqad015.

20.. Gottesman S. Trouble is coming: Signaling pathways that regulate general stress responses in bacteria. *J Biol Chem*. 2019;294(31):11685-11700. doi:10.1074/jbc.REV119.005593.

21. Moya-Beltrán A, Rojas-Villalobos C, Díaz M, Guiliani N, Quatrini R, Castro M. Nucleotide second messenger-based signaling in extreme acidophiles of the *Acidithiobacillus* species complex: partition between the core and variable gene complements. *Front Microbiol*. 2019;10:381. doi:10.3389/fmicb.2019.00381.
