# Supplementary material for: From CRISPR–Cas to Argonautes: Defense systems of bacteria and archaea in the hydrothermal deep subsurface
Source: ISME Commun. 2026 Jul 10;6(1):ycag200. doi: 10.1093/ismeco/ycag200 (PMC13477220; doi:10.1093/ismeco/ycag200)
Supplement: Supplementary file 1 [file supplementary_material_ycag200.zip › Supplementary_Materials_ycag200.zip!/_7_bin_1_fna-_01190_hypothetical_protein.pdf]

Phyre2.2

Email paraskevi.mara@gmail.com
Description _7_bin_1_fna-_01190_hypothetical_protein
Date Mon Dec 22 19:11:28 GMT 2025
Unique Job ID 6493df43d75a14b5

Detailed template information

#	Template	Alignment Coverage	3D Model	Confidence	% i.d.	Template Information
1	c2f8sC_	Alignment	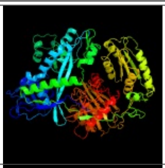	99.9	17	**PDB header:** rna binding protein/rna<br>**Chain:** C: **PDB Molecule:** Argonaute protein<br>**PDBTitle:** crystal structure of aa-ago with externally-bound sirna<br>**PDB Entry:** PDBe RCSB PDBj
2	c8xihB_	Alignment	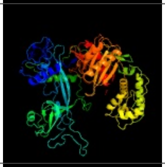	100.0	17	**PDB header:** dna binding protein/dna<br>**Chain:** B: **PDB Molecule:** Piwi domain-containing protein<br>**PDBTitle:** protein-dna complex<br>**PDB Entry:** PDBe RCSB PDBj
3	c4kreA_	Alignment	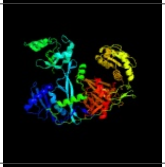	99.8	16	**PDB header:** transcription/rna<br>**Chain:** A: **PDB Molecule:** Protein argonaute-1<br>**PDBTitle:** structure of human argonaute-1 bound to endogenous sf9 rna<br>**PDB Entry:** PDBe RCSB PDBj
4	c7r8iA_	Alignment	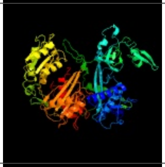	100.0	16	**PDB header:** hydrolase/dna<br>**Chain:** A: **PDB Molecule:** Argonaute<br>**PDBTitle:** ;crystal structure of pseudooceanicola lipolyticus argonaute bound to 5 oh guide dna in the presence of mg2+;<br>**PDB Entry:** PDBe RCSB PDBj
5	c7r8gA_	Alignment	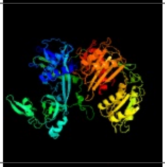	100.0	16	**PDB header:** hydrolase/dna<br>**Chain:** A: **PDB Molecule:** Argonaute<br>**PDBTitle:** ;crystal structure of pseudooceanicola lipolyticus argonaute bound to 5 oh guide dna;<br>**PDB Entry:** PDBe RCSB PDBj
6	c7svaA_	Alignment	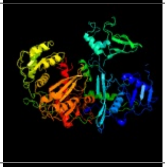	99.7	16	**PDB header:** gene regulation<br>**Chain:** A: **PDB Molecule:** Protein argonaute 10<br>**PDBTitle:** cryo-em structure of arabidopsis ago10-guide rna complex<br>**PDB Entry:** PDBe RCSB PDBj
7	c6mdzA_	Alignment	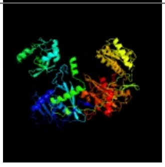	99.8	15	**PDB header:** hydrolase/rna<br>**Chain:** A: **PDB Molecule:** Protein argonaute-2<br>**PDBTitle:** human argonaute2-mir-122 bound to a target rna with two central mismatches (bu2)<br>**PDB Entry:** PDBe RCSB PDBj
8	c1yvuA_	Alignment	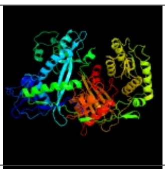	99.9	16	**PDB header:** rna binding protein, gene regulation<br>**Chain:** A: **PDB Molecule:** hypothetical protein aq_1447<br>**PDBTitle:** crystal structure of a. aeolicus argonaute<br>**PDB Entry:** PDBe RCSB PDBj
9	c4w5tA_	Alignment	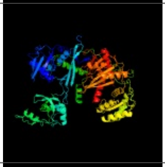	99.8	15	**PDB header:** hydrolase/rna<br>**Chain:** A: **PDB Molecule:** Protein argonaute-2<br>**PDBTitle:** the crystal structure of human argonaute2 bound to a guide and target rna containing seed pairing from 2-7<br>**PDB Entry:** PDBe RCSB PDBj
10	c4ncbA_	Alignment	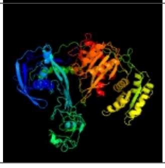	100.0	16	**PDB header:** nuclear protein/dna<br>**Chain:** A: **PDB Molecule:** Argonaute<br>**PDBTitle:** structure of thermus thermophilus argonaute bound to guide dna and 19-mer target dna with mg2+<br>**PDB Entry:** PDBe RCSB PDBj

11	c6nitA_	Alignment	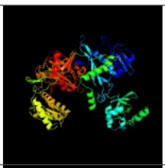	99.8	15	**PDB header:** rna binding protein/rna<br>**Chain:** A: **PDB Molecule:** Protein argonaute-2<br>**PDBTitle:** human argonaute2-mir-122 bound to a target rna with four central mismatches (bu4)<br>**PDB Entry:** PDBe RCSB PDBj
12	c4z4dA_	Alignment	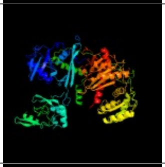	99.7	15	**PDB header:** gene regulation/rna<br>**Chain:** A: **PDB Molecule:** Protein argonaute-2<br>**PDBTitle:** human argonaute2 bound to t1-g target rna<br>**PDB Entry:** PDBe RCSB PDBj
13	c6oonA_	Alignment	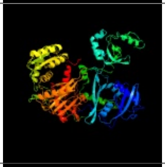	99.7	15	**PDB header:** hydrolase/rna<br>**Chain:** A: **PDB Molecule:** Protein argonaute-4<br>**PDBTitle:** human argonaute4 bound to guide rna<br>**PDB Entry:** PDBe RCSB PDBj
14	c7yfqC_	Alignment	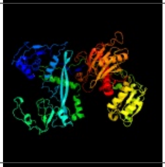	99.8	15	**PDB header:** rna binding protein/rna<br>**Chain:** C: **PDB Molecule:** Piwi<br>**PDBTitle:** cryo-em structure of the efpiwi (n959k)-pirna-target ternary complex<br>**PDB Entry:** PDBe RCSB PDBj
15	c7kx9A_	Alignment	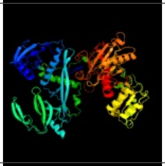	99.8	15	**PDB header:** hydrolase,rna binding protein/rna<br>**Chain:** A: **PDB Molecule:** Piwi-A<br>**PDBTitle:** cryo-em structure of ephydatia fluviatilis piwia-pirna-target complex<br>**PDB Entry:** PDBe RCSB PDBj
16	c3dlbA_	Alignment	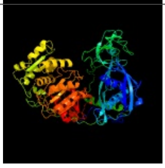	99.9	16	**PDB header:** nucleic acid binding protein/dna<br>**Chain:** A: **PDB Molecule:** argonaute<br>**PDBTitle:** crystal structure of the guide-strand-containing argonaute protein silencing complex<br>**PDB Entry:** PDBe RCSB PDBj
17	c1u04A_	Alignment	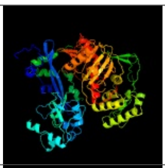	99.9	17	**PDB header:** hydrolase/gene regulation<br>**Chain:** A: **PDB Molecule:** hypothetical protein PF0537<br>**PDBTitle:** crystal structure of full length argonaute from pyrococcus furiosus<br>**PDB Entry:** PDBe RCSB PDBj
18	c8vajA_	Alignment	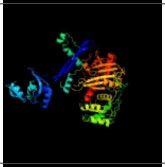	99.7	17	**PDB header:** rna binding protein/rna<br>**Chain:** A: **PDB Molecule:** Protein argonaute-3<br>**PDBTitle:** human argonaute3 bound to cityrna and target rna<br>**PDB Entry:** PDBe RCSB PDBj
19	c6n4oA_	Alignment	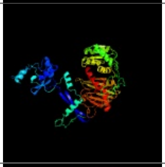	99.6	17	**PDB header:** rna binding protein/rna<br>**Chain:** A: **PDB Molecule:** Protein argonaute-2<br>**PDBTitle:** human argonaute2-mir-122 bound to a seed and supplementary paired target<br>**PDB Entry:** PDBe RCSB PDBj
20	c5g5sA_	Alignment	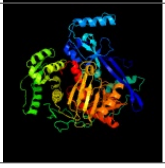	99.9	17	**PDB header:** hydrolase<br>**Chain:** A: **PDB Molecule:** ARGONAUTE<br>**PDBTitle:** structure of the argonaute protein from methanocaldcoccus janaschii<br>**PDB Entry:** PDBe RCSB PDBj
21	c5guhA_	Alignment	not modelled	99.8	13	**PDB header:** hydrolase/rna<br>**Chain:** A: **PDB Molecule:** PIWI<br>**PDBTitle:** crystal structure of silkworm piwi-clade argonaute siwi bound to pirna<br>**PDB Entry:** PDBe RCSB PDBj
22	c5vm9C_	Alignment	not modelled	99.6	16	**PDB header:** hydrolase/rna<br>**Chain:** C: **PDB Molecule:** Protein argonaute-3<br>**PDBTitle:** human argonaute3 bound to guide rna<br>**PDB Entry:** PDBe RCSB PDBj
23	c6kr6A_	Alignment	not modelled	99.8	13	**PDB header:** rna binding protein/rna<br>**Chain:** A: **PDB Molecule:** Protein piwi<br>**PDBTitle:** crystal structure of drosophila piwi<br>**PDB Entry:** PDBe RCSB PDBj
24	c7yfxA_	Alignment	not modelled	99.7	15	**PDB header:** rna binding protein/rna<br>**Chain:** A: **PDB Molecule:** Piwi-like protein 2<br>**PDBTitle:** cryo-em structure of hili in complex with pirna<br>**PDB Entry:** PDBe RCSB PDBj

25	c8wd8A_	Alignment	not modelled	99.9	15	**PDB header:** dna binding protein/dna<br>**Chain:** A: **PDB Molecule:** Argonaute family protein<br>**PDBTitle:** cryo-em structure of ttdago-guide dna-target dna complex<br>**PDB Entry:** PDBe RCSB PDBj
26	c8jpxD_	Alignment	not modelled	99.9	15	**PDB header:** gene regulation<br>**Chain:** D: **PDB Molecule:** Protein argonaute<br>**PDBTitle:** cryo-em structure of pfago-guide dna-target dna complex<br>**PDB Entry:** PDBe RCSB PDBj
27	c8jpxA_	Alignment	not modelled	99.9	15	**PDB header:** gene regulation<br>**Chain:** A: **PDB Molecule:** Protein argonaute<br>**PDBTitle:** cryo-em structure of pfago-guide dna-target dna complex<br>**PDB Entry:** PDBe RCSB PDBj
28	c8u72D_	Alignment	not modelled	99.9	20	**PDB header:** rna binding protein/rna/dna<br>**Chain:** D: **PDB Molecule:** Piwi domain-containing protein<br>**PDBTitle:** cryo-em structure of the sparta oligomer with guide rna and target dna<br>**PDB Entry:** PDBe RCSB PDBj
29	c8fexB_	Alignment	not modelled	99.9	19	**PDB header:** immune system<br>**Chain:** B: **PDB Molecule:** short pAgo<br>**PDBTitle:** inactivate state of maribacter polysiphoniae argonuate (short pago system)<br>**PDB Entry:** PDBe RCSB PDBj
30	c8ffiB_	Alignment	not modelled	99.9	19	**PDB header:** immune system<br>**Chain:** B: **PDB Molecule:** short pAgo<br>**PDBTitle:** structure of tetramerized mapsparta upon guide rna-mediated target dna binding<br>**PDB Entry:** PDBe RCSB PDBj
31	c8ifkB_	Alignment	not modelled	99.9	18	**PDB header:** rna binding protein/rna/dna<br>**Chain:** B: **PDB Molecule:** Piwi domain-containing protein<br>**PDBTitle:** cryo-em structure of monomeric sparta grna-ssdna target complex<br>**PDB Entry:** PDBe RCSB PDBj
32	c8k88C_	Alignment	not modelled	99.9	17	**PDB header:** dna/rna/cell invasion<br>**Chain:** C: **PDB Molecule:** Piwi domain protein<br>**PDBTitle:** structure of procaryotic ago<br>**PDB Entry:** PDBe RCSB PDBj
33	c8jl0B_	Alignment	not modelled	99.9	16	**PDB header:** antiviral protein/dna/rna<br>**Chain:** B: **PDB Molecule:** Piwi domain protein<br>**PDBTitle:** cryo-em structure of the prokaryotic sparsa system complex<br>**PDB Entry:** PDBe RCSB PDBj
34	c8in8B_	Alignment	not modelled	99.9	16	**PDB header:** antiviral protein<br>**Chain:** B: **PDB Molecule:** Piwi domain protein<br>**PDBTitle:** cryo-em structure of the target ssdna-bound sir2-apaz/ago-grna quaternary complex<br>**PDB Entry:** PDBe RCSB PDBj
35	c6d92D_	Alignment	not modelled	99.8	16	**PDB header:** rna binding protein<br>**Chain:** D: **PDB Molecule:** Uncharacterized protein<br>**PDBTitle:** ternary rsago complex with guide rna and target dna containing a-a non-canonical pair at position 3<br>**PDB Entry:** PDBe RCSB PDBj
36	c6d92A_	Alignment	not modelled	99.8	16	**PDB header:** rna binding protein<br>**Chain:** A: **PDB Molecule:** Uncharacterized protein<br>**PDBTitle:** ternary rsago complex with guide rna and target dna containing a-a non-canonical pair at position 3<br>**PDB Entry:** PDBe RCSB PDBj
37	c8isyA_	Alignment	not modelled	99.8	22	**PDB header:** dna binding protein<br>**Chain:** A: **PDB Molecule:** Piwi domain-containing protein<br>**PDBTitle:** cryo-em structure of free-state crt-sparta<br>**PDB Entry:** PDBe RCSB PDBj
38	c9iizA_	Alignment	not modelled	99.5	18	**PDB header:** rna binding protein/rna<br>**Chain:** A: **PDB Molecule:** Piwi<br>**PDBTitle:** cryo-em structure of efpiwi-pirna-target (25-nt, comma)<br>**PDB Entry:** PDBe RCSB PDBj
39	c8pvvA_	Alignment	not modelled	99.8	15	**PDB header:** dna binding protein<br>**Chain:** A: **PDB Molecule:** Piwi protein<br>**PDBTitle:** archaeoglobus fulgidus afago complex with afago-n protein (fafago) bound with 30 nt rna guide and 51 nt dna target<br>**PDB Entry:** PDBe RCSB PDBj
40	c8qg0A_	Alignment	not modelled	99.8	15	**PDB header:** dna binding protein<br>**Chain:** A: **PDB Molecule:** Piwi protein<br>**PDBTitle:** archaeoglobus fulgidus afago complex with afago-n protein (fafago) bound with 17 nt rna guide and 17 nt dna target<br>**PDB Entry:** PDBe RCSB PDBj
41	c5theG_	Alignment	not modelled	99.2	17	**PDB header:** rna binding protein/rna<br>**Chain:** G: **PDB Molecule:** Uncharacterized protein<br>**PDBTitle:** crystal structure of the c-terminal lobe of a budding yeast argonaute<br>**PDB Entry:** PDBe RCSB PDBj
42	c5theE_	Alignment	not modelled	99.1	17	**PDB header:** rna binding protein/rna<br>**Chain:** E: **PDB Molecule:** Uncharacterized protein<br>**PDBTitle:** crystal structure of the c-terminal lobe of a budding yeast argonaute<br>**PDB Entry:** PDBe RCSB PDBj
43	c6t5tA_	Alignment	not modelled	99.8	15	**PDB header:** dna binding protein<br>**Chain:** A: **PDB Molecule:** Piwi protein AF_1318<br>**PDBTitle:** ;crystal structure of archaeoglobus fulgidus argonaute protein with cognate dna oligoduplex 5-pattgtggccacaat;<br>**PDB Entry:** PDBe RCSB PDBj
44	c5i4aC_	Alignment	not modelled	99.6	13	**PDB header:** rna binding protein/rna<br>**Chain:** C: **PDB Molecule:** Argonaute protein<br>**PDBTitle:** ;x-ray crystal structure of marinitoga piezophila argonaute in complex with 5 oh guide rna;<br>**PDB Entry:** PDBe RCSB PDBj
45	c9ij2A_	Alignment	not modelled	99.1	15	**PDB header:** rna binding protein/rna<br>**Chain:** A: **PDB Molecule:** Piwi-like protein 2<br>**PDBTitle:** cryo-em structure of mili-pirna-target (22-nt, comma)<br>**PDB Entry:** PDBe RCSB PDBj

46	c2yhaA_	Alignment	not modelled	98.2	12	**PDB header:** rna binding protein<br>**Chain:** A: **PDB Molecule:** POST-TRANSCRIPTIONAL GENE SILENCING PROTEIN QDE-2<br>**PDBTitle:** crystal structure of the n. crassa qde-2 ago mid-piwi domains<br>**PDB Entry:** PDBe RCSB PDBj
47	c3vnbA_	Alignment	not modelled	94.8	17	**PDB header:** rna binding protein<br>**Chain:** A: **PDB Molecule:** Protein argonaute 1<br>**PDBTitle:** structural insights into small rna sorting and mrna binding by arabidopsis ago domains<br>**PDB Entry:** PDBe RCSB PDBj
48	c4g0oA_	Alignment	not modelled	96.2	16	**PDB header:** gene regulation<br>**Chain:** A: **PDB Molecule:** Protein argonaute 5<br>**PDBTitle:** crystal structure of arabidopsis thaliana ago5 mid domain<br>**PDB Entry:** PDBe RCSB PDBj
49	c2qh9A_	Alignment	not modelled	51.5	32	**PDB header:** structural genomics, unknown function<br>**Chain:** A: **PDB Molecule:** UPF0215 protein AF_1433<br>**PDBTitle:** the crystal structure of a protein of unknown function from archaeoglobus fulgidus dsm 4304<br>**PDB Entry:** PDBe RCSB PDBj
