# Supplementary material for: From CRISPR–Cas to Argonautes: Defense systems of bacteria and archaea in the hydrothermal deep subsurface
Source: ISME Commun. 2026 Jul 10;6(1):ycag200. doi: 10.1093/ismeco/ycag200 (PMC13477220; doi:10.1093/ismeco/ycag200)
Supplement: Supplementary file 1 [file supplementary_material_ycag200.zip › Supplementary_Materials_ycag200.zip!/_12_bin_3_fna-_01260_hypothetical_protein.pdf]

Phyre2.2

Email	paraskevi.mara@gmail.com
Description	_12_bin_3_fna-_01260_hypothetical_protein
Date	Mon Dec 22 19:13:47 GMT 2025
Unique Job ID	753398719d8279aa

Detailed template information

#	Template	Alignment Coverage	3D Model	Confidence	% i.d.	Template Information
1	c8isyB_	Alignment	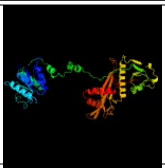	99.9	20	**PDB header:** dna binding protein<br>**Chain:** B: **PDB Molecule:** TIR domain-containing protein<br>**PDBTitle:** cryo-em structure of free-state crt-sparta<br>**PDB Entry:** PDBe RCSB PDBj
2	c8y6pA_	Alignment	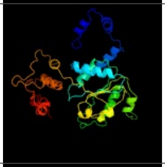	99.0	13	**PDB header:** apoptosis<br>**Chain:** A: **PDB Molecule:** Apaf-1 related killer DARK<br>**PDBTitle:** structure of the auto-inhibited dark monomer<br>**PDB Entry:** PDBe RCSB PDBj
3	c8u72C_	Alignment	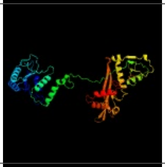	99.9	20	**PDB header:** rna binding protein/rna/dna<br>**Chain:** C: **PDB Molecule:** TIR domain-containing protein<br>**PDBTitle:** cryo-em structure of the sparta oligomer with guide rna and target dna<br>**PDB Entry:** PDBe RCSB PDBj
4	c8ffiM_	Alignment	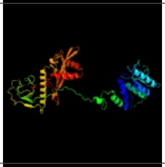	99.9	19	**PDB header:** immune system<br>**Chain:** M: **PDB Molecule:** TIR-APAZ<br>**PDBTitle:** structure of tetramerized mapsparta upon guide rna-mediated target dna binding<br>**PDB Entry:** PDBe RCSB PDBj
5	c7dfvA_	Alignment	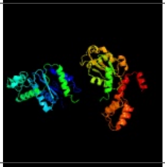	99.9	12	**PDB header:** plant protein<br>**Chain:** A: **PDB Molecule:** NAD+ hydrolase (NADase)<br>**PDBTitle:** cryo-em structure of plant nlr rpp1 tetramer core part<br>**PDB Entry:** PDBe RCSB PDBj
6	c7crcB_	Alignment	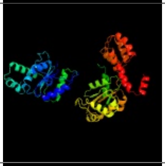	100.0	12	**PDB header:** plant protein<br>**Chain:** B: **PDB Molecule:** NAD+ hydrolase (NADase)<br>**PDBTitle:** cryo-em structure of plant nlr rpp1 tetramer in complex with atr1<br>**PDB Entry:** PDBe RCSB PDBj
7	c8ozeC_	Alignment	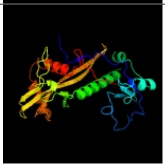	98.1	19	**PDB header:** immune system<br>**Chain:** C: **PDB Molecule:** TIR domain-containing protein<br>**PDBTitle:** cryoem structure of sparta complex dimer high resolution<br>**PDB Entry:** PDBe RCSB PDBj
8	c7nagA_	Alignment	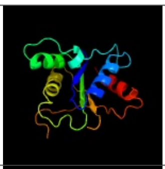	99.0	28	**PDB header:** hydrolase<br>**Chain:** A: **PDB Molecule:** Sterile alpha and TIR motif-containing protein 1<br>**PDBTitle:** crystal structure of the tir domain from human sarm1 in complex with 1ad<br>**PDB Entry:** PDBe RCSB PDBj
9	c8d0jA_	Alignment	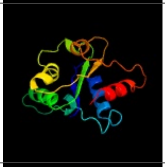	99.0	28	**PDB header:** hydrolase<br>**Chain:** A: **PDB Molecule:** NAD(+) hydrolase SARM1<br>**PDBTitle:** apo human sarm1 tir domain<br>**PDB Entry:** PDBe RCSB PDBj
10	c3j9lP_	Alignment	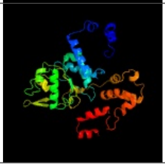	99.5	12	**PDB header:** apoptosis<br>**Chain:** P: **PDB Molecule:** Apaf-1 related killer DARK<br>**PDBTitle:** structure of dark apoptosome from drosophila melanogaster<br>**PDB Entry:** PDBe RCSB PDBj

11	c8g83B_	Alignment	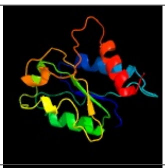	99.0	25	**PDB header:** hydrolase<br>**Chain:** B: **PDB Molecule:** NAD(+) hydrolase AbTIR<br>**PDBTitle:** structure of nad+ consuming protein acinetobacter baumannii tir domain<br>**PDB Entry:** PDBe RCSB PDBj
12	c7uwgC_	Alignment	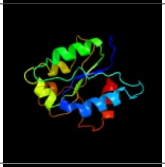	99.1	25	**PDB header:** hydrolase<br>**Chain:** C: **PDB Molecule:** Molecular chaperone Tir<br>**PDBTitle:** the crystal structure of the tir domain-containing protein from acinetobacter baumannii (abtir)<br>**PDB Entry:** PDBe RCSB PDBj
13	c7uwgD_	Alignment	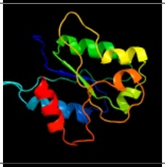	99.1	25	**PDB header:** hydrolase<br>**Chain:** D: **PDB Molecule:** Molecular chaperone Tir<br>**PDBTitle:** the crystal structure of the tir domain-containing protein from acinetobacter baumannii (abtir)<br>**PDB Entry:** PDBe RCSB PDBj
14	c8p2mA_	Alignment	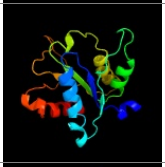	99.0	25	**PDB header:** hydrolase<br>**Chain:** A: **PDB Molecule:** NAD(+) hydrolase tir-1<br>**PDBTitle:** c. elegans tir-1 protein.<br>**PDB Entry:** PDBe RCSB PDBj
15	c7anwD_	Alignment	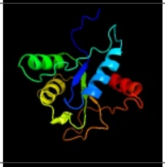	99.3	25	**PDB header:** apoptosis<br>**Chain:** D: **PDB Molecule:** NAD(+) hydrolase SARM1<br>**PDBTitle:** hsarm1 nad+ complex<br>**PDB Entry:** PDBe RCSB PDBj
16	c7knqD_	Alignment	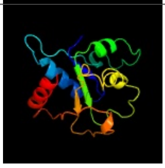	99.1	26	**PDB header:** hydrolase<br>**Chain:** D: **PDB Molecule:** NAD(+) hydrolase SARM1<br>**PDBTitle:** sarm1 octamer<br>**PDB Entry:** PDBe RCSB PDBj
17	c7uxrA_	Alignment	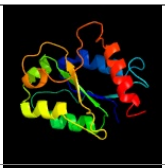	99.0	23	**PDB header:** hydrolase<br>**Chain:** A: **PDB Molecule:** TIR domain protein<br>**PDBTitle:** crystal structure of the bttir tir domain<br>**PDB Entry:** PDBe RCSB PDBj
18	c5ku7A_	Alignment	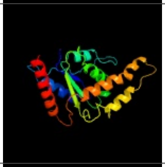	98.8	18	**PDB header:** signaling protein<br>**Chain:** A: **PDB Molecule:** TIR-NB-LRR type resistance protein RPV1<br>**PDBTitle:** crystal structure of the tir domain from the muscadinia rotundifolia disease resistance protein rpv1<br>**PDB Entry:** PDBe RCSB PDBj
19	c6o0wA_	Alignment	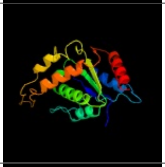	98.8	18	**PDB header:** signaling protein<br>**Chain:** A: **PDB Molecule:** TIR-NB-LRR type resistance protein RUN1<br>**PDBTitle:** crystal structure of the tir domain from the grapevine disease resistance protein run1 in complex with nadp+ and bis-tris<br>**PDB Entry:** PDBe RCSB PDBj
20	c3shfA_	Alignment	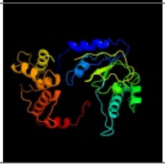	98.9	12	**PDB header:** apoptosis<br>**Chain:** A: **PDB Molecule:** Apoptotic peptidase activating factor 1<br>**PDBTitle:** crystal structure of the r265s mutant of full-length murine apaf-1<br>**PDB Entry:** PDBe RCSB PDBj
21	c4lqdA_	Alignment	not modelled	99.6	25	**PDB header:** immune system<br>**Chain:** A: **PDB Molecule:** Toll/interleukin-1 receptor domain-containing adapter protein<br>**PDBTitle:** ;the crystal structures of the brucella protein tcpb and the tlr adaptor protein tirap show structural differences in microbial tir mimicry;<br>**PDB Entry:** PDBe RCSB PDBj
22	c6wt7A_	Alignment	not modelled	99.5	19	**PDB header:** immune system<br>**Chain:** A: **PDB Molecule:** Metazoan TIR-STING fusion<br>**PDBTitle:** ;structure of a metazoan tir-sting receptor from c. gigas in complex with 2,3-cgamp;<br>**PDB Entry:** PDBe RCSB PDBj
23	c7vu8A_	Alignment	not modelled	99.1	17	**PDB header:** hydrolase<br>**Chain:** A: **PDB Molecule:** Flax rust resistance protein<br>**PDBTitle:** l7-tir domain with bound ligand<br>**PDB Entry:** PDBe RCSB PDBj
24	c7x5lC_	Alignment	not modelled	99.1	17	**PDB header:** hydrolase/dna<br>**Chain:** C: **PDB Molecule:** Flax rust resistance protein<br>**PDBTitle:** tir-dsdna complex, the initial binding state<br>**PDB Entry:** PDBe RCSB PDBj

25	c4w8hA_	Alignment	not modelled	99.4	22	**PDB header:** signaling protein<br>**Chain:** A: **PDB Molecule:** Toll-receptor-related 2<br>**PDBTitle:** ;crystal structure of the tir domain of the toll-related receptor trr-2 from the lower metazoan hydra magnipapillata (crystal form ii);<br>**PDB Entry:** PDBe RCSB PDBj
26	c7ylfA_	Alignment	not modelled	99.3	21	**PDB header:** immune system<br>**Chain:** A: **PDB Molecule:** Toll-like receptor 15<br>**PDBTitle:** crystal structure of the chicken toll-like receptor 15 tir domain (2-mercaptoethanol adduct)<br>**PDB Entry:** PDBe RCSB PDBj
27	c9fp6D_	Alignment	not modelled	99.4	9	**PDB header:** immune system<br>**Chain:** D: **PDB Molecule:** NRC2a<br>**PDBTitle:** structure of the nbnrc2 hexameric resistosome<br>**PDB Entry:** PDBe RCSB PDBj
28	c7ylgA_	Alignment	not modelled	99.3	21	**PDB header:** immune system<br>**Chain:** A: **PDB Molecule:** Toll-like receptor 2<br>**PDBTitle:** crystal structure of the chicken toll-like receptor 15 tir domain (glutathione adduct)<br>**PDB Entry:** PDBe RCSB PDBj
29	c7jlvD_	Alignment	not modelled	99.6	11	**PDB header:** immune system<br>**Chain:** D: **PDB Molecule:** Disease resistance protein Roq1<br>**PDBTitle:** structure of the activated roq1 resistosome directly recognizing the pathogen effector xopq<br>**PDB Entry:** PDBe RCSB PDBj
30	c4w8gA_	Alignment	not modelled	99.4	22	**PDB header:** signaling protein<br>**Chain:** A: **PDB Molecule:** Toll-receptor-related 2<br>**PDBTitle:** ;crystal structure of the tir domain of the toll-related receptor trr-2 from the lower metazoan hydra magnipapillata (crystal form i);<br>**PDB Entry:** PDBe RCSB PDBj
31	c7szlA_	Alignment	not modelled	99.2	21	**PDB header:** membrane protein<br>**Chain:** A: **PDB Molecule:** X-linked interleukin-1 receptor accessory protein-like 2<br>**PDBTitle:** crystal structure of the toll/interleukin-1 receptor domain of human il-1r10 (il-1rapl2)<br>**PDB Entry:** PDBe RCSB PDBj
32	c7nuxA_	Alignment	not modelled	99.5	19	**PDB header:** signaling protein<br>**Chain:** A: **PDB Molecule:** Toll-like receptor 1<br>**PDBTitle:** crystal structure of the tir domain of human tlr1 (crystallised without zn2+ ions)<br>**PDB Entry:** PDBe RCSB PDBj
33	c7jlxA_	Alignment	not modelled	99.1	17	**PDB header:** immune system<br>**Chain:** A: **PDB Molecule:** Disease resistance protein Roq1<br>**PDBTitle:** structure of the activated roq1 resistosome directly recognizing the pathogen effector xopq (tir domains)<br>**PDB Entry:** PDBe RCSB PDBj
34	c7jlxD_	Alignment	not modelled	99.1	17	**PDB header:** immune system<br>**Chain:** D: **PDB Molecule:** Disease resistance protein Roq1<br>**PDBTitle:** structure of the activated roq1 resistosome directly recognizing the pathogen effector xopq (tir domains)<br>**PDB Entry:** PDBe RCSB PDBj
35	c7nuwA_	Alignment	not modelled	99.4	19	**PDB header:** signaling protein<br>**Chain:** A: **PDB Molecule:** Toll-like receptor 1<br>**PDBTitle:** crystal structure of the tir domain of human tlr1 (crystallised with zn2+ ions)<br>**PDB Entry:** PDBe RCSB PDBj
36	c7fccA_	Alignment	not modelled	99.3	20	**PDB header:** immune system<br>**Chain:** A: **PDB Molecule:** Isoform 4 of Interleukin-1 receptor accessory protein<br>**PDBTitle:** il-1racpb tir domain<br>**PDB Entry:** PDBe RCSB PDBj
37	c6wt6A_	Alignment	not modelled	99.4	19	**PDB header:** immune system<br>**Chain:** A: **PDB Molecule:** Metazoan TIR-STING fusion<br>**PDBTitle:** structure of a metazoan tir-sting receptor from c. gigas<br>**PDB Entry:** PDBe RCSB PDBj
38	c3oziA_	Alignment	not modelled	99.2	16	**PDB header:** plant protein<br>**Chain:** A: **PDB Molecule:** L6tr<br>**PDBTitle:** crystal structure of the tir domain from the flax disease resistance protein l6<br>**PDB Entry:** PDBe RCSB PDBj
39	c9cc8A_	Alignment	not modelled	99.3	10	**PDB header:** plant protein<br>**Chain:** A: **PDB Molecule:** NLR-required for cell death 4<br>**PDBTitle:** hexameric state of the nrc4 resistosome<br>**PDB Entry:** PDBe RCSB PDBj
40	c7fchA_	Alignment	not modelled	99.4	20	**PDB header:** immune system<br>**Chain:** A: **PDB Molecule:** Interleukin-18 receptor accessory protein<br>**PDBTitle:** il-18rbeta tir domain<br>**PDB Entry:** PDBe RCSB PDBj
41	c7fchD_	Alignment	not modelled	99.4	20	**PDB header:** immune system<br>**Chain:** D: **PDB Molecule:** Interleukin-18 receptor accessory protein<br>**PDBTitle:** il-18rbeta tir domain<br>**PDB Entry:** PDBe RCSB PDBj
42	c6blbA_	Alignment	not modelled	97.3	12	**PDB header:** hydrolase<br>**Chain:** A: **PDB Molecule:** Holliday junction ATP-dependent DNA helicase RuvB<br>**PDBTitle:** ;1.88 angstrom resolution crystal structure holliday junction atp-dependent dna helicase (ruvb) from pseudomonas aeruginosa in complex with adp;<br>**PDB Entry:** PDBe RCSB PDBj
43	c3h16B_	Alignment	not modelled	99.0	20	**PDB header:** signaling protein<br>**Chain:** B: **PDB Molecule:** TIR protein<br>**PDBTitle:** crystal structure of a bacteria tir domain, pdtir from paracoccus denitrificans<br>**PDB Entry:** PDBe RCSB PDBj
44	c4om7A_	Alignment	not modelled	99.4	20	**PDB header:** signaling protein<br>**Chain:** A: **PDB Molecule:** Toll-like receptor 6<br>**PDBTitle:** crystal structure of tir domain of tlr6<br>**PDB Entry:** PDBe RCSB PDBj

45	c4c6tB_	Alignment	not modelled	99.0	16	**PDB header:** immune system<br>**Chain:** B: **PDB Molecule:** DISEASE RESISTANCE PROTEIN RPS4<br>**PDBTitle:** crystal structure of the rps4 and rrs1 tir domain heterodimer<br>**PDB Entry:** PDBe RCSB PDBj
46	c3ub3A_	Alignment	not modelled	99.3	21	**PDB header:** immune system<br>**Chain:** A: **PDB Molecule:** Toll/interleukin-1 receptor domain-containing adapter protein<br>**PDBTitle:** d96n variant of tir domain of mal/tirap<br>**PDB Entry:** PDBe RCSB PDBj
47	c5tecA_	Alignment	not modelled	97.9	17	**PDB header:** immune system<br>**Chain:** A: **PDB Molecule:** Protein SUPPRESSOR OF npr1-1, CONSTITUTIVE 1<br>**PDBTitle:** crystal structure of the tir domain from the arabidopsis thaliana nlr protein snc1<br>**PDB Entry:** PDBe RCSB PDBj
48	c4c6rD_	Alignment	not modelled	98.9	16	**PDB header:** immune system<br>**Chain:** D: **PDB Molecule:** DISEASE RESISTANCE PROTEIN RPS4<br>**PDBTitle:** crystal structure of the tir domain from the arabidopsis thaliana disease resistance protein rps4<br>**PDB Entry:** PDBe RCSB PDBj
49	c2chvD_	Alignment	not modelled	96.0	11	**PDB header:** dna binding protein<br>**Chain:** D: **PDB Molecule:** REPLICATION FACTOR C SMALL SUBUNIT<br>**PDBTitle:** replication factor c adpnp complex<br>**PDB Entry:** PDBe RCSB PDBj
50	c2chvA_	Alignment	not modelled	95.9	11	**PDB header:** dna binding protein<br>**Chain:** A: **PDB Molecule:** REPLICATION FACTOR C SMALL SUBUNIT<br>**PDBTitle:** replication factor c adpnp complex<br>**PDB Entry:** PDBe RCSB PDBj
51	c4lzpB_	Alignment	not modelled	98.9	25	**PDB header:** immune system<br>**Chain:** B: **PDB Molecule:** Putative uncharacterized protein<br>**PDBTitle:** structure of the tir domain of the immunosuppressor btpa from brucella<br>**PDB Entry:** PDBe RCSB PDBj
52	c4eo7A_	Alignment	not modelled	99.5	18	**PDB header:** protein binding<br>**Chain:** A: **PDB Molecule:** Myeloid differentiation primary response protein MyD88<br>**PDBTitle:** crystal structure of the tir domain of human myeloid differentiation primary response protein 88.<br>**PDB Entry:** PDBe RCSB PDBj
53	c1in8A_	Alignment	not modelled	97.4	11	**PDB header:** dna binding protein<br>**Chain:** A: **PDB Molecule:** HOLLIDAY JUNCTION DNA HELICASE RUVB<br>**PDBTitle:** thermotoga maritima ruvb t158v<br>**PDB Entry:** PDBe RCSB PDBj
54	c7fcjA_	Alignment	not modelled	99.3	18	**PDB header:** immune system<br>**Chain:** A: **PDB Molecule:** SIGIRR protein<br>**PDBTitle:** zebrafish sigirr tir domain mutant - c299s<br>**PDB Entry:** PDBe RCSB PDBj
55	c8jo0G_	Alignment	not modelled	99.3	10	**PDB header:** apoptosis<br>**Chain:** G: **PDB Molecule:** Cell death protein 4<br>**PDBTitle:** the cryo-em structure of a heptameric ced-4/ced-3 catalytic complex<br>**PDB Entry:** PDBe RCSB PDBj
56	c4domA_	Alignment	not modelled	99.5	18	**PDB header:** signaling protein<br>**Chain:** A: **PDB Molecule:** Myeloid differentiation primary response protein MyD88<br>**PDBTitle:** crystal structure of the tir-domain of human myeloid differentiation primary response protein (myd88)<br>**PDB Entry:** PDBe RCSB PDBj
57	c2j67A_	Alignment	not modelled	99.3	18	**PDB header:** receptor<br>**Chain:** A: **PDB Molecule:** TOLL LIKE RECEPTOR 10<br>**PDBTitle:** the tir domain of human toll-like receptor 10 (tlr10)<br>**PDB Entry:** PDBe RCSB PDBj
58	c3jrnA_	Alignment	not modelled	98.1	16	**PDB header:** plant protein<br>**Chain:** A: **PDB Molecule:** AT1G72930 protein<br>**PDBTitle:** crystal structure of tir domain from arabidopsis thaliana<br>**PDB Entry:** PDBe RCSB PDBj
59	c4lqcB_	Alignment	not modelled	98.8	25	**PDB header:** immune system<br>**Chain:** B: **PDB Molecule:** TcpB<br>**PDBTitle:** ;the crystal structures of the brucella protein tcpb and the tlr adaptor protein tirap show structural differences in microbial tir mimicry.;<br>**PDB Entry:** PDBe RCSB PDBj
60	c7tjkA_	Alignment	not modelled	97.1	9	**PDB header:** replication/dna<br>**Chain:** A: **PDB Molecule:** Origin recognition complex subunit 1<br>**PDBTitle:** s. cerevisiae orc bound to 84 bp ars1 dna and cdc6 (state 2) with docked orc6 n-terminal domain<br>**PDB Entry:** PDBe RCSB PDBj
61	c1fyxA_	Alignment	not modelled	99.4	17	**PDB header:** signaling protein<br>**Chain:** A: **PDB Molecule:** TOLL-LIKE RECEPTOR 2<br>**PDBTitle:** crystal structure of p681h mutant of tir domain of human tlr2<br>**PDB Entry:** PDBe RCSB PDBj
62	c8rfhA_	Alignment	not modelled	99.4	9	**PDB header:** immune system<br>**Chain:** A: **PDB Molecule:** NRC2a<br>**PDBTitle:** cryoem structure of the plant helper nlr nrc2 in its resting state<br>**PDB Entry:** PDBe RCSB PDBj
63	c1t3gA_	Alignment	not modelled	99.2	18	**PDB header:** membrane protein<br>**Chain:** A: **PDB Molecule:** X-linked interleukin-1 receptor accessory protein-like 1<br>**PDBTitle:** crystal structure of the toll/interleukin-1 receptor (tir) domain of human il-1rapl<br>**PDB Entry:** PDBe RCSB PDBj
64	c3pfiA_	Alignment	not modelled	94.9	11	**PDB header:** hydrolase<br>**Chain:** A: **PDB Molecule:** Holliday junction ATP-dependent DNA helicase ruvB<br>**PDBTitle:** ;2.7 angstrom resolution crystal structure of a probable holliday junction dna helicase (ruvb) from campylobacter jejuni subsp. jejuni nctc 11168 in complex with adenosine-5-diphosphate;<br>**PDB Entry:** PDBe RCSB PDBj

65	c8xuoA_	Alignment	not modelled	99.2	9	**PDB header:** plant protein<br>**Chain:** A: **PDB Molecule:** NRC2<br>**PDBTitle:** cryo-em structure of tomato nrc2 dimer<br>**PDB Entry:** PDBe RCSB PDBj
66	c2m1wA_	Alignment	not modelled	99.8	17	**PDB header:** immune system<br>**Chain:** A: **PDB Molecule:** TIR domain-containing adapter molecule 2<br>**PDBTitle:** ticam-2 tir domain<br>**PDB Entry:** PDBe RCSB PDBj
67	c8bv0A_	Alignment	not modelled	99.3	9	**PDB header:** protein binding<br>**Chain:** A: **PDB Molecule:** NRC1<br>**PDBTitle:** ;binary complex between the nb-arc domain from the tomato immune receptor nrc1 and the spry domain-containing effector ss15 from the potato cyst nematode;<br>**PDB Entry:** PDBe RCSB PDBj
68	c7xozA_	Alignment	not modelled	98.9	14	**PDB header:** hydrolase<br>**Chain:** A: **PDB Molecule:** ADP-ribosyl cyclase/cyclic ADP-ribose hydrolase<br>**PDBTitle:** crystal structure of rppt-tir<br>**PDB Entry:** PDBe RCSB PDBj
69	c7xozB_	Alignment	not modelled	98.9	14	**PDB header:** hydrolase<br>**Chain:** B: **PDB Molecule:** ADP-ribosyl cyclase/cyclic ADP-ribose hydrolase<br>**PDBTitle:** crystal structure of rppt-tir<br>**PDB Entry:** PDBe RCSB PDBj
70	c5tebA_	Alignment	not modelled	98.9	14	**PDB header:** signaling protein<br>**Chain:** A: **PDB Molecule:** Recognition of Peronospora parasitica 1<br>**PDBTitle:** crystal structure of the tir domain from the arabidopsis thaliana disease resistance protein rpp1<br>**PDB Entry:** PDBe RCSB PDBj
71	c3sfzA_	Alignment	not modelled	99.3	9	**PDB header:** apoptosis<br>**Chain:** A: **PDB Molecule:** Apoptotic peptidase activating factor 1<br>**PDBTitle:** crystal structure of full-length murine apaf-1<br>**PDB Entry:** PDBe RCSB PDBj
72	c6j5tC_	Alignment	not modelled	99.2	9	**PDB header:** plant protein<br>**Chain:** C: **PDB Molecule:** Disease resistance RPP13-like protein 4<br>**PDBTitle:** reconstitution and structure of a plant nlr resistosome conferring immunity<br>**PDB Entry:** PDBe RCSB PDBj
73	c8umvE_	Alignment	not modelled	95.0	12	**PDB header:** replication/dna<br>**Chain:** E: **PDB Molecule:** Replication factor C subunit 3<br>**PDBTitle:** atomic model of the human ctf18-rfc-pcna-dna ternary complex with narrow pcna opening state i (state 5)<br>**PDB Entry:** PDBe RCSB PDBj
74	c8ffiA_	Alignment	not modelled	99.9	20	**PDB header:** immune system<br>**Chain:** A: **PDB Molecule:** TIR-APAZ<br>**PDBTitle:** structure of tetramerized mapsparta upon guide rna-mediated target dna binding<br>**PDB Entry:** PDBe RCSB PDBj
75	c1iqpA_	Alignment	not modelled	96.5	11	**PDB header:** replication<br>**Chain:** A: **PDB Molecule:** RFCS<br>**PDBTitle:** crystal structure of the clamp loader small subunit from pyrococcus furiosus<br>**PDB Entry:** PDBe RCSB PDBj
76	c4l15A_	Alignment	not modelled	93.6	9	**PDB header:** hydrolase<br>**Chain:** A: **PDB Molecule:** Fidgetin-like protein 1<br>**PDBTitle:** crystal structure of figl-1 aaa domain<br>**PDB Entry:** PDBe RCSB PDBj
77	c8xvbB_	Alignment	not modelled	97.0	12	**PDB header:** viral protein/dna<br>**Chain:** B: **PDB Molecule:** ATP-dependent target DNA activator B<br>**PDBTitle:** cryo-em structure of atp-dna-mub filaments<br>**PDB Entry:** PDBe RCSB PDBj
78	c2a5yB_	Alignment	not modelled	99.3	9	**PDB header:** apoptosis<br>**Chain:** B: **PDB Molecule:** ced-4<br>**PDBTitle:** structure of a ced-4/ced-9 complex<br>**PDB Entry:** PDBe RCSB PDBj
79	c8xvbD_	Alignment	not modelled	96.8	12	**PDB header:** viral protein/dna<br>**Chain:** D: **PDB Molecule:** ATP-dependent target DNA activator B<br>**PDBTitle:** cryo-em structure of atp-dna-mub filaments<br>**PDB Entry:** PDBe RCSB PDBj
80	c2chgA_	Alignment	not modelled	93.6	12	**PDB header:** dna binding protein<br>**Chain:** A: **PDB Molecule:** REPLICATION FACTOR C SMALL SUBUNIT<br>**PDBTitle:** replication factor c domains 1 and 2<br>**PDB Entry:** PDBe RCSB PDBj
81	c8efvC_	Alignment	not modelled	97.5	10	**PDB header:** structural protein<br>**Chain:** C: **PDB Molecule:** Holliday junction ATP-dependent DNA helicase RuvB<br>**PDBTitle:** structure of single homo-hexameric holliday junction atp-dependent dna helicase ruvb motor<br>**PDB Entry:** PDBe RCSB PDBj
82	c6vvoC_	Alignment	not modelled	96.5	10	**PDB header:** dna binding protein, replication<br>**Chain:** C: **PDB Molecule:** Replication factor C subunit 5<br>**PDBTitle:** ;structure of the human clamp loader (replication factor c, rfc) bound to the sliding clamp (proliferating cell nuclear antigen, pcna);<br>**PDB Entry:** PDBe RCSB PDBj
83	c5y8fA_	Alignment	not modelled	99.4	16	**PDB header:** signaling protein<br>**Chain:** A: **PDB Molecule:** Sefir domain protein<br>**PDBTitle:** crystal structure of a prokaryotic sefir domain<br>**PDB Entry:** PDBe RCSB PDBj
84	c8umyE_	Alignment	not modelled	90.7	11	**PDB header:** replication/dna<br>**Chain:** E: **PDB Molecule:** Replication factor C subunit 3<br>**PDBTitle:** atomic model of the human ctf18-rfc-pcna-dna ternary complex with narrow pcna opening state ii (state 6)<br>**PDB Entry:** PDBe RCSB PDBj
85	c7xc2G_	Alignment	not modelled	99.0	8	**PDB header:** plant protein/inhibitor<br>**Chain:** G: **PDB Molecule:** CNL9<br>**PDBTitle:** cryo em structure of oligomeric complex formed by wheat cnl sr35 and the effector avrsr35 of the wheat stem rust pathogen<br>**PDB Entry:** PDBe RCSB PDBj

86	c7pboD_	Alignment	not modelled	96.2	9	**PDB header:** hydrolase<br>**Chain:** D: **PDB Molecule:** Holliday junction ATP-dependent DNA helicase RuvB<br>**PDBTitle:** ruvab branch migration motor complexed to the holliday junction - ruvb aaa+ state s4 [t2 dataset]<br>**PDB Entry:** PDBe RCSB PDBj
87	c7xc2A_	Alignment	not modelled	99.0	8	**PDB header:** plant protein/inhibitor<br>**Chain:** A: **PDB Molecule:** CNL9<br>**PDBTitle:** cryo em structure of oligomeric complex formed by wheat cnl sr35 and the effector avrsr35 of the wheat stem rust pathogen<br>**PDB Entry:** PDBe RCSB PDBj
88	c7jpoA_	Alignment	not modelled	98.3	8	**PDB header:** replication<br>**Chain:** A: **PDB Molecule:** Origin recognition complex subunit 1<br>**PDBTitle:** orc-o1aaa: human origin recognition complex (orc) with dynamic/unresolved orc2 wh<br>**PDB Entry:** PDBe RCSB PDBj
89	c9emcB_	Alignment	not modelled	94.6	9	**PDB header:** chaperone<br>**Chain:** B: **PDB Molecule:** RuvB-like 2<br>**PDBTitle:** ruvbl1/2 in complex with atp<br>**PDB Entry:** PDBe RCSB PDBj
90	c7tjkI_	Alignment	not modelled	97.1	8	**PDB header:** replication/dna<br>**Chain:** I: **PDB Molecule:** Cell division control protein 6<br>**PDBTitle:** s. cerevisiae orc bound to 84 bp ars1 dna and cdc6 (state 2) with docked orc6 n-terminal domain<br>**PDB Entry:** PDBe RCSB PDBj
91	c8dr1A_	Alignment	not modelled	90.9	13	**PDB header:** replication/dna<br>**Chain:** A: **PDB Molecule:** Replication factor C subunit 1<br>**PDBTitle:** ;consensus closed state of rfc:pcna bound to a 3 ss/dsdna junction (dna2);<br>**PDB Entry:** PDBe RCSB PDBj
92	c2fnaA_	Alignment	not modelled	97.2	11	**PDB header:** atp-binding protein<br>**Chain:** A: **PDB Molecule:** Conserved hypothetical protein<br>**PDBTitle:** crystal structure of an archaeal aaa+ atpase (sso1545) from sulfolobus solfataricus p2 at 2.00 a resolution<br>**PDB Entry:** PDBe RCSB PDBj
93	c2qbyC_	Alignment	not modelled	97.6	8	**PDB header:** replication/dna<br>**Chain:** C: **PDB Molecule:** Cell division control protein 6 homolog 1<br>**PDBTitle:** crystal structure of a heterodimer of cdc6/orc1 initiators bound to origin dna (from s. solfataricus)<br>**PDB Entry:** PDBe RCSB PDBj
94	c4mejC_	Alignment	not modelled	85.8	16	**PDB header:** transferase/transferase inhibitor<br>**Chain:** C: **PDB Molecule:** Nucleoside deoxyribosyltransferase<br>**PDBTitle:** ;crystal structure of lactobacillus helveticus purine deoxyribosyl transferase (pdt) with the tricyclic purine 8,9-dihydro-9-oxoimidazo[2,1-b]purine (n2,3-ethenoguanine);<br>**PDB Entry:** PDBe RCSB PDBj
95	c7wbtA_	Alignment	not modelled	97.0	9	**PDB header:** immune system<br>**Chain:** A: **PDB Molecule:** NACHT, LRR and PYD domains-containing protein 9<br>**PDBTitle:** crystal structure of bovine nlrp9<br>**PDB Entry:** PDBe RCSB PDBj
96	c8umvC_	Alignment	not modelled	96.5	9	**PDB header:** replication/dna<br>**Chain:** C: **PDB Molecule:** Replication factor C subunit 5<br>**PDBTitle:** atomic model of the human ctf18-rfc-pcna-dna ternary complex with narrow pcna opening state i (state 5)<br>**PDB Entry:** PDBe RCSB PDBj
97	c8unjC_	Alignment	not modelled	96.5	9	**PDB header:** replication<br>**Chain:** C: **PDB Molecule:** Replication factor C subunit 5<br>**PDBTitle:** atomic model of the human ctf18-rfc alone in the apo state (state 1)<br>**PDB Entry:** PDBe RCSB PDBj
98	c3vbcA_	Alignment	not modelled	97.2	16	**PDB header:** immune system<br>**Chain:** A: **PDB Molecule:** Interleukin-17 receptor B<br>**PDBTitle:** crystal structure of il-17 receptor b sefir domain<br>**PDB Entry:** PDBe RCSB PDBj
99	c8dr1E_	Alignment	not modelled	95.3	10	**PDB header:** replication/dna<br>**Chain:** E: **PDB Molecule:** Replication factor C subunit 5<br>**PDBTitle:** ;consensus closed state of rfc:pcna bound to a 3 ss/dsdna junction (dna2);<br>**PDB Entry:** PDBe RCSB PDBj
100	c1s2dB_	Alignment	not modelled	83.2	16	**PDB header:** transferase<br>**Chain:** B: **PDB Molecule:** Nucleoside 2-deoxyribosyltransferase<br>**PDBTitle:** ;purine 2-deoxyribosyl complex with arabinoside: ribosylated intermediate (araa);<br>**PDB Entry:** PDBe RCSB PDBj
101	c1s2lB_	Alignment	not modelled	83.2	16	**PDB header:** transferase<br>**Chain:** B: **PDB Molecule:** purine trans deoxyribosylase<br>**PDBTitle:** ;purine 2deoxyribosyltransferase native structure;<br>**PDB Entry:** PDBe RCSB PDBj
102	c8bobC_	Alignment	not modelled	98.0	9	**PDB header:** transcription<br>**Chain:** C: **PDB Molecule:** HTH-type transcriptional regulator MalT<br>**PDBTitle:** structural basis for negative regulation of the maltose system<br>**PDB Entry:** PDBe RCSB PDBj
103	c1w5tB_	Alignment	not modelled	97.7	8	**PDB header:** dna replication initiation<br>**Chain:** B: **PDB Molecule:** ORC2<br>**PDBTitle:** structure of the aeropyrum pernix orc2 protein (adpnp-adp complexes)<br>**PDB Entry:** PDBe RCSB PDBj
104	c8c0vA_	Alignment	not modelled	95.5	10	**PDB header:** translocase<br>**Chain:** A: **PDB Molecule:** Peroxisomal ATPase PEX1<br>**PDBTitle:** structure of the peroxisomal pex1/pex6 atpase complex bound to a substrate in single seam state<br>**PDB Entry:** PDBe RCSB PDBj
105	c7qqkD_	Alignment	not modelled	99.1	18	**PDB header:** signaling protein<br>**Chain:** D: **PDB Molecule:** TIR_SAVED fusion protein<br>**PDBTitle:** tir-saved effector bound to ca3<br>**PDB Entry:** PDBe RCSB PDBj

106	c8darB_	Alignment	not modelled	94.5	9	**PDB header:** motor protein<br>**Chain:** B: **PDB Molecule:** Cell division control protein 48<br>**PDBTitle:** saccharomyces cerevisiae ufd1/npl4/cdc48 complex unbound but in the presence of sumo-ubiquitin(k48polyub)-meos and atp<br>**PDB Entry:** PDBe RCSB PDBj
107	c7jppE_	Alignment	not modelled	91.5	9	**PDB header:** replication<br>**Chain:** E: **PDB Molecule:** Origin recognition complex subunit 5<br>**PDBTitle:** orc-o2wh: human origin recognition complex (orc) with dynamic/unresolved orc1 aaa+ domain<br>**PDB Entry:** PDBe RCSB PDBj
108	c6ugeE_	Alignment	not modelled	92.7	9	**PDB header:** motor protein<br>**Chain:** E: **PDB Molecule:** Meiotic spindle formation protein mei-1<br>**PDBTitle:** katanin hexamer in the ring conformation in complex with substrate<br>**PDB Entry:** PDBe RCSB PDBj
109	c8btnB_	Alignment	not modelled	99.4	15	**PDB header:** hydrolase<br>**Chain:** B: **PDB Molecule:** TIR domain-containing protein<br>**PDBTitle:** crystal structure of bcthsb<br>**PDB Entry:** PDBe RCSB PDBj
110	c7jpoE_	Alignment	not modelled	90.4	9	**PDB header:** replication<br>**Chain:** E: **PDB Molecule:** Origin recognition complex subunit 5<br>**PDBTitle:** orc-o1aaa: human origin recognition complex (orc) with dynamic/unresolved orc2 wh<br>**PDB Entry:** PDBe RCSB PDBj
111	c7w46B_	Alignment	not modelled	94.3	13	**PDB header:** hydrolase<br>**Chain:** B: **PDB Molecule:** Uncharacterized ATPase YjoB<br>**PDBTitle:** crystal structure of bacillus subtilis yjob with adp<br>**PDB Entry:** PDBe RCSB PDBj
112	c7mhbA_	Alignment	not modelled	94.2	9	**PDB header:** chaperone<br>**Chain:** A: **PDB Molecule:** ATP-dependent protease ATPase subunit HslU<br>**PDBTitle:** ;crystal structure of atp-dependent protease atpase subunit hslu in complex with adenosine 5-diphosphate;<br>**PDB Entry:** PDBe RCSB PDBj
113	c8rwvF_	Alignment	not modelled	97.4	7	**PDB header:** replication<br>**Chain:** F: **PDB Molecule:** Origin recognition complex subunit 1<br>**PDBTitle:** human occm dna licensing intermediate<br>**PDB Entry:** PDBe RCSB PDBj
114	c7jk2D_	Alignment	not modelled	94.6	8	**PDB header:** replication/dna<br>**Chain:** D: **PDB Molecule:** Origin recognition complex subunit 5<br>**PDBTitle:** structure of drosophila orc bound to poly(da/dt) dna and cdc6 (conformation 1)<br>**PDB Entry:** PDBe RCSB PDBj
115	c4nuxA_	Alignment	not modelled	98.2	12	**PDB header:** immune system<br>**Chain:** A: **PDB Molecule:** Interleukin-17 receptor A<br>**PDBTitle:** structure of receptor a<br>**PDB Entry:** PDBe RCSB PDBj
116	c8dr1B_	Alignment	not modelled	97.1	8	**PDB header:** replication/dna<br>**Chain:** B: **PDB Molecule:** Replication factor C subunit 4<br>**PDBTitle:** ;consensus closed state of rfc:pcna bound to a 3 ss/dsdna junction (dna2);<br>**PDB Entry:** PDBe RCSB PDBj
117	c3glhB_	Alignment	not modelled	94.1	8	**PDB header:** transferase<br>**Chain:** B: **PDB Molecule:** DNA polymerase III subunit tau<br>**PDBTitle:** crystal structure of the e. coli clamp loader bound to psi peptide<br>**PDB Entry:** PDBe RCSB PDBj
118	c5vhnB_	Alignment	not modelled	91.0	8	**PDB header:** hydrolase<br>**Chain:** B: **PDB Molecule:** 26S proteasome regulatory subunit 7<br>**PDBTitle:** conformational landscape of the p28-bound human proteasome regulatory particle<br>**PDB Entry:** PDBe RCSB PDBj
119	c1fnnA_	Alignment	not modelled	98.0	7	**PDB header:** cell cycle<br>**Chain:** A: **PDB Molecule:** CELL DIVISION CONTROL PROTEIN 6<br>**PDBTitle:** crystal structure of cdc6p from pyrobaculum aerophilum<br>**PDB Entry:** PDBe RCSB PDBj
120	c6h7xA_	Alignment	not modelled	94.1	9	**PDB header:** chaperone<br>**Chain:** A: **PDB Molecule:** RuvB-like 2<br>**PDBTitle:** first x-ray structure of full-length human ruvb-like 2.<br>**PDB Entry:** PDBe RCSB PDBj
