# Supplementary material for: From CRISPR–Cas to Argonautes: Defense systems of bacteria and archaea in the hydrothermal deep subsurface
Source: ISME Commun. 2026 Jul 10;6(1):ycag200. doi: 10.1093/ismeco/ycag200 (PMC13477220; doi:10.1093/ismeco/ycag200)
Supplement: Supplementary file 1 [file supplementary_material_ycag200.zip › Supplementary_Materials_ycag200.zip!/_12_bin_10_fna-01588_hypothetical_protein.pdf]

Phyre2.2

Email paraskevi.mara@gmail.com
Description _12_bin_10_fna-_01588_hypothetical_protein
Date Mon Dec 22 19:13:12 GMT 2025
Unique Job ID 337aafc757b4407b

Detailed template information

#	Template	Alignment Coverage	3D Model	Confidence	% i.d.	Template Information
1	c8u72C_	Alignment	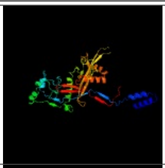	100.0	16	**PDB header:** rna binding protein/rna/dna<br>**Chain:** C: **PDB Molecule:** TIR domain-containing protein<br>**PDBTitle:** cryo-em structure of the sparta oligomer with guide rna and target dna<br>**PDB Entry:** PDBe RCSB PDBj
2	c3lmmD_	Alignment	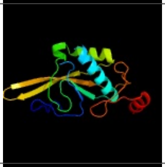	98.6	19	**PDB header:** structural genomics, unknown function<br>**Chain:** D: **PDB Molecule:** Uncharacterized protein<br>**PDBTitle:** crystal structure of the dip2311 protein from corynebacterium diphtheriae, northeast structural genomics consortium target cdr35<br>**PDB Entry:** PDBe RCSB PDBj
3	c8ozeC_	Alignment	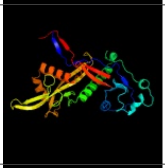	100.0	18	**PDB header:** immune system<br>**Chain:** C: **PDB Molecule:** TIR domain-containing protein<br>**PDBTitle:** cryoem structure of sparta complex dimer high resolution<br>**PDB Entry:** PDBe RCSB PDBj
4	c8isyB_	Alignment	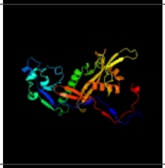	100.0	16	**PDB header:** dna binding protein<br>**Chain:** B: **PDB Molecule:** TIR domain-containing protein<br>**PDBTitle:** cryo-em structure of free-state crt-sparta<br>**PDB Entry:** PDBe RCSB PDBj
5	c8ffiA_	Alignment	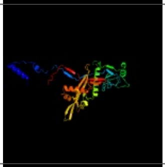	100.0	14	**PDB header:** immune system<br>**Chain:** A: **PDB Molecule:** TIR-APAZ<br>**PDBTitle:** structure of tetramerized mapsparta upon guide rna-mediated target dna binding<br>**PDB Entry:** PDBe RCSB PDBj
6	c8ffiM_	Alignment	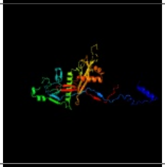	100.0	14	**PDB header:** immune system<br>**Chain:** M: **PDB Molecule:** TIR-APAZ<br>**PDBTitle:** structure of tetramerized mapsparta upon guide rna-mediated target dna binding<br>**PDB Entry:** PDBe RCSB PDBj
7	c8k88D_	Alignment	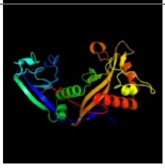	99.2	13	**PDB header:** dna/rna/cell invasion<br>**Chain:** D: **PDB Molecule:** Sir2 superfamily protein<br>**PDBTitle:** structure of procaryotic ago<br>**PDB Entry:** PDBe RCSB PDBj
8	c8in8A_	Alignment	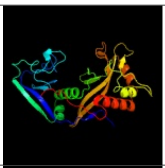	97.8	13	**PDB header:** antiviral protein<br>**Chain:** A: **PDB Molecule:** Sir2 superfamily protein<br>**PDBTitle:** cryo-em structure of the target ssdna-bound sir2-apaz/ago-grna quaternary complex<br>**PDB Entry:** PDBe RCSB PDBj
9	c2kyyA_	Alignment	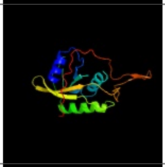	98.9	16	**PDB header:** hydrolase<br>**Chain:** A: **PDB Molecule:** Possible ATP-dependent DNA helicase RecG-related protein<br>**PDBTitle:** ;solution nmr structure of the n-terminal domain of putative atp-dependent dna helicase recg-related protein from nitrosomonas europaea, northeast structural genomics consortium target ner70a;<br>**PDB Entry:** PDBe RCSB PDBj
10	c5yd0B_	Alignment	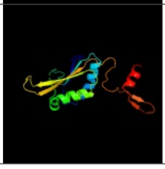	98.4	16	**PDB header:** hydrolase<br>**Chain:** B: **PDB Molecule:** Schlafen 8<br>**PDBTitle:** ;crystal structure of schlafen 13 (slfn13) n-domain;<br>**PDB Entry:** PDBe RCSB PDBj

11	c7q3zA_	Alignment	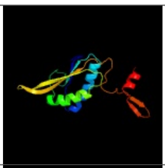	98.4	15	**PDB header:** dna binding protein<br>**Chain:** A: **PDB Molecule:** Schlafen family member 5<br>**PDBTitle:** dna/rna binding protein<br>**PDB Entry:** PDBe RCSB PDBj
12	c6rr9A_	Alignment	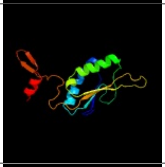	98.3	15	**PDB header:** dna binding protein<br>**Chain:** A: **PDB Molecule:** Schlafen family member 5<br>**PDBTitle:** dna/rna binding protein<br>**PDB Entry:** PDBe RCSB PDBj
13	c7lreA_	Alignment	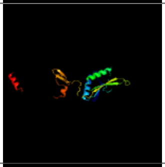	98.2	13	**PDB header:** rna binding protein<br>**Chain:** A: **PDB Molecule:** Schlafen family member 12<br>**PDBTitle:** cryo-em of the slfn12-pde3a complex: slfn12 body refinement<br>**PDB Entry:** PDBe RCSB PDBj
14	c7zelA_	Alignment	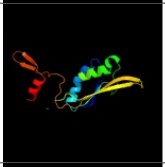	98.1	14	**PDB header:** hydrolase<br>**Chain:** A: **PDB Molecule:** Schlafen family member 11<br>**PDBTitle:** human slfn11 dimer apoenzyme<br>**PDB Entry:** PDBe RCSB PDBj
15	c9erfA_	Alignment	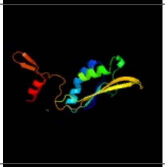	98.1	14	**PDB header:** hydrolase<br>**Chain:** A: **PDB Molecule:** Schlafen family member 11<br>**PDBTitle:** slfn11 dimer bound to trna-met-cat<br>**PDB Entry:** PDBe RCSB PDBj
16	c7fexA_	Alignment	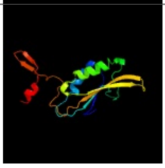	98.4	11	**PDB header:** antiviral protein<br>**Chain:** A: **PDB Molecule:** SLFN11<br>**PDBTitle:** crystal structure of sus scrofa schlafen11 n-terminal domain (2.69 a)<br>**PDB Entry:** PDBe RCSB PDBj
17	c4kr1A_	Alignment	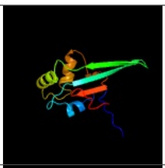	26.4	15	**PDB header:** cell cycle<br>**Chain:** A: **PDB Molecule:** Central kinetochore subunit IML3<br>**PDBTitle:** crystal structure of the kinetochore protein iml3 from budding yeast<br>**PDB Entry:** PDBe RCSB PDBj
18	c4d71A_	Alignment	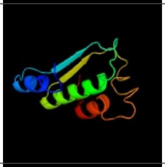	10.4	12	**PDB header:** hydrolase<br>**Chain:** A: **PDB Molecule:** GLYCOSIDE HYDROLASE<br>**PDBTitle:** ;crystal structure of a family 98 glycoside hydrolase catalytic module (sp3gh98) in complex with the type 2 blood group a-tetrasaccharide (e558a x02 mutant);<br>**PDB Entry:** PDBe RCSB PDBj
19	c2hqbA_	Alignment	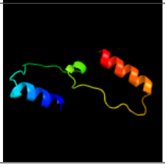	10.4	15	**PDB header:** transcription<br>**Chain:** A: **PDB Molecule:** Transcriptional activator of comK gene<br>**PDBTitle:** crystal structure of a transcriptional activator of comk gene from bacillus halodurans<br>**PDB Entry:** PDBe RCSB PDBj
