# Supplementary material for: From CRISPR–Cas to Argonautes: Defense systems of bacteria and archaea in the hydrothermal deep subsurface
Source: ISME Commun. 2026 Jul 10;6(1):ycag200. doi: 10.1093/ismeco/ycag200 (PMC13477220; doi:10.1093/ismeco/ycag200)
Supplement: Supplementary file 1 [file supplementary_material_ycag200.zip › Supplementary_Materials_ycag200.zip!/_SRR22580798_bin_19_fna-.pdf]

# Phyre2.2

| | |
|---|---|
| Email | paraskevi.mara@gmail.com |
| Description | _SRR22580798_bin_19_fna-_00701_hypothetical_protein_Aenigmatarchaeota |
| Date | Mon Dec 22 19:14:22 GMT 2025 |
| Unique Job ID | 9134ba88dd197f59 |

Detailed template information

| # | Template | Alignment Coverage | 3D Model | Confidence | % i.d. | Template Information |
|---|---|---|---|---|---|---|
| 1 | c7r8iA_ | Alignment | 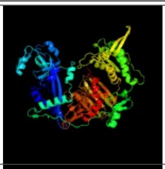 | 100.0 | 26 | **PDB header:** hydrolase/dna<br>**Chain:** A: **PDB Molecule:** Argonaute<br>**PDBTitle:** ;crystal structure of pseudooceanicola lipolyticus argonaute bound to 5 oh guide dna in the presence of mg2+;<br>**PDB Entry:** PDBe RCSB PDBj |
| 2 | c7r8gA_ | Alignment | 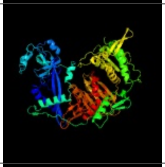 | 100.0 | 26 | **PDB header:** hydrolase/dna<br>**Chain:** A: **PDB Molecule:** Argonaute<br>**PDBTitle:** ;crystal structure of pseudooceanicola lipolyticus argonaute bound to 5 oh guide dna;<br>**PDB Entry:** PDBe RCSB PDBj |
| 3 | c8xihB_ | Alignment | 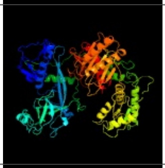 | 100.0 | 24 | **PDB header:** dna binding protein/dna<br>**Chain:** B: **PDB Molecule:** Piwi domain-containing protein<br>**PDBTitle:** protein-dna complex<br>**PDB Entry:** PDBe RCSB PDBj |
| 4 | c7svaA_ | Alignment | 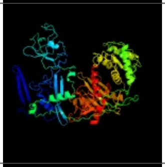 | 100.0 | 14 | **PDB header:** gene regulation<br>**Chain:** A: **PDB Molecule:** Protein argonaute 10<br>**PDBTitle:** cryo-em structure of arabidopsis ago10-guide rna complex<br>**PDB Entry:** PDBe RCSB PDBj |
| 5 | c6nitA_ | Alignment | 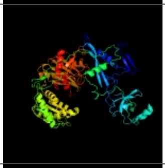 | 100.0 | 13 | **PDB header:** rna binding protein/rna<br>**Chain:** A: **PDB Molecule:** Protein argonaute-2<br>**PDBTitle:** human argonaute2-mir-122 bound to a target rna with four central mismatches (bu4)<br>**PDB Entry:** PDBe RCSB PDBj |
| 6 | c1yvuA_ | Alignment | 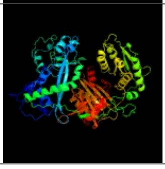 | 100.0 | 14 | **PDB header:** rna binding protein, gene regulation<br>**Chain:** A: **PDB Molecule:** hypothetical protein aq_1447<br>**PDBTitle:** crystal structure of a. aeolicus argonaute<br>**PDB Entry:** PDBe RCSB PDBj |
| 7 | c8vajA_ | Alignment | 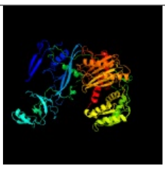 | 100.0 | 13 | **PDB header:** rna binding protein/rna<br>**Chain:** A: **PDB Molecule:** Protein argonaute-3<br>**PDBTitle:** human argonaute3 bound to cityrna and target rna<br>**PDB Entry:** PDBe RCSB PDBj |
| 8 | c4kreA_ | Alignment | 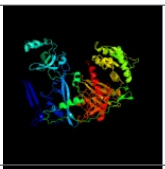 | 100.0 | 13 | **PDB header:** transcription/rna<br>**Chain:** A: **PDB Molecule:** Protein argonaute-1<br>**PDBTitle:** structure of human argonaute-1 bound to endogenous sf9 rna<br>**PDB Entry:** PDBe RCSB PDBj |
| 9 | c6mdzA_ | Alignment | 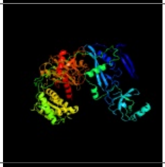 | 100.0 | 13 | **PDB header:** hydrolase/rna<br>**Chain:** A: **PDB Molecule:** Protein argonaute-2<br>**PDBTitle:** human argonaute2-mir-122 bound to a target rna with two central mismatches (bu2)<br>**PDB Entry:** PDBe RCSB PDBj |
| 10 | c5guhA_ | Alignment | 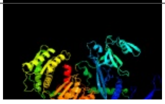 | 100.0 | 13 | **PDB header:** hydrolase/rna<br>**Chain:** A: **PDB Molecule:** PIWI<br>**PDBTitle:** crystal structure of silkworm piwi-clade argonaute siwi bound to [illegible] |

| | | | | | | |
|---|---|---|---|---|---|---|
| | | | 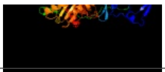 | | | pirna<br>**PDB Entry:** PDBe RCSB PDBj |
| 11 | c5vm9C_ | Alignment | 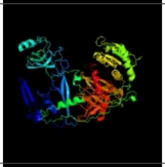 | 100.0 | 13 | **PDB header:** hydrolase/rna<br>**Chain:** C: **PDB Molecule:** Protein argonaute-3<br>**PDBTitle:** human argonaute3 bound to guide rna<br>**PDB Entry:** PDBe RCSB PDBj |
| 12 | c4w5tA_ | Alignment | 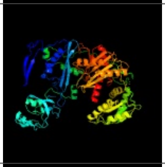 | 100.0 | 13 | **PDB header:** hydrolase/rna<br>**Chain:** A: **PDB Molecule:** Protein argonaute-2<br>**PDBTitle:** the crystal structure of human argonaute2 bound to a guide and target rna containing seed pairing from 2-7<br>**PDB Entry:** PDBe RCSB PDBj |
| 13 | c6n4oA_ | Alignment | 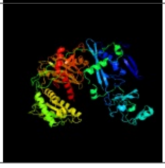 | 100.0 | 13 | **PDB header:** rna binding protein/rna<br>**Chain:** A: **PDB Molecule:** Protein argonaute-2<br>**PDBTitle:** human argonaute2-mir-122 bound to a seed and supplementary paired target<br>**PDB Entry:** PDBe RCSB PDBj |

| | | | | | | |
|---|---|---|---|---|---|---|
| 14 | c4z4dA_ | Alignment | 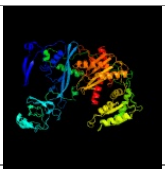 | 100.0 | 13 | **PDB header:** gene regulation/rna<br>**Chain:** A: **PDB Molecule:** Protein argonaute-2<br>**PDBTitle:** human argonaute2 bound to t1-g target rna<br>**PDB Entry:** PDBe RCSB PDBj |
| 15 | c6oonA_ | Alignment | 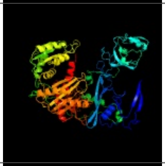 | 100.0 | 13 | **PDB header:** hydrolase/rna<br>**Chain:** A: **PDB Molecule:** Protein argonaute-4<br>**PDBTitle:** human argonaute4 bound to guide rna<br>**PDB Entry:** PDBe RCSB PDBj |
| 16 | c6kr6A_ | Alignment | 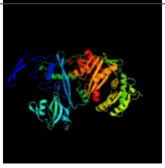 | 100.0 | 13 | **PDB header:** rna binding protein/rna<br>**Chain:** A: **PDB Molecule:** Protein piwi<br>**PDBTitle:** crystal structure of drosophila piwi<br>**PDB Entry:** PDBe RCSB PDBj |
| 17 | c2f8sC_ | Alignment | 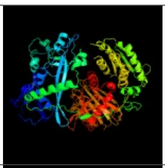 | 100.0 | 13 | **PDB header:** rna binding protein/rna<br>**Chain:** C: **PDB Molecule:** Argonaute protein<br>**PDBTitle:** crystal structure of aa-ago with externally-bound sirna<br>**PDB Entry:** PDBe RCSB PDBj |
| 18 | c4ncbA_ | Alignment | 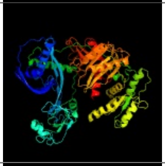 | 100.0 | 13 | **PDB header:** nuclear protein/dna<br>**Chain:** A: **PDB Molecule:** Argonaute<br>**PDBTitle:** structure of thermus thermophilus argonaute bound to guide dna and 19-mer target dna with mg2+<br>**PDB Entry:** PDBe RCSB PDBj |
| 19 | c7yfxA_ | Alignment | 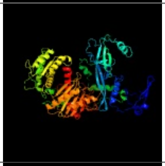 | 100.0 | 12 | **PDB header:** rna binding protein/rna<br>**Chain:** A: **PDB Molecule:** Piwi-like protein 2<br>**PDBTitle:** cryo-em structure of hili in complex with pirna<br>**PDB Entry:** PDBe RCSB PDBj |
| 20 | c8jpxA_ | Alignment | 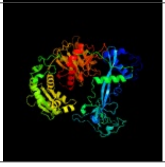 | 100.0 | 13 | **PDB header:** gene regulation<br>**Chain:** A: **PDB Molecule:** Protein argonaute<br>**PDBTitle:** cryo-em structure of pfago-guide dna-target dna complex<br>**PDB Entry:** PDBe RCSB PDBj |
| 21 | c8jpxD_ | Alignment | not modelled | 100.0 | 13 | **PDB header:** gene regulation<br>**Chain:** D: **PDB Molecule:** Protein argonaute<br>**PDBTitle:** cryo-em structure of pfago-guide dna-target dna complex<br>**PDB Entry:** PDBe RCSB PDBj |
| 22 | c7kx9A_ | Alignment | not modelled | 100.0 | 12 | **PDB header:** hydrolase,rna binding protein/rna<br>**Chain:** A: **PDB Molecule:** Piwi-A<br>**PDBTitle:** cryo-em structure of ephydatia fluviatilis piwia-pirna-target complex<br>**PDB Entry:** PDBe RCSB PDBj |
| 23 | c7yfqC_ | Alignment | not modelled | 100.0 | 12 | **PDB header:** rna binding protein/rna<br>**Chain:** C: **PDB Molecule:** Piwi<br>**PDBTitle:** cryo-em structure of the efpiwi (n959k)-pirna-target ternary [illegible] |

| | | | | | | |
|---|---|---|---|---|---|---|
| 23 | c7yiqC_ | Alignment | not modelled | 100.0 | 12 | **PDBTitle:** cryo-em structure of the efpiwi (h959k)-pirna-target ternary complex<br>**PDB Entry:** PDBe RCSB PDBj |
| 24 | c1u04A_ | Alignment | not modelled | 100.0 | 13 | **PDB header:** hydrolase/gene regulation<br>**Chain:** A: **PDB Molecule:** hypothetical protein PF0537<br>**PDBTitle:** crystal structure of full length argonaute from pyrococcus furiosus<br>**PDB Entry:** PDBe RCSB PDBj |
| 25 | c3dlbA_ | Alignment | not modelled | 100.0 | 13 | **PDB header:** nucleic acid binding protein/dna<br>**Chain:** A: **PDB Molecule:** argonaute<br>**PDBTitle:** crystal structure of the guide-strand-containing argonaute protein silencing complex<br>**PDB Entry:** PDBe RCSB PDBj |
| 26 | c5g5sA_ | Alignment | not modelled | 100.0 | 12 | **PDB header:** hydrolase<br>**Chain:** A: **PDB Molecule:** ARGONAUTE<br>**PDBTitle:** structure of the argonaute protein from methanocaldcoccus janaschii<br>**PDB Entry:** PDBe RCSB PDBj |
| 27 | c8wd8A_ | Alignment | not modelled | 100.0 | 12 | **PDB header:** dna binding protein/dna<br>**Chain:** A: **PDB Molecule:** Argonaute family protein<br>**PDBTitle:** cryo-em structure of ttdago-guide dna-target dna complex<br>**PDB Entry:** PDBe RCSB PDBj |
| 28 | c5i4aC_ | Alignment | not modelled | 100.0 | 13 | **PDB header:** rna binding protein/rna<br>**Chain:** C: **PDB Molecule:** Argonaute protein<br>**PDBTitle:** ;x-ray crystal structure of marinitoga piezophila argonaute in complex with 5 oh guide rna;<br>**PDB Entry:** PDBe RCSB PDBj |
| 29 | c6d92A_ | Alignment | not modelled | 100.0 | 11 | **PDB header:** rna binding protein<br>**Chain:** A: **PDB Molecule:** Uncharacterized protein<br>**PDBTitle:** ternary rsago complex with guide rna and target dna containing a-a non-canonical pair at position 3<br>**PDB Entry:** PDBe RCSB PDBj |
| 30 | c6d92D_ | Alignment | not modelled | 100.0 | 11 | **PDB header:** rna binding protein<br>**Chain:** D: **PDB Molecule:** Uncharacterized protein<br>**PDBTitle:** ternary rsago complex with guide rna and target dna containing a-a non-canonical pair at position 3<br>**PDB Entry:** PDBe RCSB PDBj |
| 31 | c8fexB_ | Alignment | not modelled | 100.0 | 14 | **PDB header:** immune system<br>**Chain:** B: **PDB Molecule:** short pAgo<br>**PDBTitle:** inactivate state of maribacter polysiphoniae argonuate (short pago system)<br>**PDB Entry:** PDBe RCSB PDBj |
| 32 | c8isyA_ | Alignment | not modelled | 100.0 | 14 | **PDB header:** dna binding protein<br>**Chain:** A: **PDB Molecule:** Piwi domain-containing protein<br>**PDBTitle:** cryo-em structure of free-state crt-sparta<br>**PDB Entry:** PDBe RCSB PDBj |
| 33 | c5theE_ | Alignment | not modelled | 100.0 | 13 | **PDB header:** rna binding protein/rna<br>**Chain:** E: **PDB Molecule:** Uncharacterized protein<br>**PDBTitle:** crystal structure of the c-terminal lobe of a budding yeast argonaute<br>**PDB Entry:** PDBe RCSB PDBj |
| 34 | c5theG_ | Alignment | not modelled | 100.0 | 13 | **PDB header:** rna binding protein/rna<br>**Chain:** G: **PDB Molecule:** Uncharacterized protein<br>**PDBTitle:** crystal structure of the c-terminal lobe of a budding yeast argonaute<br>**PDB Entry:** PDBe RCSB PDBj |
| 35 | c8ffiB_ | Alignment | not modelled | 100.0 | 14 | **PDB header:** immune system<br>**Chain:** B: **PDB Molecule:** short pAgo<br>**PDBTitle:** structure of tetramerized mapsparta upon guide rna-mediated target dna binding<br>**PDB Entry:** PDBe RCSB PDBj |

| | | | | | | |
|---|---|---|---|---|---|---|
| 36 | c8qg0A_ | Alignment | not modelled | 100.0 | 14 | **PDB header:** dna binding protein<br>**Chain:** A: **PDB Molecule:** Piwi protein<br>**PDBTitle:** archaeoglobus fulgidus afago complex with afago-n protein (fafago) bound with 17 nt rna guide and 17 nt dna target<br>**PDB Entry:** PDBe RCSB PDBj |
| 37 | c8pvvA_ | Alignment | not modelled | 100.0 | 14 | **PDB header:** dna binding protein<br>**Chain:** A: **PDB Molecule:** Piwi protein<br>**PDBTitle:** archaeoglobus fulgidus afago complex with afago-n protein (fafago) bound with 30 nt rna guide and 51 nt dna target<br>**PDB Entry:** PDBe RCSB PDBj |
| 38 | c6t5tA_ | Alignment | not modelled | 100.0 | 14 | **PDB header:** dna binding protein<br>**Chain:** A: **PDB Molecule:** Piwi protein AF_1318<br>**PDBTitle:** ;crystal structure of archaeoglobus fulgidus argonaute protein with cognate dna oligoduplex 5-pattgtggccacaat;<br>**PDB Entry:** PDBe RCSB PDBj |
| 39 | c8u72D_ | Alignment | not modelled | 100.0 | 13 | **PDB header:** rna binding protein/rna/dna<br>**Chain:** D: **PDB Molecule:** Piwi domain-containing protein<br>**PDBTitle:** cryo-em structure of the sparta oligomer with guide rna and target dna<br>**PDB Entry:** PDBe RCSB PDBj |
| 40 | c8k88C_ | Alignment | not modelled | 100.0 | 12 | **PDB header:** dna/rna/cell invasion<br>**Chain:** C: **PDB Molecule:** Piwi domain protein<br>**PDBTitle:** structure of procaryotic ago<br>**PDB Entry:** PDBe RCSB PDBj |
| 41 | c8jl0B_ | Alignment | not modelled | 100.0 | 11 | **PDB header:** antiviral protein/dna/rna<br>**Chain:** B: **PDB Molecule:** Piwi domain protein<br>**PDBTitle:** cryo-em structure of the prokaryotic sparsa system complex<br>**PDB Entry:** PDBe RCSB PDBj |
| 42 | c8in8B_ | Alignment | not modelled | 100.0 | 11 | **PDB header:** antiviral protein<br>**Chain:** B: **PDB Molecule:** Piwi domain protein<br>**PDBTitle:** cryo-em structure of the target ssdna-bound sir2-apaz/ago-grna quaternary complex<br>**PDB Entry:** PDBe RCSB PDBj |
| 43 | c9ij2A_ | Alignment | not modelled | 100.0 | 13 | **PDB header:** rna binding protein/rna<br>**Chain:** A: **PDB Molecule:** Piwi-like protein 2<br>**PDBTitle:** cryo-em structure of mili-pirna-target (22-nt, comma)<br>**PDB Entry:** PDBe RCSB PDBj |

| | | | | | | |
|---|---|---|---|---|---|---|
| 44 | c8ifkB_ | Alignment | not modelled | 100.0 | 11 | **PDB header:** rna binding protein/rna/dna<br>**Chain:** B: **PDB Molecule:** Piwi domain-containing protein<br>**PDBTitle:** cryo-em structure of monomeric sparta grna-ssdna target complex<br>**PDB Entry:** PDBe RCSB PDBj |
| 45 | c9iizA_ | Alignment | not modelled | 100.0 | 11 | **PDB header:** rna binding protein/rna<br>**Chain:** A: **PDB Molecule:** Piwi<br>**PDBTitle:** cryo-em structure of efpiwi-pirna-target (25-nt, comma)<br>**PDB Entry:** PDBe RCSB PDBj |
| 46 | c2yhaA_ | Alignment | not modelled | 100.0 | 12 | **PDB header:** rna binding protein<br>**Chain:** A: **PDB Molecule:** POST-TRANSCRIPTIONAL GENE SILENCING PROTEIN QDE-2<br>**PDBTitle:** crystal structure of the n. crassa qde-2 ago mid-piwi domains<br>**PDB Entry:** PDBe RCSB PDBj |
| 47 | c2l5cA_ | Alignment | not modelled | 99.1 | 19 | **PDB header:** rna binding protein<br>**Chain:** A: **PDB Molecule:** Piwi-like protein 1<br>**PDBTitle:** solution structures of human piwi-like 1 paz domain<br>**PDB Entry:** PDBe RCSB PDBj |
| 48 | c3vnbA_ | Alignment | not modelled | 99.4 | 13 | **PDB header:** rna binding protein<br>**Chain:** A: **PDB Molecule:** Protein argonaute 1<br>**PDBTitle:** structural insights into small rna sorting and mrna binding by arabidopsis ago domains<br>**PDB Entry:** PDBe RCSB PDBj |
| 49 | c2xfmA_ | Alignment | not modelled | 98.5 | 19 | **PDB header:** rna/protein<br>**Chain:** A: **PDB Molecule:** PIWI-LIKE PROTEIN 1<br>**PDBTitle:** complex structure of the miwi paz domain bound to methylated single stranded rna<br>**PDB Entry:** PDBe RCSB PDBj |
| 50 | c3o3iA_ | Alignment | not modelled | 98.6 | 20 | **PDB header:** rna binding protein<br>**Chain:** A: **PDB Molecule:** Piwi-like protein 1<br>**PDBTitle:** ;crystal structure of human hiwi1 paz domain (residues 277-399) in complex with 14-mer rna (12-bp + 2-nt overhang) containing 2-oh at its 3-end;<br>**PDB Entry:** PDBe RCSB PDBj |
| 51 | c4g0mA_ | Alignment | not modelled | 98.4 | 14 | **PDB header:** gene regulation<br>**Chain:** A: **PDB Molecule:** Protein argonaute 2<br>**PDBTitle:** crystal structure of arabidopsis thaliana ago2 mid domain<br>**PDB Entry:** PDBe RCSB PDBj |
| 52 | c4g0xA_ | Alignment | not modelled | 99.4 | 13 | **PDB header:** gene regulation<br>**Chain:** A: **PDB Molecule:** Protein argonaute 1<br>**PDBTitle:** crystal structure of arabidopsis thaliana ago1 mid domain<br>**PDB Entry:** PDBe RCSB PDBj |
| 53 | c1r4kA_ | Alignment | not modelled | 97.1 | 13 | **PDB header:** rna binding protein<br>**Chain:** A: **PDB Molecule:** Argonaute 1<br>**PDBTitle:** solution structure of the drosophila argonaute 1 paz domain<br>**PDB Entry:** PDBe RCSB PDBj |
| 54 | c1r6zC_ | Alignment | not modelled | 97.4 | 15 | **PDB header:** gene regulation<br>**Chain:** C: **PDB Molecule:** Chimera of Maltose-binding periplasmic protein and Argonaute 2<br>**PDBTitle:** the crystal structure of the argonaute2 paz domain (as a mbp fusion)<br>**PDB Entry:** PDBe RCSB PDBj |
| 55 | c3qirD_ | Alignment | not modelled | 98.7 | 15 | **PDB header:** rna binding protein<br>**Chain:** D: **PDB Molecule:** Piwi-like protein 2<br>**PDBTitle:** crystal structure of piwil2 paz domain<br>**PDB Entry:** PDBe RCSB PDBj |
| 56 | c4g0oA_ | Alignment | not modelled | 99.1 | 10 | **PDB header:** gene regulation<br>**Chain:** A: **PDB Molecule:** Protein argonaute 5<br>**PDBTitle:** crystal structure of arabidopsis thaliana ago5 mid domain<br>**PDB Entry:** PDBe RCSB PDBj |
| 57 | c2fflA_ | Alignment | not modelled | 92.4 | 14 | **PDB header:** translation,hydrolase<br>**Chain:** A: **PDB Molecule:** Dicer<br>**PDBTitle:** crystal structure of dicer from giardia intestinalis<br>**PDB Entry:** PDBe RCSB PDBj |
| 58 | c3mj0A_ | Alignment | not modelled | 95.8 | 14 | **PDB header:** rna/rna binding protein<br>**Chain:** A: **PDB Molecule:** Protein argonaute-2<br>**PDBTitle:** ;crystal structure of drosophia ago-paz domain in complex with 3-end 2-o-methylated rna;<br>**PDB Entry:** PDBe RCSB PDBj |
| 59 | c4p1zA_ | Alignment | not modelled | 99.2 | 9 | **PDB header:** rna binding protein<br>**Chain:** A: **PDB Molecule:** Piwi-like protein 1<br>**PDBTitle:** structure of the mid domain from miwi<br>**PDB Entry:** PDBe RCSB PDBj |
| 60 | c1vynA_ | Alignment | not modelled | 94.9 | 14 | **PDB header:** nucleic acid binding<br>**Chain:** A: **PDB Molecule:** ARGONAUTE2<br>**PDBTitle:** structure and nucleic acid binding of the drosophila argonaute2 paz domain<br>**PDB Entry:** PDBe RCSB PDBj |
| 61 | c7xw2A_ | Alignment | not modelled | 85.3 | 11 | **PDB header:** gene regulation/rna<br>**Chain:** A: **PDB Molecule:** Endoribonuclease Dicer<br>**PDBTitle:** cryo-em structure of human dicer-pre-mirna in a dicing state<br>**PDB Entry:** PDBe RCSB PDBj |
| 62 | c8thqA_ | Alignment | not modelled | 91.2 | 12 | **PDB header:** rna binding protein/rna<br>**Chain:** A: **PDB Molecule:** Protein argonaute-2<br>**PDBTitle:** nonamer rna bound to hago2-paz<br>**PDB Entry:** PDBe RCSB PDBj |
| 63 | c4ngcA_ | Alignment | not modelled | 67.7 | 12 | **PDB header:** hydrolase/rna<br>**Chain:** A: **PDB Molecule:** Endoribonuclease Dicer<br>**PDBTitle:** ;structure of human dicer platform-paz-connector helix cassette in complex with 12-mer sirna having ua-3 ends (2.1 angstrom resolution);<br>**PDB Entry:** PDBe RCSB PDBj |
| 64 | c7vg2A_ | Alignment | not modelled | 82.0 | 7 | **PDB header:** rna binding protein/rna<br>**Chain:** A: **PDB Molecule:** Dicer-like 3<br>**PDBTitle:** cryo-em structure of arabidopsis dcl3 in complex with a 40-bp rna<br>**PDB Entry:** PDBe RCSB PDBj |
| | | | | | | **PDB header:** hydrolase/rna |

| | | | | | | |
|---|---|---|---|---|---|---|
| 65 | c4ngdA_ | Alignment | not modelled | 63.4 | 10 | **PDB header:** [illegible]<br>**Chain:** A: **PDB Molecule:** Endoribonuclease Dicer<br>**PDBTitle:** ;structure of human dicer platform-paz-connector helix cassette in complex with 12-mer sirna having 5-p and uu-3 ends (1.95 angstrom resolution);<br>**PDB Entry:** PDBe RCSB PDBj |
| 66 | c6ozeA_ | Alignment | not modelled | 22.1 | 27 | **PDB header:** hydrolase<br>**Chain:** A: **PDB Molecule:** Endonuclease V<br>**PDBTitle:** crystal structure of the catalytic domain of human endonuclease v (c140s/c225s/c226a/c228s)<br>**PDB Entry:** PDBe RCSB PDBj |
| 67 | c7yymA_ | Alignment | not modelled | 24.0 | 14 | **PDB header:** rna binding protein<br>**Chain:** A: **PDB Molecule:** Endoribonuclease Dicer<br>**PDBTitle:** ;mammalian dicer in the "pre-dicing state" with pre-mir-15a substrate;<br>**PDB Entry:** PDBe RCSB PDBj |
| 68 | c3gocA_ | Alignment | not modelled | 26.0 | 14 | **PDB header:** hydrolase<br>**Chain:** A: **PDB Molecule:** Endonuclease V<br>**PDBTitle:** ;crystal structure of the endonuclease v (sav1684) from streptomyces avermitilis. northeast structural genomics consortium target svr196;<br>**PDB Entry:** PDBe RCSB PDBj |
| 69 | c2w35A_ | Alignment | not modelled | 21.8 | 15 | **PDB header:** hydrolase<br>**Chain:** A: **PDB Molecule:** Endonuclease V<br>**PDBTitle:** structures of endonuclease v with dna reveal initiation of deaminated adenine repair<br>**PDB Entry:** PDBe RCSB PDBj |
| 70 | c2w36A_ | Alignment | not modelled | 18.4 | 16 | **PDB header:** hydrolase<br>**Chain:** A: **PDB Molecule:** ENDONUCLEASE V<br>**PDBTitle:** structures of endonuclease v with dna reveal initiation of deaminated adenine repair<br>**PDB Entry:** PDBe RCSB PDBj |
| 71 | c1z05A_ | Alignment | not modelled | 7.0 | 10 | **PDB header:** transcription<br>**Chain:** A: **PDB Molecule:** transcriptional regulator, ROK family<br>**PDBTitle:** crystal structure of the rok family transcriptional regulator, homolog of e.coli mlc protein.<br>**PDB Entry:** PDBe RCSB PDBj |
