# Supplementary material for: From CRISPR–Cas to Argonautes: Defense systems of bacteria and archaea in the hydrothermal deep subsurface
Source: ISME Commun. 2026 Jul 10;6(1):ycag200. doi: 10.1093/ismeco/ycag200 (PMC13477220; doi:10.1093/ismeco/ycag200)
Supplement: Supplementary file 1 [file supplementary_material_ycag200.zip › Supplementary_Materials_ycag200.zip!/_SRR22580803_bin_1_fna-.pdf]

Phyre2.2

Email paraskevi.mara@gmail.com

Description _SRR22580803_bin_1_fna-_00806_hypothetical_protein_Bathyarchaeia

Date Mon Dec 22 19:15:20 GMT 2025

Unique Job ID 29e0d9ae7d3f2a7b

Detailed template information

#	Template	Alignment Coverage	3D Model	Confidence	% i.d.	Template Information
1	c7yfqC_	Alignment	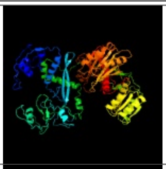	100.0	17	**PDB header:** rna binding protein/rna<br>**Chain:** C: **PDB Molecule:** Piwi<br>**PDBTitle:** cryo-em structure of the efpiwi (n959k)-pirna-target ternary complex<br>**PDB Entry:** PDBe RCSB PDBj
2	c8xihB_	Alignment	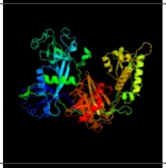	100.0	17	**PDB header:** dna binding protein/dna<br>**Chain:** B: **PDB Molecule:** Piwi domain-containing protein<br>**PDBTitle:** protein-dna complex<br>**PDB Entry:** PDBe RCSB PDBj
3	c7kx9A_	Alignment	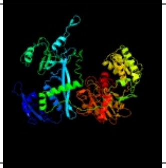	100.0	16	**PDB header:** hydrolase,rna binding protein/rna<br>**Chain:** A: **PDB Molecule:** Piwi-A<br>**PDBTitle:** cryo-em structure of ephydatia fluviatilis piwia-pirna-target complex<br>**PDB Entry:** PDBe RCSB PDBj
4	c7r8iA_	Alignment	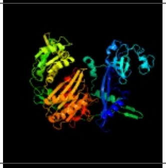	100.0	18	**PDB header:** hydrolase/dna<br>**Chain:** A: **PDB Molecule:** Argonaute<br>**PDBTitle:** ;crystal structure of pseudooceanicola lipolyticus argonaute bound to 5 oh guide dna in the presence of mg2+;<br>**PDB Entry:** PDBe RCSB PDBj
5	c7r8gA_	Alignment	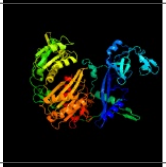	100.0	18	**PDB header:** hydrolase/dna<br>**Chain:** A: **PDB Molecule:** Argonaute<br>**PDBTitle:** ;crystal structure of pseudooceanicola lipolyticus argonaute bound to 5 oh guide dna;<br>**PDB Entry:** PDBe RCSB PDBj
6	c5guhA_	Alignment	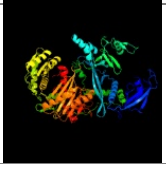	100.0	15	**PDB header:** hydrolase/rna<br>**Chain:** A: **PDB Molecule:** PIWI<br>**PDBTitle:** crystal structure of silkworm piwi-clade argonaute siwi bound to pirna<br>**PDB Entry:** PDBe RCSB PDBj
7	c1yvuA_	Alignment	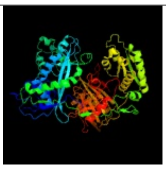	100.0	16	**PDB header:** rna binding protein, gene regulation<br>**Chain:** A: **PDB Molecule:** hypothetical protein aq_1447<br>**PDBTitle:** crystal structure of a. aeolicus argonaute<br>**PDB Entry:** PDBe RCSB PDBj
8	c2f8sC_	Alignment	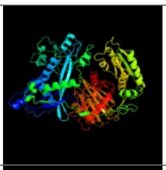	100.0	15	**PDB header:** rna binding protein/rna<br>**Chain:** C: **PDB Molecule:** Argonaute protein<br>**PDBTitle:** crystal structure of aa-ago with externally-bound sirna<br>**PDB Entry:** PDBe RCSB PDBj
9	c3dlbA_	Alignment	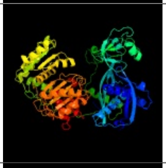	100.0	16	**PDB header:** nucleic acid binding protein/dna<br>**Chain:** A: **PDB Molecule:** argonaute<br>**PDBTitle:** crystal structure of the guide-strand-containing argonaute protein silencing complex<br>**PDB Entry:** PDBe RCSB PDBj
10	c8jpxA_	Alignment	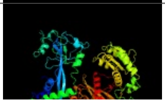	100.0	15	**PDB header:** gene regulation<br>**Chain:** A: **PDB Molecule:** Protein argonaute<br>**PDBTitle:** cryo-em structure of pfago-guide dna-target dna complex

			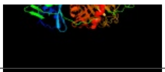			[illegible]<br>**PDB Entry:** PDBe RCSB PDBj
11	c7yfxA_	Alignment	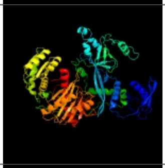	100.0	14	**PDB header:** rna binding protein/rna<br>**Chain:** A: **PDB Molecule:** Piwi-like protein 2<br>**PDBTitle:** cryo-em structure of hili in complex with pirna<br>**PDB Entry:** PDBe RCSB PDBj
12	c8jpxD_	Alignment	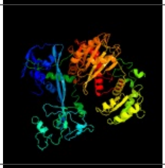	100.0	15	**PDB header:** gene regulation<br>**Chain:** D: **PDB Molecule:** Protein argonaute<br>**PDBTitle:** cryo-em structure of pfago-guide dna-target dna complex<br>**PDB Entry:** PDBe RCSB PDBj
13	c6kr6A_	Alignment	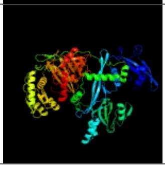	100.0	14	**PDB header:** rna binding protein/rna<br>**Chain:** A: **PDB Molecule:** Protein piwi<br>**PDBTitle:** crystal structure of drosophila piwi<br>**PDB Entry:** PDBe RCSB PDBj

14	c4ncbA_	Alignment	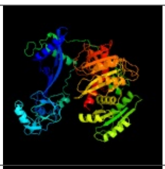	100.0	17	**PDB header:** nuclear protein/dna<br>**Chain:** A: **PDB Molecule:** Argonaute<br>**PDBTitle:** structure of thermus thermophilus argonaute bound to guide dna and 19-mer target dna with mg2+<br>**PDB Entry:** PDBe RCSB PDBj
15	c1u04A_	Alignment	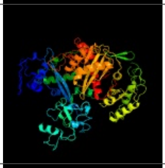	100.0	15	**PDB header:** hydrolase/gene regulation<br>**Chain:** A: **PDB Molecule:** hypothetical protein PF0537<br>**PDBTitle:** crystal structure of full length argonaute from pyrococcus furiosus<br>**PDB Entry:** PDBe RCSB PDBj
16	c6d92A_	Alignment	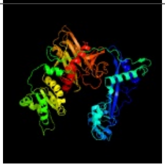	100.0	16	**PDB header:** rna binding protein<br>**Chain:** A: **PDB Molecule:** Uncharacterized protein<br>**PDBTitle:** ternary rsago complex with guide rna and target dna containing a-a non-canonical pair at position 3<br>**PDB Entry:** PDBe RCSB PDBj
17	c6d92D_	Alignment	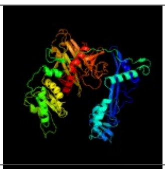	100.0	16	**PDB header:** rna binding protein<br>**Chain:** D: **PDB Molecule:** Uncharacterized protein<br>**PDBTitle:** ternary rsago complex with guide rna and target dna containing a-a non-canonical pair at position 3<br>**PDB Entry:** PDBe RCSB PDBj
18	c7svaA_	Alignment	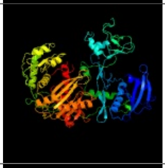	100.0	13	**PDB header:** gene regulation<br>**Chain:** A: **PDB Molecule:** Protein argonaute 10<br>**PDBTitle:** cryo-em structure of arabidopsis ago10-guide rna complex<br>**PDB Entry:** PDBe RCSB PDBj
19	c8wd8A_	Alignment	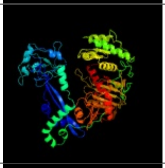	100.0	15	**PDB header:** dna binding protein/dna<br>**Chain:** A: **PDB Molecule:** Argonaute family protein<br>**PDBTitle:** cryo-em structure of ttdago-guide dna-target dna complex<br>**PDB Entry:** PDBe RCSB PDBj
20	c6nitA_	Alignment	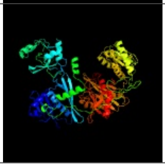	100.0	12	**PDB header:** rna binding protein/rna<br>**Chain:** A: **PDB Molecule:** Protein argonaute-2<br>**PDBTitle:** human argonaute2-mir-122 bound to a target rna with four central mismatches (bu4)<br>**PDB Entry:** PDBe RCSB PDBj
21	c5vm9C_	Alignment	not modelled	100.0	12	**PDB header:** hydrolase/rna<br>**Chain:** C: **PDB Molecule:** Protein argonaute-3<br>**PDBTitle:** human argonaute3 bound to guide rna<br>**PDB Entry:** PDBe RCSB PDBj
22	c6oonA_	Alignment	not modelled	100.0	12	**PDB header:** hydrolase/rna<br>**Chain:** A: **PDB Molecule:** Protein argonaute-4<br>**PDBTitle:** human argonaute4 bound to guide rna<br>**PDB Entry:** PDBe RCSB PDBj
23	c8vajA	Alignment	not modelled	100.0	13	**PDB header:** rna binding protein/rna<br>**Chain:** A: **PDB Molecule:** Protein argonaute-3<br>[illegible]

		Alignment				**PDBTitle:** human argonaute3 bound to cityrna and target rna<br>**PDB Entry:** PDBe RCSB PDBj
24	c5g5sA_	Alignment	not modelled	100.0	13	**PDB header:** hydrolase<br>**Chain:** A: **PDB Molecule:** ARGONAUTE<br>**PDBTitle:** structure of the argonaute protein from methanocaldcoccus janaschii<br>**PDB Entry:** PDBe RCSB PDBj
25	c4w5tA_	Alignment	not modelled	100.0	11	**PDB header:** hydrolase/rna<br>**Chain:** A: **PDB Molecule:** Protein argonaute-2<br>**PDBTitle:** the crystal structure of human argonaute2 bound to a guide and target rna containing seed pairing from 2-7<br>**PDB Entry:** PDBe RCSB PDBj
26	c4z4dA_	Alignment	not modelled	100.0	11	**PDB header:** gene regulation/rna<br>**Chain:** A: **PDB Molecule:** Protein argonaute-2<br>**PDBTitle:** human argonaute2 bound to t1-g target rna<br>**PDB Entry:** PDBe RCSB PDBj
27	c6n4oA_	Alignment	not modelled	100.0	11	**PDB header:** rna binding protein/rna<br>**Chain:** A: **PDB Molecule:** Protein argonaute-2<br>**PDBTitle:** human argonaute2-mir-122 bound to a seed and supplementary paired target<br>**PDB Entry:** PDBe RCSB PDBj
28	c6mdzA_	Alignment	not modelled	100.0	11	**PDB header:** hydrolase/rna<br>**Chain:** A: **PDB Molecule:** Protein argonaute-2<br>**PDBTitle:** human argonaute2-mir-122 bound to a target rna with two central mismatches (bu2)<br>**PDB Entry:** PDBe RCSB PDBj
29	c8ffiB_	Alignment	not modelled	100.0	20	**PDB header:** immune system<br>**Chain:** B: **PDB Molecule:** short pAgo<br>**PDBTitle:** structure of tetramerized mapsparta upon guide rna-mediated target dna binding<br>**PDB Entry:** PDBe RCSB PDBj
30	c8fexB_	Alignment	not modelled	100.0	20	**PDB header:** immune system<br>**Chain:** B: **PDB Molecule:** short pAgo<br>**PDBTitle:** inactivate state of maribacter polysiphoniae argonuate (short pago system)<br>**PDB Entry:** PDBe RCSB PDBj
31	c4kreA_	Alignment	not modelled	100.0	12	**PDB header:** transcription/rna<br>**Chain:** A: **PDB Molecule:** Protein argonaute-1<br>**PDBTitle:** structure of human argonaute-1 bound to endogenous sf9 rna<br>**PDB Entry:** PDBe RCSB PDBj
32	c8isyA_	Alignment	not modelled	100.0	19	**PDB header:** dna binding protein<br>**Chain:** A: **PDB Molecule:** Piwi domain-containing protein<br>**PDBTitle:** cryo-em structure of free-state crt-sparta<br>**PDB Entry:** PDBe RCSB PDBj
33	c8ifkB_	Alignment	not modelled	100.0	20	**PDB header:** rna binding protein/rna/dna<br>**Chain:** B: **PDB Molecule:** Piwi domain-containing protein<br>**PDBTitle:** cryo-em structure of monomeric sparta grna-ssdna target complex<br>**PDB Entry:** PDBe RCSB PDBj
34	c8u72D_	Alignment	not modelled	100.0	19	**PDB header:** rna binding protein/rna/dna<br>**Chain:** D: **PDB Molecule:** Piwi domain-containing protein<br>**PDBTitle:** cryo-em structure of the sparta oligomer with guide rna and target dna<br>**PDB Entry:** PDBe RCSB PDBj
35	c6t5tA_	Alignment	not modelled	100.0	19	**PDB header:** dna binding protein<br>**Chain:** A: **PDB Molecule:** Piwi protein AF_1318<br>**PDBTitle:** ;crystal structure of archaeoglobus fulgidus argonaute protein with cognate dna oligoduplex 5-pattgtggccacaat;<br>**PDB Entry:** PDBe RCSB PDBj
36	c8qg0A_	Alignment	not modelled	100.0	18	**PDB header:** dna binding protein<br>**Chain:** A: **PDB Molecule:** Piwi protein<br>**PDBTitle:** archaeoglobus fulgidus afago complex with afago-n protein (fafago) bound with 17 nt rna guide and 17 nt dna target<br>**PDB Entry:** PDBe RCSB PDBj
37	c8pvvA_	Alignment	not modelled	100.0	18	**PDB header:** dna binding protein<br>**Chain:** A: **PDB Molecule:** Piwi protein<br>**PDBTitle:** archaeoglobus fulgidus afago complex with afago-n protein (fafago) bound with 30 nt rna guide and 51 nt dna target<br>**PDB Entry:** PDBe RCSB PDBj
38	c5i4aC_	Alignment	not modelled	100.0	13	**PDB header:** rna binding protein/rna<br>**Chain:** C: **PDB Molecule:** Argonaute protein<br>**PDBTitle:** ;x-ray crystal structure of marinitoga piezophila argonaute in complex with 5 oh guide rna;<br>**PDB Entry:** PDBe RCSB PDBj
39	c8k88C_	Alignment	not modelled	100.0	17	**PDB header:** dna/rna/cell invasion<br>**Chain:** C: **PDB Molecule:** Piwi domain protein<br>**PDBTitle:** structure of procaryotic ago<br>**PDB Entry:** PDBe RCSB PDBj
40	c8jl0B_	Alignment	not modelled	100.0	17	**PDB header:** antiviral protein/dna/rna<br>**Chain:** B: **PDB Molecule:** Piwi domain protein<br>**PDBTitle:** cryo-em structure of the prokaryotic sparsa system complex<br>**PDB Entry:** PDBe RCSB PDBj
41	c8in8B_	Alignment	not modelled	100.0	17	**PDB header:** antiviral protein<br>**Chain:** B: **PDB Molecule:** Piwi domain protein<br>**PDBTitle:** cryo-em structure of the target ssdna-bound sir2-apaz/ago-grna quaternary complex<br>**PDB Entry:** PDBe RCSB PDBj
42	c9iizA_	Alignment	not modelled	100.0	17	**PDB header:** rna binding protein/rna<br>**Chain:** A: **PDB Molecule:** Piwi<br>**PDBTitle:** cryo-em structure of efpiwi-pirna-target (25-nt, comma)<br>**PDB Entry:** PDBe RCSB PDBj
43	c5theE_	Alignment	not modelled	100.0	13	**PDB header:** rna binding protein/rna<br>**Chain:** E: **PDB Molecule:** Uncharacterized protein<br>**PDBTitle:** crystal structure of the c-terminal lobe of a budding yeast argonaute<br>**PDB Entry:** PDBe RCSB PDBj
44	c5theG_	Alignment	not modelled	100.0	12	**PDB header:** rna binding protein/rna<br>**Chain:** G: **PDB Molecule:** Uncharacterized protein<br>**PDBTitle:** crystal structure of the c-terminal lobe of a budding yeast

		Alignment				argonaute<br>**PDB Entry:** PDBe RCSB PDBj
45	c9ij2A_	Alignment	not modelled	100.0	13	**PDB header:** rna binding protein/rna<br>**Chain:** A: **PDB Molecule:** Piwi-like protein 2<br>**PDBTitle:** cryo-em structure of mili-pirna-target (22-nt, comma)<br>**PDB Entry:** PDBe RCSB PDBj
46	c2yhaA_	Alignment	not modelled	100.0	11	**PDB header:** rna binding protein<br>**Chain:** A: **PDB Molecule:** POST-TRANSCRIPTIONAL GENE SILENCING PROTEIN QDE-2<br>**PDBTitle:** crystal structure of the n. crassa qde-2 ago mid-piwi domains<br>**PDB Entry:** PDBe RCSB PDBj
47	c2l5cA_	Alignment	not modelled	99.6	18	**PDB header:** rna binding protein<br>**Chain:** A: **PDB Molecule:** Piwi-like protein 1<br>**PDBTitle:** solution structures of human piwi-like 1 paz domain<br>**PDB Entry:** PDBe RCSB PDBj
48	c3qirD_	Alignment	not modelled	99.5	20	**PDB header:** rna binding protein<br>**Chain:** D: **PDB Molecule:** Piwi-like protein 2<br>**PDBTitle:** crystal structure of piwil2 paz domain<br>**PDB Entry:** PDBe RCSB PDBj
49	c3o3iA_	Alignment	not modelled	99.4	20	**PDB header:** rna binding protein<br>**Chain:** A: **PDB Molecule:** Piwi-like protein 1<br>**PDBTitle:** ;crystal structure of human hiwi1 paz domain (residues 277-399) in complex with 14-mer rna (12-bp + 2-nt overhang) containing 2-oh at its 3-end;<br>**PDB Entry:** PDBe RCSB PDBj
50	c2xfmA_	Alignment	not modelled	99.4	19	**PDB header:** rna/protein<br>**Chain:** A: **PDB Molecule:** PIWI-LIKE PROTEIN 1<br>**PDBTitle:** complex structure of the miwi paz domain bound to methylated single stranded rna<br>**PDB Entry:** PDBe RCSB PDBj
51	c4g0oA_	Alignment	not modelled	99.3	14	**PDB header:** gene regulation<br>**Chain:** A: **PDB Molecule:** Protein argonaute 5<br>**PDBTitle:** crystal structure of arabidopsis thaliana ago5 mid domain<br>**PDB Entry:** PDBe RCSB PDBj
52	c4p1zA_	Alignment	not modelled	99.0	15	**PDB header:** rna binding protein<br>**Chain:** A: **PDB Molecule:** Piwi-like protein 1<br>**PDBTitle:** structure of the mid domain from miwi<br>**PDB Entry:** PDBe RCSB PDBj
53	c3vnbA_	Alignment	not modelled	99.4	13	**PDB header:** rna binding protein<br>**Chain:** A: **PDB Molecule:** Protein argonaute 1<br>**PDBTitle:** structural insights into small rna sorting and mrna binding by arabidopsis ago domains<br>**PDB Entry:** PDBe RCSB PDBj
54	c4g0xA_	Alignment	not modelled	99.4	13	**PDB header:** gene regulation<br>**Chain:** A: **PDB Molecule:** Protein argonaute 1<br>**PDBTitle:** crystal structure of arabidopsis thaliana ago1 mid domain<br>**PDB Entry:** PDBe RCSB PDBj
55	c1r4kA_	Alignment	not modelled	98.5	11	**PDB header:** rna binding protein<br>**Chain:** A: **PDB Molecule:** Argonaute 1<br>**PDBTitle:** solution structure of the drosophila argonaute 1 paz domain<br>**PDB Entry:** PDBe RCSB PDBj
56	c3mj0A_	Alignment	not modelled	98.2	15	**PDB header:** rna/rna binding protein<br>**Chain:** A: **PDB Molecule:** Protein argonaute-2<br>**PDBTitle:** ;crystal structure of drosophia ago-paz domain in complex with 3-end 2-o-methylated rna;<br>**PDB Entry:** PDBe RCSB PDBj
57	c1r6zC_	Alignment	not modelled	98.4	14	**PDB header:** gene regulation<br>**Chain:** C: **PDB Molecule:** Chimera of Maltose-binding periplasmic protein and Argonaute 2<br>**PDBTitle:** the crystal structure of the argonaute2 paz domain (as a mbp fusion)<br>**PDB Entry:** PDBe RCSB PDBj
58	c8thqA_	Alignment	not modelled	97.8	14	**PDB header:** rna binding protein/rna<br>**Chain:** A: **PDB Molecule:** Protein argonaute-2<br>**PDBTitle:** nonamer rna bound to hago2-paz<br>**PDB Entry:** PDBe RCSB PDBj
59	c1vynA_	Alignment	not modelled	97.9	14	**PDB header:** nucleic acid binding<br>**Chain:** A: **PDB Molecule:** ARGONAUTE2<br>**PDBTitle:** structure and nucleic acid binding of the drosophila argonaute2 paz domain<br>**PDB Entry:** PDBe RCSB PDBj
60	c7vg2A_	Alignment	not modelled	97.5	17	**PDB header:** rna binding protein/rna<br>**Chain:** A: **PDB Molecule:** Dicer-like 3<br>**PDBTitle:** cryo-em structure of arabidopsis dcl3 in complex with a 40-bp rna<br>**PDB Entry:** PDBe RCSB PDBj
61	c8dgiA_	Alignment	not modelled	95.9	15	**PDB header:** rna binding protein/rna<br>**Chain:** A: **PDB Molecule:** Endoribonuclease Dcr-1<br>**PDBTitle:** structural basis of microrna biogenesis by dicer-1 and its partner protein loqs-pb - complex ia<br>**PDB Entry:** PDBe RCSB PDBj
62	c8dg5A_	Alignment	not modelled	95.7	15	**PDB header:** rna binding protein/rna<br>**Chain:** A: **PDB Molecule:** Endoribonuclease Dcr-1<br>**PDBTitle:** structural basis of microrna biogenesis by dicer-1 and its partner protein loqs-pb - complex iib<br>**PDB Entry:** PDBe RCSB PDBj
63	c4g0mA_	Alignment	not modelled	98.0	10	**PDB header:** gene regulation<br>**Chain:** A: **PDB Molecule:** Protein argonaute 2<br>**PDBTitle:** crystal structure of arabidopsis thaliana ago2 mid domain<br>**PDB Entry:** PDBe RCSB PDBj
64	c2fflA_	Alignment	not modelled	97.9	12	**PDB header:** translation,hydrolase<br>**Chain:** A: **PDB Molecule:** Dicer<br>**PDBTitle:** crystal structure of dicer from giardia intestinalis<br>**PDB Entry:** PDBe RCSB PDBj
65	c7w0cA_	Alignment	not modelled	96.1	12	**PDB header:** rna binding protein<br>**Chain:** A: **PDB Molecule:** Dicer-2, isoform A<br>**PDBTitle:** dicer2-loqs-pd-dsrna complex at early-translocation state<br>**PDB Entry:** PDBe RCSB PDBj
						[illegible]

66	c7w0aA_	Alignment	not modelled	95.2	12	**PDB header:** rna binding protein<br>**Chain:** A: **PDB Molecule:** Dicer-2, isoform A<br>**PDBTitle:** dmdicer2-loqspd-dsrna dimer status<br>**PDB Entry:** PDBe RCSB PDBj
67	c7yymA_	Alignment	not modelled	94.1	12	**PDB header:** rna binding protein<br>**Chain:** A: **PDB Molecule:** Endoribonuclease Dicer<br>**PDBTitle:** ;mammalian dicer in the "pre-dicing state" with pre-mir-15a substrate;<br>**PDB Entry:** PDBe RCSB PDBj
68	c4ngcA_	Alignment	not modelled	97.0	9	**PDB header:** hydrolase/rna<br>**Chain:** A: **PDB Molecule:** Endoribonuclease Dicer<br>**PDBTitle:** ;structure of human dicer platform-paz-connector helix cassette in complex with 12-mer sirna having ua-3 ends (2.1 angstrom resolution);<br>**PDB Entry:** PDBe RCSB PDBj
69	c4ngdA_	Alignment	not modelled	96.8	9	**PDB header:** hydrolase/rna<br>**Chain:** A: **PDB Molecule:** Endoribonuclease Dicer<br>**PDBTitle:** ;structure of human dicer platform-paz-connector helix cassette in complex with 12-mer sirna having 5-p and uu-3 ends (1.95 angstrom resolution);<br>**PDB Entry:** PDBe RCSB PDBj
70	c7xw2A_	Alignment	not modelled	97.5	9	**PDB header:** gene regulation/rna<br>**Chain:** A: **PDB Molecule:** Endoribonuclease Dicer<br>**PDBTitle:** cryo-em structure of human dicer-pre-mirna in a dicing state<br>**PDB Entry:** PDBe RCSB PDBj
71	c7xw3A_	Alignment	not modelled	94.7	9	**PDB header:** gene regulation<br>**Chain:** A: **PDB Molecule:** Endoribonuclease Dicer<br>**PDBTitle:** cryo-em structure of an apo-form of human dicer<br>**PDB Entry:** PDBe RCSB PDBj
72	c7kpvD_	Alignment	not modelled	15.8	12	**PDB header:** transcription<br>**Chain:** D: **PDB Molecule:** Mediator of RNA polymerase II transcription subunit 13<br>**PDBTitle:** structure of kinase and central lobes of yeast ckm<br>**PDB Entry:** PDBe RCSB PDBj
73	c2ch5A_	Alignment	not modelled	25.1	13	**PDB header:** transferase<br>**Chain:** A: **PDB Molecule:** NAGK PROTEIN<br>**PDBTitle:** crystal structure of human n-acetylglucosamine kinase in complex with n-acetylglucosamine<br>**PDB Entry:** PDBe RCSB PDBj
74	c5huxA_	Alignment	not modelled	28.2	16	**PDB header:** transferase<br>**Chain:** A: **PDB Molecule:** Probable sugar kinase<br>**PDBTitle:** putative sugar kinases from synechococcus elongatus pcc7942 in complex with adp<br>**PDB Entry:** PDBe RCSB PDBj
75	c5htnA_	Alignment	not modelled	25.5	16	**PDB header:** transferase<br>**Chain:** A: **PDB Molecule:** Probable sugar kinase<br>**PDBTitle:** putative sugar kinases from synechococcus elongatus pcc7942-apo form<br>**PDB Entry:** PDBe RCSB PDBj
76	c8oqkA_	Alignment	not modelled	26.3	10	**PDB header:** transferase<br>**Chain:** A: **PDB Molecule:** N-acetylglucosamine kinase<br>**PDBTitle:** crystal structure of tannerella forsythia sugar kinase k1058<br>**PDB Entry:** PDBe RCSB PDBj
77	c1zxoC_	Alignment	not modelled	24.9	9	**PDB header:** unknown function<br>**Chain:** C: **PDB Molecule:** conserved hypothetical protein Q8A1P1<br>**PDBTitle:** x-ray crystal structure of protein q8a1p1 from bacteroides thetaiotaomicron. northeast structural genomics consortium target btr25.<br>**PDB Entry:** PDBe RCSB PDBj
78	c1zxoD_	Alignment	not modelled	24.9	9	**PDB header:** unknown function<br>**Chain:** D: **PDB Molecule:** conserved hypothetical protein Q8A1P1<br>**PDBTitle:** x-ray crystal structure of protein q8a1p1 from bacteroides thetaiotaomicron. northeast structural genomics consortium target btr25.<br>**PDB Entry:** PDBe RCSB PDBj
79	c2e2nA_	Alignment	not modelled	18.0	12	**PDB header:** transferase<br>**Chain:** A: **PDB Molecule:** HEXOKINASE<br>**PDBTitle:** crystal structure of sulfolobus tokodaii hexokinase in the apo form<br>**PDB Entry:** PDBe RCSB PDBj
80	c2ch6A_	Alignment	not modelled	18.0	11	**PDB header:** transferase<br>**Chain:** A: **PDB Molecule:** N-ACETYL-D-GLUCOSAMINE KINASE<br>**PDBTitle:** crystal structure of human n-acetylglucosamine kinase in complex with adp and glucose<br>**PDB Entry:** PDBe RCSB PDBj
81	c2ch6D_	Alignment	not modelled	18.0	11	**PDB header:** transferase<br>**Chain:** D: **PDB Molecule:** N-ACETYL-D-GLUCOSAMINE KINASE<br>**PDBTitle:** crystal structure of human n-acetylglucosamine kinase in complex with adp and glucose<br>**PDB Entry:** PDBe RCSB PDBj
82	c1zbsA_	Alignment	not modelled	18.5	11	**PDB header:** structural genomics, unknown function<br>**Chain:** A: **PDB Molecule:** hypothetical protein PG1100<br>**PDBTitle:** ;crystal structure of the putative n-acetylglucosamine kinase (pg1100) from porphyromonas gingivalis, northeast structural genomics target pgr18;<br>**PDB Entry:** PDBe RCSB PDBj
83	c7xn7S_	Alignment	not modelled	6.7	15	**PDB header:** transcription<br>**Chain:** S: **PDB Molecule:** Transcription elongation factor Spt6<br>**PDBTitle:** rna polymerase ii elongation complex containing spt4/5, elf1, spt6, spn1 and paf1c<br>**PDB Entry:** PDBe RCSB PDBj
84	c4ehuA_	Alignment	not modelled	7.5	18	**PDB header:** electron transport<br>**Chain:** A: **PDB Molecule:** Activator of 2-hydroxyisocaproyl-CoA dehydratase<br>**PDBTitle:** activator of the 2-hydroxyisocaproyl-coa dehydratase from clostridium difficile with bound adpnp<br>**PDB Entry:** PDBe RCSB PDBj
85	c1z05A_	Alignment	not modelled	8.3	12	**PDB header:** transcription<br>**Chain:** A: **PDB Molecule:** transcriptional regulator, ROK family<br>**PDBTitle:** crystal structure of the rok family transcriptional regulator, homolog of e.coli mlc protein.<br>**PDB Entry:** PDBe RCSB PDBj

86	c3da5A_	Alignment	not modelled	11.1	21	**PDB header:** rna binding protein<br>**Chain:** A: **PDB Molecule:** Argonaute<br>**PDBTitle:** crystal structure of piwi/argonaute/zwille(paz) domain from thermococcus thioreducens<br>**PDB Entry:** PDBe RCSB PDBj
87	c4ijaA_	Alignment	not modelled	5.8	17	**PDB header:** protein binding<br>**Chain:** A: **PDB Molecule:** XylR protein<br>**PDBTitle:** structure of s. aureus methicillin resistance factor mecr2<br>**PDB Entry:** PDBe RCSB PDBj
88	c1woqA_	Alignment	not modelled	7.0	13	**PDB header:** transferase<br>**Chain:** A: **PDB Molecule:** Inorganic polyphosphate/ATP-glucomannokinase<br>**PDBTitle:** crystal structure of inorganic polyphosphate/atp-glucomannokinase from arthrobacter sp. strain km at 1.8 a resolution<br>**PDB Entry:** PDBe RCSB PDBj
