# Supplementary material for: From CRISPR–Cas to Argonautes: Defense systems of bacteria and archaea in the hydrothermal deep subsurface
Source: ISME Commun. 2026 Jul 10;6(1):ycag200. doi: 10.1093/ismeco/ycag200 (PMC13477220; doi:10.1093/ismeco/ycag200)
Supplement: Supplementary file 1 [file supplementary_material_ycag200.zip › Supplementary_Materials_ycag200.zip!/Supplementary File 1 revised.docx]

**Supplementary File 1. Documentation of** **Argonaute nucleotide sequences, protein domains, sub-domains and conserved sites (pages 1 to 18), and protein alignments (PAUP files) used for phylogenetic analyses** (pages 19-25). Based on nucleotide sequences from metagenomes, translated protein sequences were determined, and their domain structure was checked by BlastP searches. Protein-based sub-domains are highlighted in color; conserved key positions in RNA or DNA binding motifs, and active RNA-slicing sites, are highlighted in bold.

**>12_bin.11.fna_01717 hypothetical protein**

Nucleotide sequence:

ATGTCGCTTATGGCGAATGTATTTGAGGCAAATGTTAGATTTGATAAATCCGTCGCTAACTTTGACTTTTTCGAGGTTGGAGGGGTTGAGTCTGTTTCGAGGCGGTATGAATTATTGGGTATCGTTGTGACACATCTCCAGAGGGAAGAAAGAAAACCCTTTGCCACTTATACTCCTTCTGATACAGAGAATTATGTCATCGGTTTTTTGCCTAATGGACCAAAGAAAAAACAGTTTGGCATAGAGGGTCTTAGAGTTCGGTACCTGGGGAAAGATGAGCTACCACCCTCTCCCCCAGCCTATAGAACTCTAACAGAGATTTTGAACAAATCAAAATTTTGGGAACTCAAAGCGTATATGTGGGCCTTAGGTCTTCATACTTTATACCCAAAACGCTGGAAAGACTTGAACGAAGACTATCGAGATTGTGGCCTTTGTTTATTCCGAGGACCATACTTTAGATATAATATTCTGCCAGATGGTCGAATTACGCTTACAATCGATAGCAAAACCCACTACGTCAAATCTGAATGTTTTTTGGATGAAATTAGACGTAGAGGCAGAGACCTCAAGTGGTTCAAAGAGGAGATGGAAGCGAAAAGAGAGTTTTATAGAAGGCTAGGGAAAAAATTCCGGGGCATACACTTCTACTACGCGCTAGCTCAACAAGATATTGCGATAAACGACATTGATACGCGTCCTATTTCACAAATTCCCCTTAAGGAAGAAGTTAAAGTTGGTGGAAGGTTATGTAGAACTATTCTCGAGTACTTGAAAGCAAAGTATAGAGATGGTCCGCGTGTAAGAGCAATAGATGAAACCCAGCCTGGCCTGAAAGGAGACGATTTGACGTTTGCCCCTCAGTTCCTTCATAAGTCTGTTGATCTAGAGGAAGTGGAAGACCTCATTCTTAACGAACAAACGTTCTATATGGACACGAGGGGTGTGAAGGGAAAGAGAGATATTAATCGACCCGCCAAGATGAGGTGGATCTTCATAACCAAGTATTTGCAAAGGTACTTCACTTATCTAGGTGTCGGCCCCTTTCTTGCAAAGTTTAGTGACCCAATCAGGTTTCCGCTTACGAAACACTTTGATAAACCGAGGCTACTGATTAAAGAGGGTAAAGAACCTGTCTCCTTTGAAGATCTTCCGAGGGCTCTAGCAAGAGGACCGTACAAAGACCCTAAGATACCGAGAGTCTACTTTTATTCTATGTTAGAGGGAGAGGAAGCAGTCTATAATAAGCAGTTCTATGGCGTTCTAAGGGACTTTGCGAAACGTAGGCTAGGCGTCTCCTTTCCTTCATCGGTGCTCCCTCTTAACAAGGATTTGGGCTCCGTTCGGATTTATCTTAAGAAATCTCTAGAGAAATATGGTAAGTTGGGTGGGGTCTGCCTTGGAATAGTTGAAGAGGAGTCTCCACTT**CAT**GACGAGCTC**ATA**ACGATGTTCGGAGAATACGGCTTGCCGGTT**CAGTGCGTTACA**AGTAAA**ACC**GCTATAGAGGACGTGTGTTTTTCTCGTAATGTCTAT**ATA**ACGGAG**AAC**CTCTGTGCC**ATG**ATGATAGCC**AAA**GCAGGTGGCATCCCTTGGGTTCTTTACGACGAACTGAATTACGGTTGCTATGGTGCAATA**GAC**GTA**GGA**AGAACAAAGGCTGAATATTGGGCTATGGCTGTGGTTTACAACCGGAAGGGCATGTTTGAGACGCTTCCAGGGAGAATAACTATAGGGGAAAGGCTTGATGAGCACAGTATTAAGTATTGTGTTGAGGAGGCTGCGAGATACGCACGTGATAGTTCTGATTTTATTTTCTTTAGA**CAT**GGAAGAATTTATCCTAATGAAGTCGTAGCTTTCGAAAATGTAACAGGGGAAAGTCCCTTTAAGAACTGTGCCATTGTTGCCATAAAAACAGGGGAAGTCCCATATAGAATATTTCGTGAAGTTAATGGGAAAGTGATCAAACCTTTGTCTGGTGATTACTATCCCCTAGGACAGTCAACCGTTGTTGTATGCACCGTGGGTGTTGATGAGTACAAACATGGCATGCCCAAACCAATCACTATTGAAGTTATCCCTACAAAAGGGGAAGTTGAGGCCGTGAAGGTGGCCAGGGACGCGTTCTATTTAACGTACTTAAATTGGGGATCGCCGAGACAGAGCTATTCAATGCCGGCTCCTTTAAGGCTCGCT**CAC**ACTTTAGCTTCTGAATTAAGCGCTGGAATTCGAACAGGCGGGCGACCGTTTTAG

Translated protein sequence:

MSLMANVFEANVRFDKSVANFDFFEVGGVESVSRRYELLGIVVTHLQREERKPFATYTPSDTENYVIGFLPNGPKKKQFGIEGLRVRYLGKDELPPSPPAYRTLTEILNKSKFWELKAYMWALGLHTLYPKRWKDLNEDYRDCGLCLFRGPYFRYNILPDGRITLTIDSKTHYVKSECFLDEIRRRGRDLKWFKEEMEAKREFYRRLGKKFRGIHFYYALAQQDIAINDIDTRPISQIPLKEEVKVGGRLCRTILEYLKAKYRDGPRVRAIDETQPGLKGDDLTFAPQFLHKSVDLEEVEDLILNEQTFYMDTRGVKGKRDINRPAKMRWIFITKYLQRYFTYLGVGPFLAKFSDPIRFPLTKHFDKPRLLIKEGKEPVSFEDLPRALARGPYKDPKIPRVYFYSMLEGEEAVYNKQFYGVLRDFAKRRLGVSFPSSVLPLNKDLGSVRIYLKKSLEKYGKLGGVCLGIVEEESPL**H**DEL**I**TMFGEYGLPV**QCVT**SK**T**AIEDVCFSRNVY**I**TE**N**LCA**M**MIA**K**AGGIPWVLYDELNYGCYGAI**D**V**G**RTKAEYWAMAVVYNRKGMFETLPGRITIGERLDEHSIKYCVEEAARYARDSSDFIFFR**H**GRIYPNEVVAFENVTGESPFKNCAIVAIKTGEVPYRIFREVNGKVIKPLSGDYYPLGQSTVVVCTVGVDEYKHGMPKPITIEVIPTKGEVEAVKVARDAFYLTYLNWGSPRQSYSMPAPLRLA**H**TLASELSAGIRTGGRPF*

Color annotation: The C-terminal PIWI domain (in green) contains the Piwi_piwi-lile_ProArk domain which in turns contains two subdomains, the 5' RNA guide strand anchoring site (in purple) and the catalytic site for slicing (in red). Conserved sites are marked in bold. Underlined positions mark the conserved **[H/R]KQ**TN**K** motif for short pAgos (Ryazansky et al. 2018).

BlastP search:

Comments:

cd04659: Piwi_piwi-like_ProArk: PIWI domain, Piwi-like subfamily found in Archaea and Bacteria. RNA silencing refers to a group of related gene-silencing mechanisms mediated by short RNA molecules, including siRNAs, miRNAs, and heterochromatin-related guide RNAs. The central component of the RNA-induced silencing complex (RISC) and related complexes is Argonaute. The PIWI domain is the C-terminal portion of Argonaute and consists of two subdomains, one of which provides the 5' anchoring of the guide RNA and the other, the catalytic site for slicing. This domain is also found in closely related proteins, including the Piwi subfamily, where it is believed to perform a crucial role in germline cells, via a similar mechanism.

**>7_bin.1.fna_01190 hypothetical protein**

Nucleotide sequence:

ATGTCGCTTATGGCGAATGTATTTGAGGCAAATGTTAGATTTGATAAATCCGTCGCTAACTTTGACTTTTTCGAGGTTGGAGGGGTTGAGTCTGTTTCGAGGCGGTATGAATTATTGGGTATCGTTGTGACACATCTCCAGAGGGAAGAAAGAAAACCCTTTGCCACTTATACTCCTTCTGATACAGAGAATTATGTCATCGGTTTTTTGCCTAATGGACCAAAGAAAAAACAGTTTGGCATAGAGGGTCTTAGAGTTCGGTACCTGGGGAAAGATGAGCTACCACCCTCTCCCCCAGCCTATAGAACTCTAACAGAGATTTTGAACAAATCAAAATTTTGGGAACTCAAAGCGTATATGTGGGCCTTAGGTCTTCATACTTTATACCCAAAACGCTGGAAAGACTTGAACGAAGACTATCGAGATTGTGGCCTTTGTTTATTCCGAGGACCATACTTTAGATATAATATTCTGCCAGATGGTCGAATTACGCTTACAATCGATAGCAAAACCCACTACGTCAAATCTGAATGTTTTTTGGATGAAATTAGACGTAGAGGCAGAGACCTCAAGTGGTTCAAAGAGGAGATGGAAGCGAAAAGAGAGTTTTATAGAAGGCTAGGGAAAAAATTCCGGGGCATACACTTCTACTACGCGCTAGCTCAACAAGATATTGCGATAAACGACATTGATACGCGTCCTATTTCACAAATTCCCCTTAAGGAAGAAGTTAAAGTTGGTGGAAGGTTATGTAGAACTATTCTCGAGTACTTGAAAGCAAAGTATAGAGATGGTCCGCGTGTAAGAGCAATAGATGAAACCCAGCCTGGCCTGAAAGGAGACGATTTGACGTTTGCCCCTCAGTTCCTTCATAAGTCTGTTGATCTAGAGGAAGTGGAAGACCTCATTCTTAACGAACAAACGTTCTATATGGACACGAGGGGTGTGAAGGGAAAGAGAGATATTAATCGACCCGCCAAGATGAGGTGGATCTTCATAACCAAGTATTTGCAAAGGTACTTCACTTATCTAGGTGTCGGCCCGTTTCTTGCAAAGTTTAGTGACCCAATCAGGTTTCCGCTTACGAAACACTTTGATAAACCGAGGCTACTGATTAAAGAGGGTAAAGAACCTGTCTCCTTTGAAGATCTTCCGAGGGCTCTAGCAAGAGGACCGTACAAAGACCCTAAGATACCGAGAGTCTACTTTTATTCTATGTTAGAGGGAGAGGAAGCAGTCTATAATAAGCAGTTCTATGGCGTTCTAAGGGACTTTGCGAAACGTAGGCTAGGCGTCTCCTTTCCTTCATCGGTGCTCCCTCTTAACAAGGATTTGGGCTCCGTTCGGATTTATCTTAAGAAATCTCTAGAGAAATATGGTAAGTTGGGTGGGGTCTGCCTTGGAATAGTTGAAGAGGAGTCTCCACTT**CAT**GACGAGCTC**ATA**ACGATGTTCGGAGAATACGGCTTGCCGGTT**CAGTGCGTTACA**AGTAAA**ACC**GCTATAGAGGACGTGTGTTTTTCTCGTAAT**GTC**TAT**ATA**ACGGAG**AAC**CTCTGTGCC**ATG**ATGATAGCC**AAA**GCAGGTGGCATCCCTTGGGTTCTTTACGACGAACTGAATTACGGTTGCTATGGTGCAATA**GAC**GTA**GGA**AGAACAAAGGCTGAATATTGGGCTATGGCTGTGGTTTACAACCGGAAGGGCATGTTTGAGACGCTTCCAGGGAGAATAACTATAGGGGAAAGGCTTGATGAGCACAGTATTAAGTATTGTGTTGAGGAGGCTGCGAGATACGCACGTGATAGTTCTGATTTTATTTTCTTTAGA**CAT**GGAAGAATTTATCCTAATGAAGTCGTAGCTTTCGAAAATGTAACAGGGGAAAGTCCCTTTAAGAACTGTGCCATTGTTGCCATAAAAACAGGGGAAGTCCCATATAGAATATTTCGTGAAGTTAATGGGAAAGTGATCAAACCTTTGTCTGGTGATTACTATCCCCTAGGACAGTCAACCGTTGTTGTATGCACCGTGGGTGTTGATGAGTACAAACATGGCATGCCCAAACCAATCACTATTGAAGTTATCCCTACAAAAGGGGAAGTTGAGGCCGTGAAGGTGGCCAGGGACGCGTTCTATTTAACGTACTTAAATTGGGGATCGCCGAGACAGAGCTATTCAATGCCGGCTCCTTTAAGGCTCGCT**CAC**ACTTTAGCTTCTGAATTAAGCGCTGGAATTCGAACAGGCGGGCGACCGTTTTAG

Translated protein sequence

MSLMANVFEANVRFDKSVANFDFFEVGGVESVSRRYELLGIVVTHLQREERKPFATYTPSDTENYVIGFLPNGPKKKQFGIEGLRVRYLGKDELPPSPPAYRTLTEILNKSKFWELKAYMWALGLHTLYPKRWKDLNEDYRDCGLCLFRGPYFRYNILPDGRITLTIDSKTHYVKSECFLDEIRRRGRDLKWFKEEMEAKREFYRRLGKKFRGIHFYYALAQQDIAINDIDTRPISQIPLKEEVKVGGRLCRTILEYLKAKYRDGPRVRAIDETQPGLKGDDLTFAPQFLHKSVDLEEVEDLILNEQTFYMDTRGVKGKRDINRPAKMRWIFITKYLQRYFTYLGVGPFLAKFSDPIRFPLTKHFDKPRLLIKEGKEPVSFEDLPRALARGPYKDPKIPRVYFYSMLEGEEAVYNKQFYGVLRDFAKRRLGVSFPSSVLPLNKDLGSVRIYLKKSLEKYGKLGGVCLGIVEEESPL**H**DEL**I**TMFGEYGLPV**QCVT**SK**T**AIEDVCFSRN**V**Y**I**TE**N**LCA**M**MIA**K**AGGIPWVLYDELNYGCYGAI**D**V**G**RTKAEYWAMAVVYNRKGMFETLPGRITIGERLDEHSIKYCVEEAARYARDSSDFIFFR**H**GRIYPNEVVAFENVTGESPFKNCAIVAIKTGEVPYRIFREVNGKVIKPLSGDYYPLGQSTVVVCTVGVDEYKHGMPKPITIEVIPTKGEVEAVKVARDAFYLTYLNWGSPRQSYSMPAPLRLA**H**TLASELSAGIRTGGRPF

Color annotation: The C-terminal Piwi_piwi-like_ProArk domain (in green) contains two subdomains, one of which provides the 5' RNA guide strand anchoring site (in purple) and the other, the catalytic site for slicing (in red). Conserved positions are printed in bold. Underlined positions mark the conserved [**H/R**]**KQ**TN**K** motif for short pAgos (Ryazansky et al. 2018).

BlastP search:

Comments:

cd04659: Piwi_piwi-like_ProArk: PIWI domain, Piwi-like subfamily found in Archaea and Bacteria. RNA silencing refers to a group of related gene-silencing mechanisms mediated by short RNA molecules, including siRNAs, miRNAs, and heterochromatin-related guide RNAs. The central component of the RNA-induced silencing complex (RISC) and related complexes is Argonaute. The PIWI domain is the C-terminal portion of Argonaute and consists of two subdomains, one of which provides the 5' anchoring of the guide RNA and the other, the catalytic site for slicing. This domain is also found in closely related proteins, including the Piwi subfamily, where it is believed to perform a crucial role in germline cells, via a similar mechanism.

**>12_bin.10.fna_01589 hypothetical protein**

Nucleotide sequence:

ATGAGACTAACAAGATTCCAGCCAACGATAAAACATCTTGAAGAACCACATATCGAAGTGGGAGAGGACATCGCTGTACCTGAGCCAAAAATGGGATGGACACTCTTGGGTCCTTTGGGGGAACGAACGCGACAATTTGAAATTAATCTTGGCTTAATCGGAGATAATGAGTCTTTAGAAAAAACGAGAAATTTAATCGAAAGACTAAATGTAACGACATATGGCAAAGACAAGGGCTTCTTACATGCCGATTTCCCTGGCTTAAACAAGCTCAGAATAAGATTCAATATTAAATGGGAAGCTGAAATAGATCAAAAATCGCTTAAACAAAAAATTGAAAGAACCACATCATTCACAAACAGAATTGAAACTGTTGGAAAAATAATTAAAGAAAAAATCAATGCATTGATGGATAGAGAGCCACAACCAGATTTATTGATATTAGCATATCCGAAGATTGTAGACGATTATTGTATAAAAGGCGCTATAGGAAGAAGGGGCGTGCCAAGAAAAACTTCTCTTGAGAAATCAATAGAAAAAGCAAGAGCCCTACACATGACACTTGACGGATTCCTGGGTATTCAAGCTCCTCCCAAAACCTATAGGCCCGTAGATCTA**AGG**AGTTTGGTC**AAA**GCTTACTGTATGGAACAAAATGTTCCTGTT**CAAATTCTTCGT**CCCCAC**ACA**ACAGAACCATATAATCCAGAAAAACCGAGAAGAGAGGATGACGCTACA**ACA**TTTTGG**AAT**CTTGTAGTT**GCA**TTGTTCTAC**AAG**GCAAATTATCTTCCATGGAGAGTGAAAGGGTTAATGGACGACACTTCATACATTGGAATA**TCT**TTC**TTT**CGGGACAAGGACGATCCAGCTAATGTCAAGACAGCACTAGCTCAATTTTTTTCATTAGATTCTGAGGGCTTTGTTTTTAAGGGCGATAAAGCATTAATCGATGAAAATAATGCCCCCCATGTTTCAGAAAGTGAATCAGAAAGATTAATGCGAAAAGCCATCGAAGTCTATGAGACAAATAAAGACAGGCGACCAAGAAGAGTTGTTGTTCATAAG**ACT**TCAAGATATACGGATGAAGAGATAGAAGGATTCAAAGCAGGTGCAGAAGGGATTCAAAGGCTCGATCTTCTGGCATTTGGTACGAGAAGTTTCAAATTAGTTAGATGGGGGCAACAGCCACCAATAAGAGGAACAATGGTAAGACTACCTGATAGAAGCATATTGCTATACACATATGGTTACATTCCGTATCTTAATGTATACCCTGGTCCTCGTGTACCATCTCCTCTTGAAATTTTGGAACATCATGGTACCACATCCATAGAAACGATATGTAAAGAAATACTTTCCCTTACTAAATTAAACTGG**AAT**TTTGCAAAATTCTGTATCAAAGCTCCCATAACAATTAGCTTTGCT**AAG**AGAGTTGGGAATATCTTAAGAAACGCACCGCCTGATATTGAGCCGCAGAATAAATTCAAGTTTTACATGTAG

Translated protein sequence:

MRLTRFQPTIKHLEEPHIEVGEDIAVPEPKMGWTLLGPLGERTRQFEINLGLIGDNESLEKTRNLIERLNVTTYGKDKGFLHADFPGLNKLRIRFNIKWEAEIDQKSLKQKIERTTSFTNRIETVGKIIKEKINALMDREPQPDLLILAYPKIVDDYCIKGAIGRRGVPRKTSLEKSIEKARALHMTLDGFLGIQAPPKTYRPVDL**R**SLV**K**AYCMEQNVPV**QILR**PH**T**TEPYNPEKPRREDDAT**T**FW**N**LVV**A**LFY**K**ANYLPWRVKGLMDDTSYIGI**S**F**F**RDKDDPANVKTALAQFFSLDSEGFVFKGDKALIDENNAPHVSESESERLMRKAIEVYETNKDRRPRRVVVHK**T**SRYTDEEIEGFKAGAEGIQRLDLLAFGTRSFKLVRWGQQPPIRGTMVRLPDRSILLYTYGYIPYLNVYPGPRVPSPLEILEHHGTTSIETICKEILSLTKLNW**N**FAKFCIKAPITISFA**K**RVGNILRNAPPDIEPQNKFKFYM*

Color annotation: The C-terminal Piwi_piwi-like_ProArk domain (in green) contains two subdomains, one of which provides the 5' RNA guide strand anchoring site (in purple) and the other, the catalytic site for slicing (in red). Conserved positions are marked in bold. Underlined positions mark the conserved [**H/R]KQ**TN**K** motif for short pAgos (Ryazansky et al. 2018).

BlastP search:

Comments:

cd04659: Piwi_piwi-like_ProArk: PIWI domain, Piwi-like subfamily found in Archaea and Bacteria. RNA silencing refers to a group of related gene-silencing mechanisms mediated by short RNA molecules, including siRNAs, miRNAs, and heterochromatin-related guide RNAs. The central component of the RNA-induced silencing complex (RISC) and related complexes is Argonaute. The PIWI domain is the C-terminal portion of Argonaute and consists of two subdomains, one of which provides the 5' anchoring of the guide RNA and the other, the catalytic site for slicing. This domain is also found in closely related proteins, including the Piwi subfamily, where it is believed to perform a crucial role in germline cells, via a similar mechanism.

**>12_bin.3.fna_01259 hypothetical protein**

Nucleotide sequence:

ATGAGTAGCGTAAAAATTTATGCTAGAATTTTGAAAGAATCAAACCTTTTATTTCGTGGCAATTTTCCTGCACCTGATCCCAAGGTTGGGCTTGCTTCATATGGGCCTTATTCTGCTACTAAAAACAGAATTCGAGTTGGGATAATAGGCGATAAGGAAACAATCTCTCAAACTCTATCTATGTTTGAAAAATTTAGACAACCTATCGAAGGGCCACTAAGATATCCGTTATGGACACCAGACTTTCCTGGCTTTTCAATAAATAGCCCATTCAAATGCGAATTCATCTTCTCAGAGCAATGGCAGCAGTCTATTAGTTCAGGTGATATCGCAAGGTTGGAGAATCTAACCTTTGATTGGGAACGAATTTCTAATTCTGTTGATATTTTCATTAAGCACCTTAAGAATCTGAGAGAAAAAGAAGAGAGTCCTGATGTTGTGATTTGTGTTCCCCCGAAGAATATAATGGATATTTGCATCGGTGCTCAGAAACGGCTCTGGCAGAGCCGTAAAGGAAGTGATTTTTCGAGAAGAAAGAAGCGAAGTTTTGATTAT**AGA**CAGCGGTTGCTGCTCGAATTTTTACCTGATTTTGAGCAAGCCTATGCCGATTTCCTTCAGAAGCGTGCCGCCGAGAACTTTCACCATTTGCTC**AAG**GCTAGAGCGATGAACCTTAAAATTCCGATA**CAGTTGATTCTG**CCTTAC**ACC**TTAGCAGCATTTCACAAGCTGGAGGAAAAGAGGATACAAGATGATGCTACT**GTG**GCTTGG**AAT**CTTTGCGTA**GCG**CTATTGTAC**AAG**GCCGAAACTAGATTATGGCGTATGGAAAATATTCAGGCCGGCACGTGTTATGCTGGCATT**TCA**TTT**TAT**CGTGAAAAGAGAATTCTGGGGGGCCGTATGGGAGTAAGTCTCGCTCAGGTCTTCACTCCGGAGGGAGAGGGTCTGGTTCTAAAAGGGGAGCGATTTGATTGGCCAAGAGGAAAGATACCTCACTTATCCCTAGAAAGCGCAAAAAGATTGATGGAGAAAGTTCTCTCCACTTATCAACGCTACACAGGTCAGTGGCCTGCGCGAGTTGTAGTTCACAAA**ACC**TCAACATTCGATGATAACGAAAAGAAAGGGTTTCAGCTAGCACTAGAGAGTGTCCCTAGGCATGATTTCATCGCTATATTGCAGAAAACGAGAAGGATCAAGTTCTTCCGGAAGGGAAAGGAGCCAGTCTTACGCGGCACAGTTATTGATCTTACAAATAATAATTGGCTTCTCTATACAAAGGCGTATATTCCATTCATGAGAGTCTATCCAGGTCCGAGAGTTCCTAGACCACTCGAGATAGTTCAACATGAAGGAGATACTCCAACAGATGGTGTCCTGAGTGAAATTCTTAGCCTTTCAAAACTGAATTGGAACAGTGCTGACTTCACAAGTTTCCTGCCGATAACATTGCAATTCTCA**CGA**CAGGTGGGGAGGATTCTTAGGGAATTGCCGCCTGGAATAACACCTGAGACGAAGTATCTCTACTATATGTAG

Translated protein sequence:

MSSVKIYARILKESNLLFRGNFPAPDPKVGLASYGPYSATKNRIRVGIIGDKETISQTLSMFEKFRQPIEGPLRYPLWTPDFPGFSINSPFKCEFIFSEQWQQSISSGDIARLENLTFDWERISNSVDIFIKHLKNLREKEESPDVVICVPPKNIMDICIGAQKRLWQSRKGSDFSRRKKRSFDYRQRLLLEFLPDFEQAYADFLQKRAAENF**H**HLL**K**ARAMNLKIPI**QLIL**PY**T**LAAFHKLEEKRIQDDAT**V**AW**N**LCV**A**LLY**K**AETRLWRMENIQAGTCYAGI**S**F**Y**REKRILGGRMGVSLAQVFTPEGEGLVLKGERFDWPRGKIPHLSLESAKRLMEKVLSTYQRYTGQWPARVVVHK**T**STFDDNEKKGFQLALESVPRHDFIAILQKTRRIKFFRKGKEPVLRGTVIDLTNNNWLLYTKAYIPFMRVYPGPRVPRPLEIVQHEGDTPTDGVLSEILSLSKLNW**N**SADFTSFLPITLQFS**R**QVGRILRELPPGITPETKYLYYM*

Color annotation: The C-terminal Piwi_piwi-like ProArk domain (in green) contains two subdomains, one of which provides the 5' RNA guide strand anchoring site (in purple) and the other, the catalytic site for slicing (in red). Conserved positions are marked in bold. Underlined positions mark the conserved **YKQ**TN**K** motif for long pAgos (Ryazansky et al. 2018).

BlastP search:

Comments:

cd04659: Piwi_piwi-like_ProArk: PIWI domain, Piwi-like subfamily found in Archaea and Bacteria. RNA silencing refers to a group of related gene-silencing mechanisms mediated by short RNA molecules, including siRNAs, miRNAs, and heterochromatin-related guide RNAs. The central component of the RNA-induced silencing complex (RISC) and related complexes is Argonaute. The PIWI domain is the C-terminal portion of Argonaute and consists of two subdomains, one of which provides the 5' anchoring of the guide RNA and the other, the catalytic site for slicing. This domain is also found in closely related proteins, including the Piwi subfamily, where it is believed to perform a crucial role in germline cells, via a similar mechanism.

**>12_bin.10.fna_01588 hypothetical protein**

Nucleotide sequence:
ATGGAACTAAACTCTGAGTTTGCTGAGGAATTAATTCATAAAGGTTTCGAATCGCCAGATCTTGATTATAAATTAAAATTTGACGGCTCTACTCATGCTTGGATGGAACTAGCTAAGGATATTTTTGGGATGGCGAATTATGGGGGAGGCTATATTGTATTCGGAGTTGAGGACGGAACATTTCGTCCAACTGGAATGAATATAGATTTCCATAAAGATGTGCAAGAATGGATAGATAGAGTTTCCAAATGGGCCACAGGGAAAATAAACATATCTTACATTGAACATATTGGAGAGATCGAGGGAGAGAGAAGAAAATTTCCTATCTTATGTGTTCATGCCTCCATAGGTTCATTCGTAATACCAAAAGTTGATGGTAAATACAAGTTGAAAAGTGGCGAAGAGAAAATTGCATTTAAAAAAGGCATTCTCTATATAAGAAGATCTACAAGTACAGTGGCTGCTTCAGGGAATGAGTATTGGGAATCTTTCTGGTCATTATTAAGGAGAACAGCAGAGAGAGTTGGCAGCGTCGGTACACCTCTCGAAGTAATATCGGCGTTGAGTAGAAAAGCCGAACCCGACAATGTAGAAGAGACACTTTGGTTTAACTTATTTCCCGTAGTTGAAATACCAGATTTAATTCATGCGGCAGATACAGAATTCAGATATCCTCGTGAAATATTCGAAACTATAGAGAATCGAGGAGATCAAAGTCAGCAGTATAACAAAATTCCACCTTTTTTGTTAGAGGACAAGAAAATATACTCTTTTTCACCATTTGACGAAAATAATCCGCTTAGTCAATGTACTGATCTGGCACACAGAACTTTATTTGACAAAGAAGAACATGCAACTATAACATCCATTCCAACAAAAGATTGGTTGAATGATGAAACTAAACACCAAAAATTGGTGAAATTGTTAAACTTCAATCTAAAGGACTTGTGTCACAAAAAAGGGTTTCATTATGACAATAGAAAGGATCGATATTATATAAGATATTTTGGAGGCCGTATTCCACAAATAACTTGGAAGCCATATCAAAAAACAAGCACAAGACCGCTCGTGTATCTCAGATTTAATGAAAAAACTGGTAGACTAATGTATTGTGAACATTTTGCAGGCAGATTAAGATTTACAATATTGGGAGAAGGAATCTATTTAGTTATAGAGCCCTTAAGAGTTTTAACATATGACGGAACGAATCCCTTAGATCAAAAGAGGAATACCAGAATTAGTACCAAAAAGAACTTCTTCTATCATAATAACAATTATCTGTACGACATGAAACTTTGGCTACATACTCTTGCAGGCAATAGAGAGGAAATTCACCTAGGTTACGGCTCAGGTAAAATTACAGTGGATGTAACATCCATCAATACTAAAGTAAACTTTGGCATATTTGATGATCAACATACAAGCGAAGATTTTCTTGATACCTTGAAATCAGAACCACTCGAATACGTCATAGAGTACGAAGAAGAGGAGGAATACAATCCTCTTACTGAAACACCATTGGAGGGATAG

Translated protein sequence:

MELNSEFAEELIHKGFESPDLDYKLKFDGSTHAWMELAKDIFGMANYGGGYIVFGVEDGTFRPTGMNIDFHKDVQEWIDRVSKWATGKINISYIEHIGEIEGERRKFPILCVHASIGSFVIPKVDGKYKLKSGEEKIAFKKGILYIRRSTSTVAASGNEYWESFWSLLRRTAERVGSVGTPLEVISALSRKAEPDNVEETLWFNLFPVVEIPDLIHAADTEFRYPREIFETIENRGDQSQQYNKIPPFLLEDKKIYSFSPFDENNPLSQCTDLAHRTLFDKEEHATITSIPTKDWLNDETKHQKLVKLLNFNLKDLCHKKGFHYDNRKDRYYIRYFGGRIPQITWKPYQKTSTRPLVYLRFNEKTGRLMYCEHFAGRLRFTILGEGIYLVIEPLRVLTYDGTNPLDQKRNTRISTKKNFFYHNNNYLYDMKLWLHTLAGNREEIHLGYGSGKITVDVTSINTKVNFGIFDDQHTSEDFLDTLKSEPLEYVIEYEEEEEYNPLTETPLEG*

Color annotation: The sequence marked in blue was identified as AlbA family DNA-binding domain-containing protein.

BlastP search:

Comments:

--- COG2865 predicted transcriptional regulator, contains HTH domain

--- AlbA family DNA-binding domain-containing protein is a putative nucleic acid-binding protein similar to vertebrate schlafen family proteins, which are associated with embryonic lethality, meiotic drive, immune processes, and orthopoxvirus virulence. More background about archaeal AlbA proteins from Jagadeesh & Vembar 2024 https://doi.org/10.1038/s41598-024-79937-4

--- Hid1 (high-temperature-induced dauer-formation protein 1), marked in italics, represents proteins of approximately 800 residues long and is conserved from fungi to humans. Functionally it might be involved in vesicle secretion or be an inter-cellular signalling protein or be a novel insulin receptor. It was previously thought to contain up to seven potential transmembrane domains separated by regions of low complexity. However, biochemical membrane fraction analysis demonstrate that HID-1 is a peripheral membrane protein tightly associated with the Golgi apparatus but not a transmembrane protein predicted by the bioinformatic programs. Furthermore, it contains a conserved N-terminal myristoylation site was required for HID-1 binding to the Golgi apparatus.

**>12_bin.3.fna_01260 hypothetical protein**

Nucleotide sequence:

ATGACAATTACAGAAGAGGTGCAAAAGGAATATGATGTATTTCTATCTTACCAGAGTTTGGACGAGGATTTTGCTAAAAAGTTAGGCGAGAAAATCCAATCAGTAACTTATAGAGGAAGAAGATTAAAGGTCTTCCTCGCTCCTTGGGATGTTCAACCTGGTGACAATATTGTTGTAAAATTGGATAAAGGACTAGCTAACGCTCGGTATTTTCTCCTGCTTTTGTCGCCGGAAGCTCTGAAGGCAGATTGGCCAACTGCTGAAAGGGCTGCCGCCATATACTCCGATCCCTCCGGTCGTTTGGGGAAGGTGATACCCGCTATAAGACGACCGTGTAAGCTACCTCCTTTACTTGCCTTTCGTAATTACCTAGATTTCCGACAGGACTCTAACTTCAATGCCGAATTCTTTAGACTTATCTCTAAATTGACAGGACAACCCCTACCGTCTGAAATCAATCCAAGTACTTTATCGTGGCATCAAGGCATTCGTCAAGAGAGTTCTCCCTTACAATCATTGATAGATTCATCTGAACCAGATGATGTTTATGAGAAGATATTTAGTAACCTATTCCCAGTTAGGAATCTGCCAGCTAGGGTATATAGCGCACCTACTAAATTTGTTGATAGAAAGCAGGTTCAAGTATATTTTCCACGAGATTCCATCGTTCCTCCTTTTATCCTGAAAGAGCATCGCCTTTTCACGTTTGCCAATCTTTCTGCAGAAACGCATCCATTTAAAGGTGTTGTCGAAGACTATGACATTCGGTCTGAACTAACCTCAGAATGGATACAAGATGAAAACCGTGCTCGCTGGTTGGTGGAGCTTTTAAACGATTGCATTCGTGTGCATCGATTTTCCTTAGGTTTGTACCTAGACAGGAGAGGAAAAAAATTATACTACCCAAAAGGTGTGCTTGCAAGCGAGAAGATAAACTGGAAGGCTCACCAGAGAACTGCAACTAGGCACCTTATTAAGGAGCGCAAGAATCCACAAGGACAGACCACATTTTTTCTCCACCGCGCTGTTGGCCTGAGATTCATCTTACTTGGGGTAAATGCATTCCTACGAATTGAAACAGGAAGAGTGTTCTCCATTGATGGAAGGCGATTGATTCGAGGTCGTAGAAAAACTGTTCTGTCGACCAGAGTCTTATCTCGTCAGAGAAATTTGGCAGAGTTTGATGATACAAGATTTTGGGCGTGGCTTCTTTCTGAAGATGGGTACACTATTAAGATGGATCTTGGTGGACTATATCTAGAAATTAACACTGAACCACTTTCTACAACTATCAGGGGAGGTATTTTTGGAGACTCCAAAATTTTACCTGAAATCGTTGACTCGCCACCTGACTTGCTGGAGCCAGAGGAAGAAGAAGTATTCGATGAAAAAGAATTCGAAGATCTAGATTACGAGGGTGAAGACTGA

Translated Protein sequence:

MTITEEVQKEYDVFLSYQSLDEDFAKKLGEKIQSVTYRGRRLKVFLAPWDVQPGDNIVVKLDKGLANARYFLLLLSPEALKADWPTAERAAAIYSDPSGRLGKVIPAIRRPCKLPPLLAFRNYLDFRQDSNFNAEFFRLISKLTGQPLPSEINPSTLSWHQGIRQESSPLQSLIDSSEPDDVYEKIFSNLFPVRNLPARVYSAPTKFVDRKQVQVYFPRDSIVPPFILKEHRLFTFANLSAETHPFKGVVEDYDIRSELTSEWIQDENRARWLVELLNDCIRVHRFSLGLYLDRRGKKLYYPKGVLASEKINWKAHQRTATRHLIKERKNPQGQTTFFLHRAVGLRFILLGVNAFLRIETGRVFSIDGRRLIRGRRKTVLSTRVLSRQRNLAEFDDTRFWAWLLSEDGYTIKMDLGGLYLEINTEPLSTTIRGGIFGDSKILPEIVDSPPDLLEPEEEEVFDEKEFEDLDYEGED*

Color annotation: The blue sequence is a TIR_2 domain, a family of Toll-like receptors

BlastP search:

Comments:

Pfam13676: TIR_2 domain, a family of Toll-like receptors.

Pfam01582. TIR domain. The Toll/interleukin-1 receptor (TIR) homology domain is an intracellular signaling domain found in MyD88, interleukin 1 receptor and the Toll receptor. It contains three highly-conserved regions, and mediates protein-protein interactions between the Toll-like receptors (TLRs) and signal-transduction components. TIR-like motifs are also found in plant proteins thought to be involved in resistance to disease. When activated, TIR domains recruit cytoplasmic adaptor proteins MyD88 and TOLLIP (Toll interacting protein). In turn, these associate with various kinases to set off signaling cascades.

SEFIR Superfamily: cl23749: Toll - interleukin 1 – resistance. This family comprises IL17 receptors (IL17Rs) and SEF proteins. The latter are feedback inhibitors of FGF signaling and are also thought to be receptors. Due to its similarity to the TIR domain (pfam01582), the SEFIR region is thought to be involved in homotypic interactions with other SEFIR/TIR-domain-containing proteins. Thus, SEFs and IL17Rs may be involved in TOLL/IL1R-like signaling pathways.

**>SRR22580798_bin.19.fna-00701 hypothetical protein; Aenigmatarchaeota**

Nucleotide sequence:

ATGCTAAGCAATAAGACTAAATCGCCTTGCGTTACATTTAAAACAAAAGATGGTACTTTCGTCGCACAGCCGGATGGTTATGACGAGTTACCTGATTCATTAAATTTAGTGAGAGTCGCCGTTAAGATAGAAAGGATACCTGAATTACAGGAGCTAAAGTTTGATTCACTTAATCCACAAACTGCCAGACTCGCAATAAGATTCCTGCAAGGGAGTATACAAAGAAAATTCTACGACAGTCCTTCGTTGTGGCAACCACGTTCAGGATATCCATTTTATCATAAATATCCAGACCAAAGGTTCAGACGATTGTCAAGAAATGTCGATATATTTAGAGGATTTGCTTTCAGAGTTGCACTTTTGCCCGATGGAAAAATTGGAATCTGCGTCGACGTAAGCAACAAATATGTCTCTCGACATCCTCTCCCAACCAAGATCGAGCGCGAAGACTCCCACAAGTATCAGGGAATGAACTGTTTATACGAATACGGAAACCGTTGGTATGAGATAAAAATTGGAGGTTTAAATGAGTTGAATGCAAGCGAGTTAATACTTCCTGATGGCACTTCTTTATTCGAACATGTTCATCGTAAAGCAGGTTCGCGAAAATCACCAAATCTTCTCGCTCTCCCAAGAGATTGTTCTGTCTTGATTTACTATAATACCTGGGGGGAGCAGAGAAATGCTCCTTCAGGATTATGCAGATTGACTTATGGAACAGTCCATCCTGATGTAGGGCGTTTTCATTCTAAAACAATCAAGGCGCCTCATATAAGAAGGGGAGAAATACAATTAGTTGTAGACAAATACTTTAGGGACTTGCCTTTTGGTTCTAATAAGATAAGTCTATCAGAAAAACCTCTAGTCGTAGAGACCAGAGGATTCATTATTCCGGACCTTGAATTTGGGAATAACAAAATTCTGTCACTACGAAATAGCCCTAATGCAACCCATGTCTCGTTAGATGAGTTTCCGTTTAAAAAGAAAGAACTTCTATATTCGCCGGAAGCTGGCTTATTCACAAAAAAGCCATTTGACCGTCAGTACCTTATCTTGCCAAAATCCGTCCATGAATCCTTCGGCAAAAAATTTATCGAGGATATGAAAAGAGAAGTTCAAAAGCTGTGTTCTGAAGAAGATATTTCCTATTCACCTATCTATGTTCTTTACAATGATTCTGTTCAAAAGTCAGTCTACATCTTGGGAAAGGAAATAATAAAGACAGTTGAGGAGAATGACGCTAAGCCGGGCTTTGGGATCGTTATGATACCCGAAGTAAAGTCAAAAAGAATGAGGAAAGAAGATGAGTTAGCAAACCTAGTGATGAGGGAATTACGTCAGAGAGGGATTTACGTTTCAATAATTCACACCACAGTACCGACCGAAAGTTATGAATATGTTTTATTTGAGGATGGAAAAGGAGAGTGGAGACTAGTCTCTGACCCAAGGCAAAAAAGTAAGTATAAAGGTTATTTGAAGAATGTTGTTTTGAACAAAATTTTGATTCTTAACTCTTATTGGCCTTTTGTTCTAAAAACACCGTTGAACGCAGATTTAATCATAGGCATT**GATGTAAAA**AACAACACCGCAGGTTTTACGGTCATTCATAAGAATGGTGCAGACATTACTTTTTATTCAAGTCAATCGAATCAAAGAGAACAATTGGATAGAAGCCAAATCCTTACAGAAATAAGAAGGATAATAACTAAAGAACAAAAACTATCGTTTAGAAACATTAGAAAGGTAGTAATCCACAGA**CAG**GGCAAGCTGTTTCCACCTGAAAAGGAAGGCATCATTCAAGCTTTGGACAAACTTGTAAAAACGGAACTTATATCCGCAGATCATCAATGCACTTTCGTAGAAATTAGAGCCACATCGAAAGTGCCCTTTAGACTATTCGAAGTTACACCTTCACCAGGCATTCAAAAGGAGTGGGTGGATAATCCGACAATTGGTACTTATGACTGTCTTTCAGAAAATGAAGCCTTTATTTGTAACACAGGTCCTCCTTTTGGACACAAAGGGACAACGAAACCTTTGCACATCATTAAAGATGGTCCAATGTCTATTGAATCTGTTCTCGAAGACGAATTCTATTTATCAAATCTAACGTGGACAAAAATCGATGATTGTTCTCGTCAACCTCTCTCTATAAAGATGACG**GAT**ATCAGGCTCAGAGAGTTCGCTGGAGATTATGACCGTGATGCTTTAAGATTTGGTAAGGAGGAGTGA

Translated protein sequence:

MLSNKTKSPCVTFKTKDGTFVAQPDGYDELPDSLNLVRVAVKIERIPELQELKFDSLNPQTARLAIRFLQGSIQRKFYDSPSLWQPRSGYPFYHKYPDQRFRRLSRNVDIFRGFAFRVALLPDGKIGICVDVSNKYVSRHPLPTKIEREDSHKYQGMNCLYEYGNRWYEIKIGGLNELNASELILPDGTSLFEHVHRKAGSRKSPNLLALPRDCSVLIYYNTWGEQRNAPSGLCRLTYGTVHPDVGRFHSKTIKAPHIRRGEIQLVVDKYFRDLPFGSNKISLSEKPLVVETRGFIIPDLEFGNNKILSLRNSPNATHVSLDEFPFKKKELLYSPEAGLFTKKPFDRQYLILPKSVHESFGKKFIEDMKREVQKLCSEEDISYSPIYVLYNDSVQKSVYILGKEIIKTVEENDAKPGFGIVMIPEVKSKRMRKEDELANLVMRELRQRGIYVSIIHTTVPTESYEYVLFEDGKGEWRLVSDPRQKSKYKGYLKNVVLNKILILNSYWPFVLKTPLNADLIIGI**DVK**NNTAGFTVIHKNGADITFYSSQSNQREQLDRSQILTEIRRIITKEQKLSFRNIRKVVIHR**Q**GKLFPPEKEGIIQALDKLVKTELISADHQCTFVEIRATSKVPFRLFEVTPSPGIQKEWVDNPTIGTYDCLSENEAFICNTGPPFGHKGTTKPLHIIKDGPMSIESVLEDEFYLSNLTWTKIDDCSRQPLSIKMT**D**IRLREFAGDYDRDALRFGKEE

Color annotation: The C-terminal PIWI domain (in green) contains the Piwi_piwi-like_ProArk: domain (in purple), here consisting of the active site for RNA slicing; the 5' RNA guide strand anchoring site seems to be missing. Conserved positions are marked in bold.

BlastP search:

**>SRR22580798_bin.29.fna_01460 hypothetical protein; Bathyarchaeia**

Nucleotide sequence:

ATGCCTTACGATGAGGTTCTGGAAATCTTAGATCCGCCTGTGCTGGAGTTTAAGGCTGGAAAAAAGGCAATTGACCCTGTAAGGGGTTTTGCCGTCCTTGGTTGTTCACCTTTAAAGGTACCTGATCTACGTTCAACTCCAAAAATTCGTGTTTTGATACCTAAAGGCTATGCAGAGTGCAAAGAAGTGATGACTATCCTCAACGACCTTAAAAAAGGGAAGATGACAGGAAAATATATCCGTTCTAGGTATAAACCGTTTCAGAAAGTATACGGATTAGATCTAGACATTGAGGAGCCAGAGGAATATGCCGATGAAAGTGAGATATACAAAAAGATAGATGAGGTCTCTAGGCTTCCGAATAGAAAAGCCCATGTATTTGTAATAGTCTCTCTGGCCAAATCGCCCTTAGGTGGTTTT**TAT**TACGCTGTG**AAA**ATCTCCTCTGTACAGAAGAGAGTTCAAACA**CAGATTATTCGT**AAGAAA**ACT**ATTGAAAACTATGTTAATCTAATGACTGAAGAGGAGAAAGGGGAT**TTT**CTATGG**AAC**TTTTCTCTC**AGT**ATTTTCTCA**AAG**CTTGGAGGGATACCTTGGAAATTGCGAGAAGTACTTAGCGGCGTCTCTGCATTCATTAGTGTAAACACAGTTGCA**TCC**TAT**GAG**GAAGGGATCACTAGAAGGGAAGGAGTAGTAGCCCTTGAAATAGCAAATAACTGGGGCGATCCTATAGGTAGATTCTTTGCCACGGGAATAGAAGTAAAGAAGAAAGAAGGAGCTATAGTTGTTGATATTGAGTCGATAAGTACTCTCATGAAGAAAGCCCTCGATCGAGTCGAGAAAGAAATTGCAGATCCTGAAAAATCCAAAGAAAAAGACTATGTAGTAGTCCATATT**AAG**GATAGATACGCCGACTCAGTTTACGATTCAATAGCAAAAACAATAATTTCGAAAGGATTTAAGAAATTCAAGATAATTCACGTTCAGAAGGAAGGCAGTCTGAGGCTTTACGATCCCTCAAAAAGGATAACTCGAGCTTGGCCAAAAGAAGGCAGTTATTGGTACCTTGAGAGAGGCAAAATAGCGTTTCTATTTACACTTGGGAGATGGCAATACTCAATTTCACCCAGAAGTGAGCCGTATGTCATTGGAGCTCGTAATGTTTCTCCCCTGCAAACAAATTTTGTAAAAGGAAATGAGGGTTCTAATCTAACTGTAGATGACCTTCGCCATATATATTACCTTACTAGACTTCACTACTATTCTGCGGACATCCCAAGAATCAAAATGCCTTCTACAATAAGGTTAGGA**TCG**AAAGCAGCACATCTAGCAGCATGTGGACTCACTAAATCCGACTTTGACATTTTTTATCTATACTAG

Translated protein sequence:

MPYDEVLEILDPPVLEFKAGKKAIDPVRGFAVLGCSPLKVPDLRSTPKIRVLIPKGYAECKEVMTILNDLKKGKMTGKYIRSRYKPFQKVYGLDLDIEEPEEYADESEIYKKIDEVSRLPNRKAHVFVIVSLAKSPLGGF**Y**YAV**K**ISSVQKRVQT**QIIR**KK**T**IENYVNLMTEEEKGD**F**LW**N**FSL**S**IFS**K**LGGIPWKLREVLSGVSAFISVNTVA**S**Y**E**EGITRREGVVALEIANNWGDPIGRFFATGIEVKKKEGAIVVDIESISTLMKKALDRVEKEIADPEKSKEKDYVVVHI**K**DRYADSVYDSIAKTIISKGFKKFKIIHVQKEGSLRLYDPSKRITRAWPKEGSYWYLERGKIAFLFTLGRWQYSISPRSEPYVIGARNVSPLQTNFVKGNEGSNLTVDDLRHIYYLTRLHYYSADIPRIKMPSTIRLG**S**KAAHLAACGLTKSDFDIFYLY

Color annotation: The C-terminal PIWI_piwi-like ProArk domain (in green) consists of two subdomains, one of which provides the 5' RNA guide strand anchoring site (in purple) and the active catalytic site for slicing (in red). Conserved positions are marked in bold. Underlined positions mark the conserved **YKQ**TN**K** motif for long pAgos (Ryazansky et al. 2018).

BlastP search:

**>SRR22580803_bin.1.fna-_00806 hypothetical protein; Bathyarchaeia**

Nucleotide sequence:

TTGCCTAAGAATTATGAATTAAACACATTTATCTCGGTTGATCCAACTCTTAAAGACGGACTCGAGTTAGATTTCTTTTCAGTCGGCGAACTTCCACTCGGTCAGAAAAGATACAGAACGCTACGCTCCCTTTCACGGGAGATTTCAGGGTCATATAAGATAACCCATGTAACGTGGTACATTCCCAGCTTGAAGGAAAACTATTTCGTTACCTTGCCAGGAGACATAAAAGAGAAAGAGATCACTACTGCAAACTACACGATCACATACGTGGAAACTAAACCACTTTTACCCCTTTCTCTATCTTCGAGAAATACACTTGATCGCCTCCTCTATCAATCAGTTCGCCGAGCATTGTCAAAGCAGAGATTCTGGAAGAAGTACTATAACGTATACATGAAAGACGAGCCGCTTGTTACCTCACCTGAATGCGAAATCTTCACAGGTTTCTATTTTAGAATAGACGTTCTCAGCAATGGGACAGTTGGTTTGACCATAGATCCCACAACTACCATAATAAGTTCATTCACCGTGTGGGACATGATGCAGGAGAAAAGCATTGAGGAGGTTCGTAGGTGGATCCGAGGCAGTCCAAAAGAGCCAAGGCGAAGAGCTGGAGTAAACGTCTTCGAGCATAGGGCTAAGCGGGTTAGCCGTTTAATCGGTTTCGATCCGGAGAAAAAGGTGTGCCACCCCTCTTTTCTGGATCCTTATACAAACAAGATAACAAGCGTAATCGAATACCAAAGGAGGCGATACGGTATTTCTGGCATAGAGCCATCAGAACCCATAGCCCTTATCGAGTATAGTGAAGCCTTTCCTCCCGCGCACCACGCACCTAGTTTGTTGAAGCCCACGTTAACAACAGAGGAAATACCACCTGAGTTACAAAAGAGATATATTTTCTTACCACCACGCCAAAGAAGAAAGTTAACCTTAGGATACAGGCGCTACCTAGAAGAACTAGCGATCGGGGAAAAGCGAGTTTTGTTTAAGGATCATATGGCAAATCAAAGGGATATCCCTTTCAGCAACATTCTGATGCCCACCCTAGTTTTTGGGAATACCCAGAGGGTTAAAATCAGCGATTATAAAGACCTGAAAACCATCAAGAAGAAGAGCTTAGAAATGCACGGCCCATACGAGAAGCCACCTATAATGGAAACATGCTTGGTGTACAGATCCGACTTTGACCGGAATCTTATTCTTAAGTTCTATCGCGATTTGCAAAAGTATATGAAGAGATACTATCACGTTAATCTTTCTAACAGACCGAGAGAGATACGAACAAGGGACTACTACGATCTTTCAGAAAAAATCAGAGAACATAGAGATAAGATGAAAGTCTCATTTTTCCTTGTCGTTTTTCCATCTACAGAGGCGGTGGAG**TAC**GACTACATC**AAG**AGTGAACTGCCAGCTCCTTCC**CAAGGCGTGGCT**ATAGAT**AGC**ATACAGCTTAAGGAAGAAATTCAAAAAACGAAAGACTATGAAAGGAAGAATGAGCTTAGAAGC**ACA**TATTAC**GGA**ATAGTGTTTTTTGTCGCCATG**GGC**ATGTTCATA**CAA**GCAGGTGGTATACCGTGGGTTTTAGGAGACAAGCTTAATGCACACTGCCACATAGGCATC**GAT**GTA**GGC**GGAAAGGAAGCTCGTGTGGCATGCTATGGATACCTCTTCGACCAGATCGGAAGGGTTGTCGGAACTGAAGTGGGTACCGCCCAAAGCGGGGAAGTGGTGGAAAGCGGTCGGATAAAGAGAGCCATCATAAAGCTACTATCAAAGATAAAGCCCAAACAAGGGACAAGGATCATCATAACTAGA**GAT**GGAAGATTGTTGGAAGAGGAGAAGCAGGGCATAAAAGAAGCAGTAGAAGAGTACTTATCCCCAGAAATTACCAAAAGTCTTGGCCTCGCAGTCATAGAAATACGGAAGAATGTCCCGTTTCGTATCTACGACATCGTTGATCAGTGGGTTAAAAATCCGCTGATAGGCAGATATGCCCTCATAGATGAAAACACGGCCGTTCTCTGCACAACAGGCTATCCATTAATTACCCAGGGAGTTGCCGAACCCTTGCTAATATACGCCCACAACATGTACAGAGAATTCAAAATACGTGAGATACTTCAAGACATTTTGTGCCTCTCAGAACTGAACTGGATAGCTGGAGATAAGCCATCCAAATTACCGGTAACAATCAGTCTTGCA**GAA**GCCCGTGCCTTATTTGCTGAAAAGGGCATTACACCTGCTTCGAGGCTCCCTGTCTAA

Translated Protein sequence:

MPKNYELNTFISVDPTLKDGLELDFFSVGELPLGQKRYRTLRSLSREISGSYKITHVTWYIPSLKENYFVTLPGDIKEKEITTANYTITYVETKPLLPLSLSSRNTLDRLLYQSVRRALSKQRFWKKYYNVYMKDEPLVTSPECEIFTGFYFRIDVLSNGTVGLTIDPTTTIISSFTVWDMMQEKSIEEVRRWIRGSPKEPRRRAGVNVFEHRAKRVSRLIGFDPEKKVCHPSFLDPYTNKITSVIEYQRRRYGISGIEPSEPIALIEYSEAFPPAHHAPSLLKPTLTTEEIPPELQKRYIFLPPRQRRKLTLGYRRYLEELAIGEKRVLFKDHMANQRDIPFSNILMPTLVFGNTQRVKISDYKDLKTIKKKSLEMHGPYEKPPIMETCLVYRSDFDRNLILKFYRDLQKYMKRYYHVNLSNRPREIRTRDYYDLSEKIREHRDKMKVSFFLVVFPSTEAVE**Y**DYI**K**SELPAPS**QGVA**ID**S**IQLKEEIQKTKDYERKNELRS**T**YY**G**IVFFVAM**G**MFI**Q**AGGIPWVLGDKLNAHCHIGI**D**V**G**GKEARVACYGYLFDQIGRVVGTEVGTAQSGEVVESGRIKRAIIKLLSKIKPKQGTRIIITR**D**GRLLEEEKQGIKEAVEEYLSPEITKSLGLAVIEIRKNVPFRIYDIVDQWVKNPLIGRYALIDENTAVLCTTGYPLITQGVAEPLLIYAHNMYREFKIREILQDILCLSELNWIAGDKPSKLPVTISLA**E**ARALFAEKGITPASRLPV

Color annotation: The C-terminal PIWI_piwi-like ProArk domain (in green) consists of two subdomains, one of which provides the 5' RNA guide strand anchoring site (in purple) and the other, the active site [catalytic site for slicing] (in red). Conserved positions are marked in bold. Underlined positions mark the first three positions of the conserved **YKQ**TN**K** motif for long pAgos (Ryazansky et al. 2018).

**PIWI domain Alignment (PAUP file) used for phylogenetic analysis**

```
[Name: fna01589          Len:   353  Check: 82D5F173]
[Name: MFC1803759        Len:   353  Check: DDDF3E15]
[Name: MDR2719462        Len:   353  Check: 78FA39F8]
[Name: TBR08567          Len:   353  Check: C7560F32]
[Name: MFY93000140       Len:   353  Check:  F382C5E]
[Name: MCL5876754        Len:   353  Check: 20F338AD]
[Name: HET6574466        Len:   353  Check: F34BF912]
[Name: WP413431139       Len:   353  Check: 9ECFA289]
[Name: TXG99657          Len:   353  Check: 9CD6B4E0]
[Name: MCG3129241        Len:   353  Check: D30CD0F8]
[Name: MCE9652100        Len:   353  Check: 4C963D31]
[Name: CUR52642          Len:   353  Check: 7060E355]
[Name: HWY27817          Len:   353  Check: 372EEF4A]
[Name: MEM5867970        Len:   353  Check: 11FF8E97]
[Name: MEM0117216        Len:   353  Check: 3FC85758]
[Name: MFN8792580        Len:   353  Check:  12A6DB5]
[Name: HEY2956445        Len:   353  Check: E1477EC8]
[Name: TKJ43753          Len:   353  Check: 2BA26E2E]
[Name: fna01259          Len:   353  Check: 68A49E40]
[Name: MBI4232046        Len:   353  Check:  E6BF163]
[Name: fna00701          Len:   353  Check: 88239AE4]
[Name: MEM2112053        Len:   353  Check: FCEB093D]
[Name: MGA2385153        Len:   353  Check: 6CCD14FB]
[Name: MFZ3168249        Len:   353  Check: D8791EB7]
[Name: NVM53154          Len:   353  Check: F6DF5F54]
[Name: MGI0086902        Len:   353  Check: EA3A907B]
[Name: RLI97245          Len:   353  Check: DAE8843A]
[Name: HUV01944          Len:   353  Check: 4BBCAE3E]
[Name: KAF5432662        Len:   353  Check: 1E130AA8]
[Name: MGA3291213        Len:   353  Check: A0E2D588]
[Name: MBT0158967        Len:   353  Check: 94886C52]
[Name: bin00806          Len:   353  Check: 61EFA9E9]
[Name: fna01190          Len:   353  Check: F0F895B1]
[Name: fna01717          Len:   353  Check: F0F895B1]
[Name: JAHPYW101         Len:   353  Check: EDA7AD6D]
[Name: Meg22Bin182       Len:   353  Check: 23621BFF]
[Name: D4993C5H2         Len:   353  Check: 23621BFF]
[Name: RLG97969          Len:   353  Check: 11F41E5C]
[Name: MCD6238362        Len:   353  Check: 79C61B78]
[Name: MGH5779747        Len:   353  Check: CF37C746]
[Name: MDO8724606        Len:   353  Check: C4026218]
[Name: MCL5732256        Len:   353  Check: 25880B06]
[Name: bin01460          Len:   353  Check: AD4E981A]
[Name: MCD6446422        Len:   353  Check: 6C3D92D1]
[Name: MCD6241707        Len:   353  Check: 965CB06C]
[Name: MGB9959866        Len:   353  Check: 6CBEDC75]
[Name: HrAgo1            Len:   353  Check: 74FE7961]


begin data;
 dimensions ntax=47 nchar=353;
 format datatype=protein interleave missing=. gap=-;
  matrix
 fna01589  -RSLVKAYCMEQNVPVQILR PHT-TEPYNPEKP-----RR EDDA-TTFWNLVVALFYK-- -------------ANYLPWR VKGLMDD--TSYIGI-----
MFC180375  -RSLVKSYCMEYDIPIQILR PHT-YEPYNPDRP-----RR EDDA-TTFWNLAIAMFYK-- -------------ANNLPWR VKGLMED--TSYIGI-----
MDR271946  -RSLVKAICMDYDIPVQILR PST-TEPYNLEHP-----IR EDDA-TTFWNLVVAAFYK-- -------------SNHLPWR VKGILND--TCYIGI-----
 TBR08567  -RSIVKAICMQHNIPVQIIR PQT-TEPYNPEKP-----RR EDDA-TTFWNLVVAMFYK-- -------------SNNIPWQ VRGLMQD--TCYIGI-----
MFY930001  -RSVIKSICMKHDVPIQIIR PHT-TEPYNPDKP-----KR EDDA-TTFWNLVVAIFYK-- -------------ANNIPWK VKGLMQD--TCYVGI-----
MCL587675  -RSMIKIICMKYKIPIQILR PHT-TSPYNSEVP-----NR EDDA-TTYWNLIVALFYK-- -------------SNRLPWR VRKLMAD--TCYVGV-----
HET657446  -RNALKAHAMRAGLATQLIW EST-LDGSR---------RL QDPA-TVAWNFFTALYFK-- -------------AENMPWE LRFSSPR--TCFVGV-----
WP4134311  -HNSVKAHAMKSKLATQLVW YST-LTRSR---------GK EDPA-TTAWNFFTALYYK-- -------------AGNVPWR LKLGTQQ--TCYVGI-----
 TXG99657  -HNSIKAYAMRSGLATQLVW HST-LARSR---------GK EDPA-TTAWNFFTALYYK-- -------------SGNVPWR LNFGLAR--TCYVGI-----
MCG312924  -HNALKVHAMRSQLPTQLIW EST-LAGTR---------DT QDPA-TIAWNFFTALFYK-- -------------AGNVPWE LGFEAPG--TCFVGV-----
MCE965210  -WRALKAGSMSFGMPTQIVW PST-TKPKK----ENEKQDR QDPA-TIAWNFSVALHYK-- -------------GSGFPWT MTRMKQG--TCYVGI-----
 CUR52642  -WRALKAGGMAFGMPTQIVW PST-TQPKK----IDGKQNR QDLA-TIAWNFSVALHYK-- -------------GSGFPWT MTRMKIG--TCYVGI-----
 HWY27817  -WRSLKALAMQIPMPTQIMW PRT-LAQ--------RGLTK QDDA-AVAWNACVGIYYK-- -------------GSGFPWT ITKMQQG--TCYVGV-----
MEM586797  -RCLIKAKAMEMGIPTQLVK PRT-LLS------YPKDVML QDEA-TRAWNFSVALYYK-- -------------AEGYPWK LTEMTVG--TCYVGI-----
MEM011721  -RRILKADAMEIGIPTQIAW PGT-FTGE-----QASGRMV QDET-TRAWNFSVALYYK-- -------------GGGFPWR LAEARQG--TCYVGV-----
MFN879258  -RRGLKAEAMRFGIPTQIVW PRA-IELVT-QIGGRGTKKA QDVA-TRAWNFTTALYHK-- -------------AGGSPWR LAKLEPG--VCYVGI-----
HEY295644  -RRGIKAETMQLGIPTQLVW PLT-LSLTD-QEPLPGQRRP QDIA-TRAWNLTTALYHK-- -------------AGAAPWR LAEIEPG--VCFVGV-----
 TKJ43753  -RRGLKARAMQYGIPTQLIW PRT-IKLGGTQKRGFRERVL QDDA-TRAWNFVVALYHK-- -------------AKGIPWR LANIKPG--VCFVGI-----
 fna01259  -HHLLKARAMNLKIPIQLIL PYT-LAAF----HKLEEKRI QDDA-TVAWNLCVALLYK-- -------------AETRLWR MENIQAG--TCYAGI-----
MBI423204  -HDYLKSRAMDVGIPTQFIR PDT-LENY---VTAASGKK- QSET-HFAWNLCTALYYK-- -------------GQGQPWK LANAPDG--TCFIGV-----
 fna00701  -ELANLVMRELRQRGIYVSI IHT-TVPTESYEYVLFEDGK GEWR--LVSDPRQKSKYK-G YLKNVVLNKILILNSYWPFV LKTPLNA--DLIIGI-----
MEM211205  -------------------- -------------------- ------LVYDRKQIGKYK-G YLRNVVLNKILILNSFWPFV LKTPLNA--DLTIGI-----
MGA238515  -ELANIVMRLLKERELYVSI IHS-RVSTESYEYGTTDNGR EDWR--LVFDRRQQGKYR-G YIKNVVLNKVLILNSLWPFA LNTPLNA--DLTIGI-----
MFZ316824  -ELGNLVMKELRKREIFVSI IHT-ATAEESFEDTSLDGKE SNWE--LVTDGRQQGKYK-G YLKNVALNKILLLNSHWPFV LKTPLNA--DLTIGI-----
 NVM53154  -ELGNLLMSKLRERDIFASI IHT-STVEASFEDISNDGEE SKWV--LVSDRKQKGIFK-G YLKNVVLNKILLLNSYWPFG LKTPLNA--DLTIGI-----
MGI008690  -ELGNLVMRQLRQRGVYVSI MHT-EVPSRSYTKVSTSAGS N-WQ--LMDDQKIASRFK-G YLSNVILNKIMILNSCWPFV LDTQLHA--DLIIGI-----
 RLI97245  -EIANAVIRKLRKRDIIAAV IHT-GVSSNSYEQSNGQ--- --WR--VTFDRKRQGRLK-G YLRNVVLNKILILNHFWPFV LKTPLHA--DLTIGI-----
 HUV01944  -GSYFYLKKVLKNIPLQCYR SSS--LKTRGEVLRKYKN-- ------MIFVTAVGFAAK-- -------------MGFKPWV LQTQLSA--DCYIGL-----
KAF543266  -KSYFYLKKVLRDIPLQCFQ VTS--LETKRGNEKRYDN-- ------MIFVTAVGFGAK-- -------------MGFRPWV LKTQLSA--DCYIGL-----
MGA329121  -TNQYLNFKEWFDKPNQVLT LRV-INERYQARGGRYQN-- ------LLLNVCAGLLGK-- -------------MGGRPWV LEKKLSA--DFYVGL-----
MBT015896  -TTRYLNFKEWFDKPNQVLT YRV-IDERYQLRRGKYQN-- ------LLLNVCAGLLGK-- -------------MGGRPWI LDKKLSA--DFYVGL-----
 bin00806  -AVEYDYIKSELPAPSQGVA IDS-IQLKEEIQKTKDYERK NELRSTYYGIVFFVAMGMFI Q------------AGGIPWV LGDKLNA--HCHIGI-----
 fna01190  -HDELITMFGEYGLPVQCVT SKT-----AIEDVCFSRN-- ----VYITENLCAMMIAK-- -------------AGGIPWV LYDELNY--GCYGAI-----
 fna01717  -HDELITMFGEYGLPVQCVT SKT-----AIEDVCFSRN-- ----VYITENLCAMMIAK-- -------------AGGIPWV LYDELNY--GCYGAI-----
JAHPYW101  -HDEITNICGRLGVPSKCIA EST-----VEMVVNRKKS-- -----FPLAGYVSSLLTR-- -------------AKCIPWV LATQLSY--DCYIAT-----
Meg22Bin1  -HDRITNICGRIGVPSKCIM EHT-----VNTVVNRRKW-- -----FPLAGFVSSLLTR-- -------------AKCIPWV LDTQLSY--DCYIAT-----
D4993C5H2  -HDRITNICGRIGVPSKCIM EHT-----VNTVVNRRKW-- -----FPLAGFVSSLLTR-- -------------AKCIPWV LDTQLSY--DCYIAT-----
 RLG97969  -YDELTNTAGILGIPIKCVN QEN-----AKSICGGSKI-- -----NYLENLIASIITR-- -------------AGGIPWV LYDKLNY--DLYVAV-----
MCD623836  -HDMLNNLFGRFDIPVQCVE HTT-----ATNIVNYNLY-- -----SFLEGICAGIFAK-- -------------SEGIPWL LYDKLNY--DRYAAV-----
MGH577974  -YKTLNNLFAQYGIAVQCLR KKT-----AVSIIDYQRK-- -----GFLEGICAGIFAK-- -------------AGGIPWL LYDPLVY--ERYAAI-----
MDO872460  -HTELNQIFGNYKVPLQCVT EST-----ATAIINGKYQ-- -----YRLEGICAGLFVK-- -------------SGGIPWL LQSPLNY--DRYIAV-----
MCL573225  -ACGRLEIANKSIREESCIK VVK---------NSRQKN-- -----GILLNTLFPIFAR-- -------------SGSVPWI ISTRLSY--AGYVFV-----
 bin01460  -YYAVKISSVQKRVQTQIIR KKT---IENYVNLMTEEEKG -----DFLWNFSLSIFSK-- -------------LGGIPWK LREVLSG-VSAFISVNTVA-
MCD644642  -YYNVKTSSIMKEVQTQIIL KRT---IKNYFQKMTEEEKG -----DFLWNFSLSVFAK-- -------------LGGIPWK LNEVLSG-VAAFISLNTVS-
MCD624170  -YYKIKGKLI-NDVRTQIII GRTYEKYYRTRQDFKKNPFK NREYGDFLWNLSLSIFTK-- -------------LGGIPWK LKSPIEG-VLAFIGFASEF-
MGB995986  -YFKVKAYAIVNKIREQIII KPT---VDKAFRYITQNDIL KYN--DLVWNTALSIFTK-- -------------LGGIPWK LKECMSP-IDTFLAISTIM-
   HrAgo1  YRKIKQLCYRDLGIANQNVV LKN------LRDQKRRMPII RNLVRQIICK-VPNFNTK-- -------------YGGALWK IKNNSIPDKTLIVGI-----

 fna01589  SFFRDKDDP-ANVKTALAQF FSLDSEGFVFKGDKALIDEN N-APHVSESESERLMRKAIE VYETNKDRR--P------RR VVVHKTSRYTDEEIEGFKAG
MFC180375  SFFRDRTDP-SNVKTALAQF FSLDSEGFVFKGEKAKVDEN N-AAHVSKDDAIDIIQTAIK VYESNKGHK--P------RR LVVHKTSRFNEEEIEGFREG
MDR271946  SFFRDRDDS-SVVKTSLAQF FSLDSEGYVFKGKKAIIDEY N-APHVSKEEARELIERAIE VYKANKGDCS-P------RR IVVHKTSRFTEDEKIGFQEG
 TBR08567  SFFRDRGDS-AMVRTALAQM FSLDTEGYVLKGNKATTDEN N-AAHISKNDATSLIQQAID VYKRNRNGQP-P------SR IVIHKTSRFDDGEQAGFAEG
MFY930001  SFFRDRGDV-STVKTALAQV FSLDTEGFVLKGKKATVDEN N-SPHISQNDASSLIQQAID VYKRNNEGQL-P------KR VVIHKTSRFDENERNGFTEG
MCL587675  SFFRDRSDP-ASVKTALSQV FSLDSEGYVFKGGKAITDEN Y-TLHVTKEEASSIITQAIT QYKSSKNDQL-P------RR VVVHKTSKYESYEIEGFKAG
HET657446  SFYRESDDPNSPTRTALAQA FSESGEGLVLRGERVIWDRD R-DRRPHLSREAAERLLREA LTLYAQHFGDPP------KR VVLHKTSRYWLDELNGFRAG
WP4134311  TFYRESADPDAPTRTCLAQA FSENGEGLVLRGERVAWDQK R-DKKPHLTGHDAEAILRRV LQLYEAHVDDEP------QR VVLHKTSRYWAEELEGFKAA
 TXG99657  TFYRESADPDAPTRTCLAQA FSESGEGLVLRGEQVTWDTD R-DKKPHLNRNDAEKLLRRV LELYRQHFDEKP------NR VVLHKTSRYWPDELEGFKAA
MCG312924  TFYRERPGASAVTRVSLAQV FSETGDGLVLRGEPVEWDRD R-DRKPHLSEPAA-RKLLEH AIQQYEKHFNKP------RR VVVHKTSRYWPEELAGFKAG
MCE965210  TFFKDLTDDENRMRTSMAQV FTYTGEGLVIRGDRFEWNTF Q-DRSPHLDEKGAEELLKKA IELYTNRIGQSP------LR VVVHKSSKYWDDEKKGFENA
 CUR52642  TFFKDLTDDENRMRTSMAQV FTYTGEGLVIRGDRFEWDKH Q-DRSPHLNEKGAEELLKKA IELYTNRIGQPP------LR VVVHKSSKYWEDEKRGFESA
 HWY27817  SFFRNLADSQNRMSTSLAQI FTYTGEGLILRGDSFEWSTQ L-DRSPHLTQDSAEKLLSKA IGLYRQKMGQPP------TR VVIHKSSQYWDDEKSGFSKA
MEM586797  TFYKDLTNPEGRMRTSMAQV FTHTGEGLVLRGGRAVLDE- --TKSPHLSEVGAYQLMKDI LQLYHEQMGQLP------SR LVVHKSSRFWSDEISGFNKA
MEM011721  SFYKERGSVGERTRTSLAQI FTHTGEGLVLRGGRFAWDEK R-GKSPHLSEDSAFQLLSEA LNLYQRQMGAPP------LR IVVHKSSRYWQDELAGFRKA
MFN879258  SFYRESGAKGSQLRTSMAQA FTSAGDGYVLRGNPIPWNED TQGRSPHMDKKSAAALLNDV LELYKKQSRGSLP-----NR LVIHKTSRFSEDEISGFDSV
HEY295644  SFYRDHSGAAPTLRTSMAQA FTAAGDGYVLRGNSFEWNVA KQGPSPHLDAKNAAALMRGV LELYQRQNRGSFP-----TR MVVHKSSRYWDDELAGFRES
 TKJ43753  SFFKERAAEYPRMCTSMAQT FSFAGEGYVLRGKSFEW--T K-DVSPHLRYQPAKELMNEV IELYKTQNKGLP------RR VVVHKSSRFWEDEINGFHVA
 fna01259  SFYREKRILGGRMGVSLAQV FTPEGEGLVLKGERFDW--P R-GKIPHLSLESAKRLMEKV LSTYQRYTGQWP------AR VVVHKTSTFDDNEKKGFQLA
MBI423204  SFYKEKKQLGNNMGACLAQV FPPEGDGIVLRGERFKW--K Q-FEEPHLDAAAAQRLVERA ISAYETEHRLKP------KR VVIHKTSRFNEREKIGFQAG
 fna00701  DVKNNTAGFTVIHKNGADIT FYSSQSNQREQLDRSQILTE ---------------IRRII TKEQKLSFRNI-------RK VVIHRQGKLFPPEKEGIIQA
MEM211205  DVKNNMAGFTVIHKNGADIT FYFSESDQKEQLRASQVCTK ---------------IIEII SNEQKLLPRNI-------RD IVIHRQGKLFPQEKDGIVQA
MGA238515  DVKNDMAGFVVVHKNGADIA FYSSTSTQKERLEKKHVCSK ---------------LFEII SNEQKLLPSAI-------KN IVIQRQGRLFSCEKAGIEQA
MFZ316824  DVKNNTAGFTLIYKSGAEIR FFESVTEQKEQLTKSHIYQK ---------------ILEIM REEKGLVPHKI-------TS IVVHRDGIFYPSEREGIRKA
 NVM53154  DVKNNIAGFTLIYKDGSKIR FEHSESNQKEQLNKNQMKSK ---------------ILEIM RDEDIESPEDI-------KN IVIHRDGTLYRTEIEGIKLA
MGI008690  DVKNNTAGFTFVYRDQT-IR FFHSDSEQREQLSKRQLETK ---------------IYEFV KDEQERKKHEI-------KN ITIHRDGRIFLNEIDGIKQA
 RLI97245  DVKGNIAGFTLVYKSGEQIR FFPSESDDKERLGKAHIKAR ---------------VEEIL KEESATIPLKTAI-----KK IVIQRQGRLFYPEQAGITQA
 HUV01944  DVGGDRSHVMGNCYIIDKSG TYMEIGTIPQRGEAISEDTM ---------------VEIVS DAIFDSIRKYKDESARI-KS IVIHRDGEIRKSEKNGISDA
KAF543266  DVGGDRTHVMGNCYIINKQG TYLELGAIPQRGESISEDTM ---------------SEVVS DAIFDSIKKYKDVMGRI-ES IAIHRDGQITKSEKSGISDA
MGA329121  DVGGDKNARVACYTFFDGYG NYVREEWRPQRAEEINPQEL ---------------KRIVV NALATYGNPVN--------S VVFHRDGQFTDGELQGMAAI
MBT015896  DVGGDKNARVACYTFFDGYG NYVREEWRPQRAEEINTQEL ---------------KRIVA NAIAGYGNPLN--------N IVFHRDGQFTDGELQGIDAL
 bin00806  DVGG-KEARVACYGYLFDQI GRVVGTEVGTAQSGEVVESG ---------------RIKRA IIKLLSKIKPKQGT-----R IIITRDGRLLEEEKQGIKEA
 fna01190  DVGRTKAEYWAMAVVYNRKG MFETLPGRITIGERLDEHSI ---------------KYCVE EAARYARDSS---------D FIFFRHGRIYPNEVVAFENV
 fna01717  DVGRTKAEYWAMAVVYNRKG MFETLPGRITIGERLDEHSI ---------------KYCVE EAARYARDSS---------D FIFFRHGRIYPNEVVAFENV
JAHPYW101  DVGRAKSENWVMMIVYDKSG RYRIGQTELTVGESVDRDSL ---------------VKCIN EAQALVPSAK---------S LLYLRDGSVYDKERNDFESV
Meg22Bin1  DVGRAKSENWVMMIVYDKSG RYRIGQTELTVGESVDQDSL ---------------TKCIK EAQSIVPDAK---------S LLYLRDGSVYDQERET---V
D4993C5H2  DVGRAKSENWVMMIVYDKSG RYRIGQTELTVGESVDQDSL ---------------TKCIK EAQSIVPDAK---------S LLYLRDGSVYDQERET---V
 RLG97969  DVGRTLSEYWAMGIVYDRDG KFEICPGKITLGEDLDEASI ---------------DHCVK EAHRYAPEGK---------S LIFLRHGNV...........
MCD623836  DVGRRKSEWWAMSVVYSRDG RYETLPGEMLVGEDLDAEAI ---------------RKCVS GAIAGEEKPK---------S FVLLRDGDISEKELRAFEDE
MGH577974  DVGRQKAEWWAMGIVYDQKG LYEILPGEMIVGEDLNIEAL ---------------KCCTS RALRASA-PN---------S FLILRDGDVSEGESETFKTL
MDO872460  DVGREKSTYWAMGIVQDKIG NLEAVEGNLMTGEDLSLQSF ---------------KNCVS KAVAQGV-PD---------S LILLRDGDVQETEIDEFKKV
MCL573225  DVGRSLSEYWSFGCVLSKDG LFSIWHGKQMKGEDLDKNSI ---------------EESIE FAERNIPQSN---------T LIYVRDGSIAESEFENFKEI
 bin01460  SYEEGITRRE--GVVALEIA NNWGDPIGRFFATGIEVKKK EGAIVVDIESISTLMKKALD RVEKEIADPEKSKEKDY--V VVHIKDRYADSVYDSIAKTI
MCD644642  TYEQESSVTERKGVVALEIA NAWGDPLGRFFGRETVIERE KGAIIVDLESIDQLVKNSLD MIENELVDVEKSKEKDF--I IFHVKDRYAESVYDKIGEVI
MCD624170  VPT--SIGYERRAIVAVQLF NEWGEYEHSVTAKLSNVWKD EENRPCFETDKVRDLVLNIA ---DEIPIEDKEISDNY--L ICHITELYSDKVLNTIMKTL
MGB995986  KPG-IFGGKRRAGIAALQMY NNWGDYENSVYGKLMFIYEE NKGMLDLGDNESRDKFTQLV T------TIAREVKNRN--V VVHLTDLYAKSFHGLLHEVL
   HrAgo1  DVWHGRPGIDKSIAGIVFST DKGLHYTANYTITPRKGLEF IHNLGKIIITQLQNHYNATR QYFENILIFRDGVGNTQ--Y NKILQEEFKSIQQELTNSSI

 fna01589  AEGIQRLDLLAFGTRS---- ---------FKLVRW----- -GQQPPIRGTMVRLPDRSIL LYTYGYIPYLNV-YPGPRVP SPLEILEHHGTTSIETICKE
MFC180375  ANEVSKLDLVAFGNRN---- ---------LKMVRW----- -GKEPPIRGSMVRLPDNSVL LYTFGYIPYLDV-YPGPRVP SPLEILEHHGSTSIDNVCKE
MDR271946  AKEIAKLDLIAFGTRD---- ---------IKLLRW----- -GQQPPIRGTMVRLPDNSVL LYTFGYIPYLDV-YPGPRVP APLEIIEHHGSTSVDIICKE
 TBR08567  SKDVHRVDLVAFGERD---- ---------IKLMRW----- -GTNPPIRGTMVRLPDNSVL LYTSGYIPYLDV-YPGPRVP SPLEILEHHGQTSIETICKE
MFY930001  EKDVHRVDLVAFGSRE---- ---------IKLMRW----- -GTNPPIRGTMVRLPDDSIL LYTSGFIPYLDV-YPGPRVP SPLEILEHHGQTPIETICNE
MCL587675  LSEGITLDLVAFGTRN---- ---------LKLVRW----- -GVQPPIRGTMIRLPDESVL LYTNGFIPYLGM-YPGPRVP SPLEILQHLGNSPIDDICEE
HET657446  LGAIHSHDFLAVERRG---- ---------VRILRL----- -GHEPPIRGTMVELGRRNYL VYTQGYIPFLRC-YPGPRIP NPLEVVEHHGDSAADVVCSE
WP4134311  LGNIHSHDFLALERRG---- ---------LRFFRL----- -GMEPPVRGTVVELGPRNYL LYTQGYVPFLRQ-YPGMRIP NPLEILEHHGDSSAERVCRE
 TXG99657  LGDIHSYDFLALERRG---- ---------IRFLRL----- -GKEPPVRGTVLELGGRDYL VYTQGYVPFMRQ-YPGMRIP NPLEVVEHFGDSSAERVCSE
MCG312924  LGDVQSFDFLTLERRG---- ---------IRFLRL----- -GHEPPLRGTVIQLARRNYL LYTRGYVPYLRA-YPGMRVP NPIEIAEHHGDSSADKVCSE
MCE965210  LKEIKMHDLVAIGERD---- ---------IRFFRY----- -GQFPPLRGTVIQLGKTNYL LYTRGFTPYLRT-YPGAHVP LPLDILEHHGDSPRDVILTE
 CUR52642  LKEIKMHDLVAIGERD---- ---------IRFFRY----- -GQFPPLRGTVIQLGKTNYL LYTRGFTPYLRT-YPGAHVP LPLDILEHYGDSSPDVVLTE
 HWY27817  LKDVPKHDFVAFGDKR---- ---------IQLFRF----- -GKYPPLRGTAVKIGHGNYL LYTRGYVPYLQT-YPGARAP WPLD-IIHEGDSSADTVLSE
MEM586797  AKDVKIKDYVTIFKRG---- -------RGIRFMRKK---- -GKYPPLRGTVIKIGERNYI VFTQGWIPYFRT-YPGLRVP VPLEIVEHYGDSPMETICRE
MEM011721  AAKTSRIDFLAFGERG---- ---------IRFLRK----- -GSYPPLRGTAVELSSGNYI LYTRGYVPYYGT-YLGQRVP QPIEITEHYGDSDADTICRE
MFN879258  SEIVPRRDYVALGWRN---- ---------IQFYRT----- -GMYPPLRGTYVKFSDSNLL LYTSGYVPFLKT-YPSIRVP QPVEILEHHGDSPWNIILEE
HEY295644  CALVPQTDFIALGWRG---- ---------IQFYRP----- -GLYPPLRGTYVRFSETDLL LYTVGYVPYLRT-YPGARVP QPLGIVEHHGDSPWDLVLKE
 TKJ43753  CKDISEKDFVTIGKRG---- ---------IQFYRP----- -GIYPPLRGTYVKFSDKNFL LYTDGYVPFLRT-YPGPRVP QPLEILEHHGDSPWNTVLEE
 fna01259  LESVPRHDFIAILQKT---- -------RRIKFFRK----- -GKEPVLRGTVIDLTNNNWL LYTKAYIPFMRV-YPGPRVP RPLEIVQHEGDTPTDGVLSE
MBI423204  LKEVPKSDFVAITTDQ---- -------KQLRFYRS----- -GYHPIIRGTLITLPDQMRL LYTKAYIPFLGV-YPGARIP IPLEVYHDAGDSPTDTVCSE
 fna00701  LDKLVKTELISADHQ-CTFV EIRATSKVPFRLFEVTPS-- -PGIQKEWVDNPTIGTYDCL SENEAFICNTGPPFGHKGTT KPLHIIK-DGPMSIESVLED
MEM211205  LGIVAEKGLIAKDHQ-CTFL EIRTTSRIPFRLFYVTTL-- -PGTQKEMIYNPTIGTYVLL SENDSFVCNTGLPYEHQGTT KPLHVVKVEGPHALENLLED
MGA238515  ITRLKGSNVIPQESM-CTSI EIKTTSRVPFRMFKVEPM-- -TGEQKENVENPMIGTYFML SDSEAFLATTGTPYSHFGTA KPLHVIKIEGTMPLTKLLED
MFZ316824  IETLSNEGTINQNAS-GTFV EIRKTSGIPLRLFEIQLP-- -SGMQAEKVYNPIVGTYRIF SENEFFICNTGYPFRHEGTT KPLHVIRLEGHFPIKDIIED
 NVM53154  FDILAKEGKVNKDAQ-CTFV EIRKTSRTPFRMFKIVMP-- -IGLQDEKIYNPTIGTYIPI TEDEFFICNTGFPFKHKGTS KPLHIVKAEGNMSSHKLTED
MGI008690  LQKLTSEGMVSKDYS-CNFL AIQKSSSTPLRLFKVDNR-- -HGAQFQSIDNPTIGS-HMV SFDQAFLCTAGFPYRYNGTS IPLQITKIDGTMDFKYLLED
 RLI97245  IDALKQEGVLAAEAS-CTFV EVQKTSMIPFRIFEVSTQ-- -PETHGERIEAPFIGTYVQV SENEGFLTTTGQPYKLIGTA KPIHITKREGPIPLEQLMED
 HUV01944  ISRLKKEGLLPEVIN-CYGV ELRKL--NPFRIYE------ -GDNETAQMCGIGSVTLLSN SDAILATTGAPSLTQGTANP LLTKVVSFEGTFDIKEVCRD
KAF543266  ISKLKEEKLIPQRIN-CYGI EFRKV--NPFRIFD------ -GDRETAKMCSIGSVVLLSG NDAILATTGAPSLKQATANP LFVTVISFEGSFDIKEVCRD
MGA329121  KSELLSNGSMTSDGK-ITCV NVKKT--VPYRLYD------ -SQGKQQSECKIGSYLILDD HNGIIANSGAPLLRQGMARP ILVETVPPFDCIDIKSALED
MBT015896  KSELLSNGSVTNDIK-ITCV NIKKS--VPYRLYD------ -SQGKQQSVCKIGSYLILDE HNGIIANSGAPLLRQGMARP ILVETVPPFDNTDIKTALED
 bin00806  VEEYLSPEITKSLG--LAVI EIRKN--VPFRIYD------ -IVDQWVKNPLIGRYALIDE NTAVLCTTGYPLITQGVAEP LLIYAHNMYREFKIREILQD
 fna01190  TGESPFKN--------CAIV AIKTGE-VPYRIFR------ -EVNGKVIKPLSGDYYPLGQ STVVVCTVGVDEYKHGMPKP ITIEVIPTKGEVEAVKVARD
 fna01717  TGESPFKN--------CAIV AIKTGE-VPYRIFR------ -EVNGKVIKPLSGDYYPLGQ STVVVCTVGVDEYKHGMPKP ITIEVIPTKGEVEAVKVARD
JAHPYW101  VKESGLQD--------SAIL AIREI--TPFRVYR------ -GSLSDIWRPHSGDYYVLDE SNTVLCAAGADEYQHGTPSP ITIDFISVAGSIDRFKAIED
Meg22Bin1  VKK--FQH--------SAIV AIRET--TPFRVYR------ -GSQSDIWRPHSGDYYILDQ SNMVLCAAGADEYDHGTPSP ITIDYIPIVGSIDRLKAIED
D4993C5H2  VKK--FQH--------SAIV AIRET--TPFRVYR------ -GSQSDIWRPHSGDYYILDQ SNMVLCAAGADEYDHGTPSP ITIDYIPIVGSIDRLKAIED
 RLG97969  .................... .................... .................... .................... ....................
MCD623836  ISKTDIQN--------CGII SIKKD--VPHRIFR------ -RQGDKIFKPFSGDFVVLDE NLVMVCCAGIDEYEHGTPRV RVIEGESIEGDIDIVHVAKD
MGH577974  ISEAKVQN--------SAIV SVKKN--IPHRLFR------ -SVNGEIYKPISGDFIELDK NLIVICLAGVDRYQHGTPIP KVLEVIPVSGHISAQDVARD
MDO872460  VDSTGIKN--------CGIA WIKEM--IPHRIF------- -GGKTKVEKPDSGDYIPLDE NNIMICCAGLDEYGHGMPKP RVVELTLIKGELDIIKISQD
MCL573225  VEQYPNLEN-------VAIV SYMKN--VPFRIFR------ -SYNGRISKAFSGDYISMDP SHYIICNSGRDFSRQGTPST RSLEIINLRGKIDKKSIVED
 bin01460  ISKG-FKK--------FKII HVQKE--GSLRLYD-----P SKRITRAWPKEGSYWYLERG KIAFLFTLGRWQYSISPRSE PYVIGARNVSPLQTNFVKGN
MCD644642  TSEG-FKN--------FKII HIQKR--GSLRLFN-----P QEKIHRAWPREGSYWYLQKG KIAFLYTLGRWQYSLR--SE PYTIGARDVSPLQVNLARSD
MCD624170  NSRG-FEK--------VKLI RVQRE--GPLRIFD----TS SEKPAYAWPIWGTYWFLESG KIAQLYSSGAWRYSPLPTSK PYTIHYFTSSPVQVVLEKPP
MGB995986  INNG-ANN--------VKII RAQRS--SPLRLYI-----N IEEASQAWPEIGRYWFLESD KIAQLYTTGKWQYFTH--KP PYAIPRYTIRPIQVSLEH-P
   HrAgo1  FSEKHPKI-------AIILV NKRINR-RLFHKNK------ QGQILNPKPGTFIEDQYIKS EFSNYYLVPHFSRFGTTR-P IHISVIYNNTKYVNFQFVEI

 fna01589  ILSLTKLNWNFAKFCIKAPI TISFAKRVGNILRNAPPDIE PQN----KFKFYM
MFC180375  ILALTKLNWNNAKFCIKAPI TIGFARRVGNILRNIPPDAE IQN----KFKYYM
MDR271946  ILALTKLNWNSAKFCIKAPI TVSFARRVGFFLRNAPPTLT PKG----KFKFYM
 TBR08567  ILALTKLNWNNAKFCTKAPI TIGFARRVGSILREMPPDID IKDEVGTKFKFYM
MFY930001  ILSLTKLNWNNAKFCTKAPI TIGFAKRVGAIMREAPPDTE INNGA--KFKFYM
MCL587675  TLALTKLNWNNAKFCTKAPI TIGFAKRVGEILRNAPPDSD KYLQN--RLKYYI
HET657446  ILALTKLNWNTCAFAGGSPV TIAFARQIGSIMKELPAGIK PLT----RYKYYM
WP4134311  ILALTKLNWNSCKFSSADPI TTLFSQQVSTIIKEMPDGVQ PAK----KYRFYM
 TXG99657  ILALTKLNWNTCKFSSADPI TTSFSRQVATILKELPEDVV PAT----KYRFYM
MCG312924  ILALTKLNWNNCGFASRDPI TIAFSRQVGRILTELPAVGI EAPLT--KYKYYM
MCE965210  ILALSKMNWNSAEFSLAKPI TLLFSKRVGEIMASLPDSMN PRH----EYLYYM
 CUR52642  ILALSKMNWNSADFSLAKPI TLLFSKKVGEVMASLPESVN PRH----EYLYYM
 HWY27817  VLSLSKMNWNSAQFSLAEPI TLLFSDRVGEVMSHIPRGKE PRH----EYLYYM
MEM586797  ILALTKLSWNSADFCISEPV TLAYSRQVGRILAYVPEEVK PRP----QYLFYM
MEM011721  VMALTKMNWNNSDFSIREPI TLQFAREVGLILAHLREGVE PRP----QYLYYM
MFN879258  VLALTKMNWNTSDFSNAKPI TLAFSQKVGRILAELPPNVQ PRP----EYRFYM
HEY295644  ILALTKMNWNTADFACSEPI TLAFSRRVGQILAELPPHVT PRP----EYRFYM
 TKJ43753  ILALTKINWNTADFSCSKPI TLAFAQRVGDILAELPPGMK ERN----EYRFYM
 fna01259  ILSLSKLNWNSADFTSFLPI TLQFSRQVGRILRELPPGIT PET----KYLYYM
MBI423204  ILGLTKLNWNNADFTGRLPI TLEFSKRVGKILRETLPGTQ PQT----RYRFYM
 fna00701  EFYLSNLTWTKIDDCSRQPL SIKMTD---IRLREFAGDYD RDA----LRF...
MEM211205  VFYLSNLTWTKIDDCSRQPL SIKMTD---IRLREFAGEYD VDA----LRF...
MGA238515  VFYLANLTWTKIDDCSRLPL TIKMND---IRLREFAGEYD QDA----LRF...
MFZ316824  VFYLSNLTWTKIDDCSRDPI SIKMTD---IRLREIAGEYD EDL----LKF...
 NVM53154  IFYLSNLTWTKLDDCSRYPL TIKLTD---IRLREYAGEYD KDA----LEF...
MGI008690  VFWLSNLTWTKIDYCSKLPI SIKMVD---IRLREIAGQYS EDA----LDF...
 RLI97245  IFMLTNLTWTKIDYCARDPL TVKMND---IWLREIAGDYD RNA----LKF...
 HUV01944  VLYLSELNWGSPLRGMKMPV TIKIAE---DVIPFAERGFS LDY----IPW...
KAF543266  ALYLSELNWGSPLRGTKLPV TIKIAE---DIIPFAERGFR PDY----IPW...
MGA329121  VYYLSFMHWGSIMVKMKLPA TLRYAD---ALTPFALRNIR ITG----VPL...
MBT015896  VYYLSFMHWGSIMVKMKLPA TLRYAD---ALTPFALRSIR ITG----VPL...
 bin00806  ILCLSELNWIAGDKPSKLPV TISLAE---ARALFAEKGIT PAS----RLP...
 fna01190  AFYLTYLNWGSPRQSYSMPA PLRLAH---TLASELSAGIR TGG----RPF...
 fna01717  AFYLTYLNWGSPRQSYSMPA PLRLAH---TLASELSAGIR TGG----RPF...
JAHPYW101  VFKLSYLNWASPGKSYSTPA PLRMAH---RIARELSLGIE RKT----VP....
Meg22Bin1  IFKLSYLNWASPGRSYS-TA PLRMAH---RIARELSE--- RGT----VPF...
D4993C5H2  IFKLSYLNWASPGRSYS-TA PLRMAH---RIARELSE--- RGT----VPF...
 RLG97969  .................... .................... .............
MCD623836  IFYLSYLNWGSPGRSYSEPA PLRLAH---NLASELSRGLT ISG----PPF...
MGH577974  VFYLSYLNWGSPSHGYSSPA PLRLAH---ILASALSQGLT PFG----PPF...
MDO872460  IFYQSYLNWGSPSHSYSSPA PLRLAH---KLAYQLSKGLR RAG----PPF...
MCL573225  LYKMCFLNWGSPRSPYSDPA PLHVID---DYLYELSRGIQ RHG----LPF...
 bin01460  EGSNLTVDDLRH........ .................... .............
MCD644642  GDSILTREDLRP........ .................... .............
MCD624170  NEQL-TRNDLKNI....... .................... .............
MGB995986  EETL-TKDDIRDI....... .................... .............
   HrAgo1  ANILCHLNYNWA-GTVRIPA SVEYAHKVADFIGSNQITSI APE--LLQTQFYL
```

**Alb/Tir domain Alignment (PAUP file) used for phylogenetic analysis**

```
[Name: fna01588          Len:   512  Check: E5684DA7]
[Name: MFC1803758        Len:   512  Check: 3DD47C25]
[Name: MFY9300139        Len:   512  Check: CF4F3B48]
[Name: TBR085681         Len:   512  Check:  8043026]
[Name: MDR2719461        Len:   512  Check: 849BD9BB]
[Name: MCL5876755        Len:   512  Check: 6A536AC4]
[Name: MCW4019376        Len:   512  Check: 7D919B5A]
[Name: MGB7123385        Len:   512  Check: 8D9FB549]
[Name: WP179369097       Len:   512  Check: 73EE5F44]
[Name: VVB64402          Len:   512  Check: A0A81A4D]
[Name: MCF2138277        Len:   512  Check: 146562DF]
[Name: fna01260          Len:   512  Check: 8D941FAE]
[Name: WNZ28653          Len:   512  Check: A6BD0EA0]
[Name: MFX0209723        Len:   512  Check: 998B7C1E]
[Name: MDG6904615        Len:   512  Check: 104BB46A]
[Name: HWY27816          Len:   512  Check: 320328E8]
[Name: TMI14703          Len:   512  Check: AA371145]
[Name: CUR52643          Len:   512  Check: 1FD42BED]
[Name: MCE9652099        Len:   512  Check: 5A60E0D2]
[Name: MFQ5910271        Len:   512  Check: BC6CA70A]
[Name: HYD51135          Len:   512  Check:  401AEEF]


begin data;
 dimensions ntax=21 nchar=512;
 format datatype=protein interleave missing=. gap=-;
  matrix
 fna01588  ....MELNSEFAEELIHKGF ESPDLDYKLKFDGSTHA-WM ELAKDIFGMANYGGGYIVFG VE-----------DGTFRPT GMNIDFHKDVQEWIDRVSKW
MFC180375  ....MVLNPDIAEELLSRGF ESPDLDYKLEFKNDTRS-WM EIAKDIFGMSNFGGGYIVFG VK-----------DGTFEQV GLEKTFHIDSQVWVDKVQKW
MFY930013  ....MELTPELAEELIHRGY ESPDLDYKEKFDASTGA-WM EVAKDVYGMANHGGGYIVFG VQ-----------DATFKPV GMDQSFHIDSQTWADKFSKW
TBR085681  ....MELTSELAEELVHRGY ESPDLDYKEKFDNSTGS-WM EVAKDVYGMANHGGGYIVFG VQ-----------DATFKPI GMDQSFHIDSQTWADKFSKW
MDR271946  ....MELNAEISEELIHRGF ESADLDYKLEFDDSERA-WM EIAKDVFGMANYGGGYIVIG IE-----------DGTFKPI GLDPSFHKDTQEWINKISRW
MCL587675  ....MEISPELAQELINRKI ESSDLDYKEKFNDTTKD-WL EIAKDVYGMANSGGGYIIFG VQ-----------DGTFKPI GIDENFHKDSQTWVEKFSKW
MCW401937  .................... .................... .................... .................... ....................
MGB712338  ..............LFSRNY ESSELDFKESLETAKGSNFA KVARHFFGMSNYGGGFLLVG FQ--------AKPTGGFLPV GLPEDFHIDQAELQGKFNAL
WP1793690  .................... ETPFLDFKTTMDVGKDSDFP KIVKDVFAFSNNGGGYLIIG VK--PNDHSNKDIRGSFIKN GLPEDYEADQATIQQKINAY
 VVB64402  .................... ECPYLDFKFTTHIERISDFP EVAKDIFAFSNYGGGWILIG WK--------EIKSSQYVPE GLPADYNVDQAKLQEKFNSY
MCF213827  ..............LMGQRG EMPWLDFKEILHIERGSDFP EITKDIFAMANYGGGYIVFG VR--------EKDTGSFEFI GLPSDFHVDGATVTEKFNSY
 fna01260  MTITEEVQKEYDVFLSYQSL DEDFAKKLGEKIQSVTYRGR RLKVFLAPWDVQPGDNIVVK LD--------KGLANARYFL LLLSPEALKADWPTAERAAA
 WNZ28653  ........ESYDLFVSYNKA DVEFAKRLVSAVENQQYNGR KLICFFAPWDIEPGENILLK IE--------KGLTTSRFVC LIMSPDWLKSDWTTLERVVP
MFX020972  .........EADIFISYNNA DSDFSEKLVSRIEQESYQNR NLICFFAEWDIEPGENILLK IE--------KAIEKSRFIG IIMSPEWLKSDWTTLERVVP
MDG690461  .......EKEYDLFISYSRK DLQFVERLVKRIESVQLEGR ELRCFFAEWDILPGENIVVK LE--------QGLKRSKFIG LVMSPDWERSDWATLERVIP
 HWY27816  .........EWDLFLCHTAV DKEWVRELAARLENETSQQR KIRVFFDEWEIQPGTNVVVN LE--------NGVAKAKFVG VVLSPEMLDAEWPTLEWTMA
 TMI14703  ...........DLFLSYTRV DKDWVRDLAAQLERESLRGR QLNVFFDEWEIRPGANIVAS LE--------NGVSKARFVG VVLSPEMLAGEWPTLEWTMA
 CUR52643  .........EYDIFLCHNSL DKPWVQKLAESIENEDYNNR KLKVFYDDWDIIPGENNVLK IE--------YAMKKSRFVG VTLSENSINAEWPNMEWTMA
MCE965209  .........EYDIFLCHNSN DKPWVEKLAESIENEDYNNN KLRVFYDDWDVIPGENLVLK IE--------HAMKKSRFVA VILSEHSINAEWPNMEWTMA
MFQ591027  ..........YDLFLCHSGV DREWVKNLAIQVEREEVNGR RLRVFLDEWDIRPGENVILR IN--------EGLTNSRFVG VVLSPEMVQAEWPTLEWTTA
 HYD51135  ...........DVFLCHNGA DKDWTRALGERIEQENWGGR KLTVFFDEWDIEPGANILLK LD--------EGLRTSRFVA LVLSPEMLRSDWCSLEWTSI

 fna01588  ATGKINISYIEHIGEIEGER RKFPILCVHASIGSFVIPKV DGKYKLKSGEEKIAFKKGIL YIRRSTSTVAASGNEY--WE SFWSLLRRTAERVGSVGTPL
MFC180375  ATGNINLSYIEYLKERNGIE RKFPILYVHGSIGNFVVPKV DGKYKLPSGEERIAFRKGLV YTRRNTSTEHASGDEY--WE LFWSLNSRTAQQSGSRSVPL
MFY930013  IDGKINLTYLEHMSNVDGKT MKFPILYVNGSVSSLAIPKT DGIYNLPSGQSVTAFNRGVI YTRENTSTISATGNEF--WK LFWSLTKRTSEQTGSQGIPL
TBR085681  IDGKINLTYLEYAGKVDGNA VKFPILYIHGSVSSLAIPKT DGIYNLPSGQPTIAFNRGII YTRENTSTVSATGGEF--WK LFVSLTKRTAQTTGSEGTPL
MDR271946  ITGKLSIDYMEPILNVEGQQ KKFAILHVHGSIGTLVIPKD DGNYTLPSGVKKCAFRQGII YTRRNASTAQVTGDEF--WQ LFWALTHRTTAVTGSNNVPL
MCL587675  TTGHIKITYFEHISLVAGKN RKFPILFIHGSIGSLVIPKS EGAYKDEHGIDKVAFRQGTV YTRRNSATVAATGEEF--WH LFWALLKRTSEERDTSEIPI
MCW401937  .................... .................... .................... .................... ..........QKQGKVNADA
MGB712338  ASVPLALGYRELERTCDGIT RRFAVVYI-PPAPEPITPVS DGRFRDAKGKDRTAFVRGDV LIRRGTSTMRATPSE----- --------IVW--ITQRAKD
WP1793690  TDNPIEIDYTQFERMVNGEQ RKFAIMFI-PASTSLLKPTR DGIYKING-VEKKAFLKDTP YVRRGTQSIPAEPPE----- --------VAR--IQKRIED
 VVB64402  SNIPIELSYIEFTKYSK--- -RLAAIYI-PPAYDILKPIK EGIYKKGD-KDRIVFKKGDI FYRRGTQSIPPSDRE----- --------VEI--ITKRLKK
MCF213827  SSQPISLLWVEHSEIIRDTP LKFAIMYI-PPSKTVLVPIK EGAYGRGH-QRKIVFKANDV FIRRGSQSIKASSNE----- --------IEW--IAARAKD
 fna01260  IYSDPSGRLGKVIPAIRRPC K--------LPPLLAFRNYL DFRQDSNFNAEFFRLIS--- --KLTGQPLPSEPST----- --------LSW-HQGIRQES
 WNZ28653  VHSDPAGIKGRIIPILRRDC S--------IPPTIRILNWL DFRTDRNFNREIKRLIS--- --RIRGIS-PRE-------- ----------IYQQNIETDF
MFX020972  VYDDPAGLKGKIIPILRRDC E--------IPPSIRILKWL DFRTDSNFEREVKKLIS--- --RLKGEP-YRS-------- ------------LLKREEQI
MDG690461  IYEDPAGYKARVLPILRRGC V--------IPPSIMILKWF NFQNDTGFEREAERLIS--- --RIIGRS-PRD-------- -----------LAQATVTGR
 HWY27816  VYEDPSGRRGKVVPILRRDC D--------IPPSLRIRNIL DFRKPEKVERSYAKLLA--- --ILSDTPLPRG-------- --------AGEPSSTSSKPT
 TMI14703  VYEDPSGRKGKVIPILKRDC E--------IPPSLRVRNVL DFRTPERQERSYAKLLA--- --VLRNEPLPRG-------- ----------HTFQGVAPMQ
 CUR52643  VYNDPSGRRGFVIPIWLGNC E--------IPSSLKIRTVL YCRNNIEYNKSYTKLLA--- --LLRNQKLPRG-------- -----------QMRPSTLNS
MCE965209  VYNDPSGKRGFVIPMWLGGC E--------IPPSLLIRYVL YCNTDIAYKKSYTKLLA--- --LLKNQRLPRG-------- -----------QMRPSVLNS
MFQ591027  LYQDPAGKRGRLLPILLRDC D--------VPPVLSVLNRL DFRRDASWRPELARLLA--- --VLREAPLPRE-------- -------IERLSDELVGSGT
 HYD51135  LSADPVNRLGRLVPLLRRDI DKASGQRIDIPPILRPFNFI DCRDDKTFSRGYARLLA--- --KVRGEAPPRGPSC----- -----GSARGAVGSAAHPPP

 fna01588  EVISALSRKAEPDNVEETLW FNLFPVVEIPD-LIHAADTE FRYPREIFETIENRGDQSQQ YNKI-PPFLLEDKKIYSFSP FDENNPLSQ--CTDLAHRTL
MFC180375  EIISALNIKAQPDNIEETIW FNMFPVLELPD-HIYAAEAS LRQPLEVYDQIENILGSRAN YDV--PAFLLEDKKLYCFSP FEEGNPLTV--CMDMIPRKL
MFY930013  EVISVLNKKAEPDRVEETLW FNLFPVTEMPDSIHVADTDF RTPI-EIYDHVRKILSDSGQ SFVSIPSFRLADKKIYSFST FDNDNPLTL-----------
TBR085681  EVISVLNRKAQPDVIEETLW FNLFPITELPDFIHVASTES RSPK-DVYDHIREEFAKQGI TKYDIPSFLLSDKKIYSFSP FDDMNPLTF-----------
MDR271946  EILSTLNKKTKPDDLEETLW SNLFPVIELPD-MIYTANTK YRTATEIYDKIRNEHISKEQ KILM-PPFFLEDKRIYSFTN FDEINPLSL-----------
MCL587675  DALNVLNHKMKPDCLEETLW SNLYPVKELPD-KIFSANTK YRFANDIYAKIKENLKGEVK T----PAFLLEDELIYSFCP FDELNPLSL-----------
MCW401937  FKVNALPEPKTAKPIEETLL SNLFPVVSIPT-FIYSSSTH ARTTRFVFDKLGDAVKSHP- -------FLPKNRRLYSF-- DNFKDSSCV----------F
MGB712338  TAYQISLLSGEPDRIDEVLS SNIFPAIRLPA-RVFRCHVD LRGKPVPDHGLQSCLVDGRT IHS----F--EDPSRTSL-- QPFIEPGSL-----------
WP1793690  ENYRLSLLSGEPDKIEERLF SNIFEVKSIPK-RIHIGTAK FSNYFEDIQHLQEKYPGVRH FPLR---YRHYEDKIVTFQD LSDSNDLH-----------H
 VVB64402  ENYRLSVLSGEPDEIVETIY SNLFPVIELPE-FIYSGLKK DYDNKSIQVLLKQEGVNPSY MFK----FIENEGRIYTFDN LYEEMNPY-----------R
MCF213827  ERHNLSILSGEADEVRETLF GNLLEVKKLPD-HVYIGEVL PRRTKGSQRFPGP------- -------YVIFDHKCYSFLN LSRPSFKS------------
 fna01260  SPLQSLIDSSEPDDVYEKIF SNLFPVRNLPA-RVYSAPTK FVDRKQVQVYFPRD-----S IV---PPFILKEHRLFTFAN LSAETHPF-----------K
 WNZ28653  TP-QPV-DSLKPDFQKEILS SNLFPIIELPR-IIYRAKAK VRKRSDVWEMLGEG-----V SI---PVFALREESQEIFSF AKLTNPQQ-----------K
MFX020972  PTS---FDSTSPHVQDELLA SNLFQVVELPN-FFYRALSR VTKRDDVWKMLGED-----V LV---PVFALRDETKEIFSF SALNNPQQ-----------K
MDG690461  PLSRQVVDSSSPDFQNETLA SNLFPVVRMPS-RMFIGHSK VHKRNDVWAELGSG-----A ITPP-FAFNEDTQRIYAFSP LSEEGNNL-----------R
 HWY27816  GMEHVVEPLEFADRVTEQLA SNLFPVRAYPS-TIWSADTI AQSIGEVYAHLRKFLM---S ETL--PTFCLRENKLHTFWN LNDKACPF-----------R
 TMI14703  SPQIFAKPLEFADEVQERIA SNIFPIESLPR-WIWSAETE ARSYGEVYRHLRKIIV---A DTL--PTFILRQNRIYTFCN LEDRGSPF-----------G
 CUR52643  LPTEMLFPIEYADEVEEQLA SNLLPVLKIPR-HIWSGPVA SLTYREVFDHLNRKVK---G AH---PTFMIKESRIYSFCD LNDKYCIF-----------R
MCE965209  LPTETLFPIEYADEIEEQLA SNLLPILKIPN-HIWSGPVG SITYRDVFDHLHRKVK---G VH---PTFMMKENRIYSFHD LNDKHNVF-----------R
MFQ591027  RELKTGERSWEPDFQEEEIV TNLFGIASLPE-YVFIGNTW ARQPGDIFE---VTR----G KPI--PPFVLREGRIYVFED LTRRRSTL-----------R
 HYD51135  VLATFAAERQEPDSVQETLI SNLLPVCDMPK-VVWSAATS LKSKTDLPRG---------S AL---PPFILRENHVYTFAD LSRPDNAF-----------K

 fna01588  FDKEEHATITSIPTKDWLND ETKHQKLVKLLNFNLKDLCH KKGFHYDNRKDRYYIRYF-- GGRIPQITWKPYQKTSTRPL VYLRFNEK-TGRLMYCEHFA
MFC180375  MDL-GGKVYDKISIKEWVKE GPNRQKLVKLLNYNLKDLCW KKRFHHDPRKNRFFIRYY-- GGMAPEITWKPYRRTSTRKL VYPRLNKE-TGHLYYCEHFA
MFY930013  ---CIRTKNPSISTKDWLED PKQHQNLVMLLNYNLKDLCK RKHFVYDRKKDRYYIRYN-- GAAVPEITWKPYKSTSTRKL VNIKLNKE--GKLFYCEHFG
TBR085681  ---CTDTQFPAIPTQEWLVD ETKHQNLIKLLNYNLKDLCR KKRFYHDVKRDRYFIRYF-- DGPVPEITWKPYKSTSTRKL VFIRLSKE--GNLLYCEHFG
MDR271946  ---CITSKPEAIQTKQWLDD NKKHQKLVKLLNFNLKDLCK RKGFFHDQRHDRFFVKYF-V GQSVPEVTWKPYAAKSTRQL VYPKLSKT-DRSLLYCEHFA
MCL587675  ---CVSSSGAEHSTQEWLDD PKLNQKLVKLLNFNLKELCK SKRFFYDADRDRYYMRYF-- GGSVPSVTWTPYKATSTREL VNLKINKN-TGRLTYCEHFG
MCW401937  TPLIDKNAITEEYSSTWVRD DDKRNDLVYLLNLALKKYCE KRNMYYDRRHDRFVCLLD-- SGRDSTFTWRANTRHIPRKI AKKVFDKQ--GNLVFCLHYA
MGB712338  ----KSD-----PVSDWQND RDLARKLMQLIDSALVRKGA HLGMEFDWARRRFFYPLP-P NDSKREEEWEGITKRSSRQV AVRRYLGS-LKREVVIHSSV
WP1793690  ELVLRDD-ISHESTGSWLDD GPKENIVTSLLNKEIGEQAR RQGMRYFDKTKKIYYPTT-- -ADSRIESWPTRYKGVSNKT VAKKMWAEQLHRSVFVHLAA
 VVB64402  KLVHVKS-LSREPVNSWIDN LSKNKLLVQLLNLEFRHYAV SLGLSYFPLSDELYYSTE-- -GETRRITWRGRY-GKSTRT VATMMFADQLNRYIFRHAAI
MCF213827  -IVKQDTILRI--RTGDLDE EDLQ-IIVLLLNNEIKTACY SIGLKYQSRGRKYYFPLRPR DGSRRRIKWSGRYRASTRTV AQKIIPEV-GGNLEYCSHAA
 fna01260  GVVEDY-DIRSELTSEWIQD ENRARWLVELLNDCIRVHRF SLGLYLDRRGKKLYYPKGVL ASEKI--NWKAHQRTATRHL IKERKNPQ-GQTTFFLHRAV
 WNZ28653  LIKLVNNSETNEVLTAELIN SDNSSVLIELLNRSMTEHMK NIGMVYDWKNKKTFYPLEKN GDEMRYSKWTVKNREYPRLV VQKSK---SG---RYFIHKS
MFX020972  LIQVVQ-NNTEKIPIFNLLK TERYPILIELVNRAMTAHMK NIGMVYDWKNKKTFFPLDDF NRDIRYATWKIGGREYTRFI VKKSN---SG---KYYIHRS
MDG690461  KIIIES--TVEEYRTTDLL- ADSASIVIEILNRSMTAHMR NLGMNYDWNNKKTYFPLGHE GS-IRYGRWGVGNREFRRTL VRSSG-GLPG---YYAHRSC
 HWY27816  GLLSSEVAISQEESKQWVLD QNKRNWLLELVNRTLWNNCE NIGLYRDRKHNRIFFLPQDG HDRVV--EWNTGRRKSSRTV VKRYQRGNQGN--VFWAHQS
 TMI14703  GLVSPIATIRKEDTYLWSAE GPDQDGLLELLNRALVNNSE NLGLYHDRQHNRTYFLTNNG GNRIV--EWDTGTRRSRRTV VKRYQKGPNGE--VFWAHLS
 CUR52643  ELLTAE-SIEKNEITPWINN LDKKNGLIEMLNRTLRNHCY DLGMRYDKKHERFFFLPYGG GNREV--SWNTGTRKSTRTI VKKYQKGKDGS--IFWAHQS
MCE965209  ELLTAD-SIEKTELTPWINN LDKKNGLIEMLNRALRNHCH NLEMRYDKKHDRFFFLPRGG GNREI--DWNTGTRKSTRKV VKKYQKGTDGS--IFWAHQA
MFQ591027  KAAAVA-DVQTYSTKHLIDS ESTQDYVVELLNRTFWKHCR SLGLAFDKVSGKHFSYAENG EPRTF--GWK-PNKRWSNRE IARPKLYGGRV--LFWVHLA
 HYD51135  PWLTRN-EWKRSAVTDWRTD PDRWRWVIELLNLSLKAHLG SRGIAFDPDAKRFYFRPRKP GCSVR-LKWGSGTKR---TV VRAPDAEKGGN----WVHQA

 fna01588  GRLRFTILGEGIYLVIEPLR VL-TYDGTNPLDQKRNTRI- -STKKNFFYHNNNYLYDMKL WLHTLAGNREEIHLGYGSGK ITVDVTSINTKVNFGIFDDQ
MFC180375  GKLRFTFLGDQPYLIIEPTR VL-TVDGVEPLDQRRNVKI- -STRRNFHYHNNNYLYDMKL WLHILAGNREEIILGHGKEL IKVSITPLTSRINVGILNDQ
MFY930013  ARMRFIILGDGIYLTIEPIR VL-TEDGTNPLDQKRNTRI- -STKNNSWYHNNNYLYDIKL LLHILAGTKEEIHLGTGLGK ITVSILSINSKVNFGILGDL
TBR085681  ARMRFIILGGGIYLTIEPIR VL-TEDGSNPLDQRWNTRI- -STKKNSWYHNNNYLYDVKL MLHILAGNREEIHFGNGQGR ITVSILSINSKTNFGILNDL
MDR271946  GRLRFIILGSGVYLVIEPIR VL-TVDGQSPLDQNMNLRI- -STKKNFRYHNNNYLYDMKL WLHILAGNRQELHLGQGEGK ITISILSINSRVNFGILNDQ
MCL587675  GRLRFTILGNGVYLIIEPIR VL-TRDGLQPLDNRLNVRI- -STKKNVYYHNNNYLYDMKL WLHILAGNKEEVHLGQEPGK VIVSILSLTSKTSFGIAEDQ
MCW401937  ARLNFMYLNRLLFLRIDPTK VF-TEDGVKPINRDELAKL- -MSRYLSKEFNNLYLNSARF WAKYLSMLDINIRIPAGKQA IEIRTTPCSTKMPFG.....
MGB712338  RTDFHWIG-DTIHLRLEPGF LL-SEDGRHPLHGQKQGNVL ISLESWLSSHNAGYLRNVLF WSSRFQDTDKVIRLCPGLEV SGRPTEARV-GIGIRED...
WP1793690  KVSIVKLG-GKFFLKLNLTR VI-SDDGRNVSKGEREGTI- -ITRSGYNTFNNQYLNDVLF WINKLGNAEDVKVMDDFVIS IDPVQTTMNRGISWDIPTSE
 VVB64402  SSNFVQLGKGKFFLKILPTF II-TEDGYHVIKGLKEGTI- -ITRLSYNKYNNDFLNTIFF WIYQLGGGKDIIINDYLSIS SEPLKFDSEIGILFDIPSSE
MCF213827  VKLVAREIDSSLYIQISPRI VL-TSDGYKPIIDSTMGTK- -ITSLSYNEYNGQYLLNILF WI.................. ....................
 fna01260  GLR-FILLGVNAFLRIETGR VF-SIDGRRLIRGRRKTVL- -STRVLSRQRNLAEFDDTRF WAWLLSEDGYTIKMDLGGLY LEINTEPLSTTIRGGIFGDS
 WNZ28653  CKATFTSLGENIFLKVIPGF HF-TIDGLIKAVPNKFMSS- LSTRWMNPQKNHSVLDDVRF WIYKISEGTENAQINVGGTS PVLVRSTPLSAVIDRGIVED
MFX020972  CKATFTKIGDNLFLKVIPGW HF-TTDGLFNPVPPKMMTS- LSTKWMNIQRNHSILDDVRF WIQRLSLGNEYLELDVGADS PVIISSLPISSRIDHGIEED
MDG690461  KAT-FAYFKQVLCLKLLPGW HF-TTDGIQNPVSSQSMSS- LSSRWMNRERNHSILDDIRF WINTLAQGTDMVHLDVGSEI MTEISSVPIFASLDRGIEGD
 HWY27816  AVTRIIDIDDRLFLRIEPGW TF-TTNGVEPLPRDKIGSI- -STRWMFDEYNPSIFYHLRF WIFILS-RGKNGITLQAGKS EIEIENTPTTTEMHWGIEGD
 TMI14703  VVLRFILIESRIFLRMEPGW TF-TRDGVTPLPRREMQSI- -STRWMYDEYNPSIFYHLRF WTYILS-QAKRQIVLDAGGS SICIEPMPATALMKFGVEGD
 CUR52643  LQAKFLLVDNEIVLQIVPGW TF-TSDGEHAISPREIGSL- -SSKWTHNEYNSSVFYHIRF WCFILSKGQEKISMKLAHDL SLEVDITPAVSDISVGIEGD
MCE965209  LQAKFLLLDGEIFLQIVPGW TF-TSDGEHSISPREIGSL- -SSKWTHNEYNSSVLYHVRF WSFILSKGQEKISMKLAENS QITIDITPAVSDISVGIDGD
MFQ591027  LRARFTILESDPYLVISPSW FH-TVDGVEPVPIERRGPL- -ITRRIYREGNGRVLSNLRL WLAYLGAGTESIKMRT.... ....................
 HYD51135  ARLSFETLGNRLFLAVEPCW VF-TTDGRNSVRKQDIGPL- -AMQWGGKERNGAIVRHVLM WSDVLT-NGHRTGHIPCGDK PIVIGRLPATVGMGVGIADD

 fna01588  HTSEDFLDTLKS
MFC180375  HTNEDFLDNLKS
MFY930013  HTGEDFLDSLKS
TBR085681  HAGGDFLDSLKS
MDR271946  HTKGDFLDALKS
MCL587675  HTGADFLDSLKS
MCW401937  ............
MGB712338  ............
WP1793690  ............
 VVB64402  FKLD........
MCF213827  ............
 fna01260  ............
 WNZ28653  ............
MFX020972  ............
MDG690461  ............
 HWY27816  ............
 TMI14703  ............
 CUR52643  ............
MCE965209  ............
MFQ591027  ............
 HYD51135  ............
```
